# Supplementary material for: Synthesis and Inhibitory Studies of Phosphonic Acid Analogues of Homophenylalanine and Phenylalanine towards Alanyl Aminopeptidases
Source: Biomolecules. 2020 Sep 14;10(9):1319. doi: 10.3390/biom10091319 (PMC7565091; doi:10.3390/biom10091319)
Supplement: Supplementary file 1 [file biomolecules-10-01319-s001.pdf]

biomolecules

MDPI

Article

Synthesis and inhibitory studies of phosphonic acid analogues of homophenylalanine and phenylalanine towards Alanyl Aminopeptidases

Supplementary Materials

Table of Contents

Section S1. The characterization data of the compounds **2c-2h** and **8a-8e**. 3

Section S2. The characterization data of the compounds **3c-3h** and **9a-9e**. 4

Section S3. The characterization data of the compounds **4e-4h, 10a** and **10b**....6

Section S4. The characterization data of the compounds **4b, 4c, 10c-10e**....7

Section S5. The characterization data of the compounds **6b-6h and 13a-13e**. 8

Section S6. The characterization data of the compounds **14c, 14f, 14h, 16d and 16e**. 11

Figure S1. ^{1}H (**A**), ^{13}C (**B**) and ^{19}F (**C**) NMR spectra for 3-(4-fluorophenyl)propyl-3-(4-fluorophenyl) propionate (**5d**). 13

Figure S2. ^{1}H (**A**), ^{1}H-^{31}P HMQC (**B**) and ^{1}H-^{13}C HMQC (**C**) NMR spectra for 1-amino-3-(4-fluorophenyl)propylphosphonic acid (**15d**). 14

Section S7. Molecular docking simulations of the inhibitors **15c, 15f, 17b** and **17c** binding to active site of pAPN (PDB: 4FKE). 16

Figure S7-1. Binding mode of the 1-amino-3-(3-fluorophenyl)propylphosphonic acid (compound **15c**) with the pAPN 16

Figure S7-2. Binding mode of the 1-amino-3-(3,4-difluorophenyl)propylphosphonic acid (compound **15f**) with the pAPN 16

Figure S7-3. Binding mode of the 1-amino-2-(2-bromo-5-fluorophenyl)ethylphosphonic acid (compound **17b**) with the pAPN 17

Figure S7-4. Binding mode of the 1-amino-2-(3-bromo-4-fluorophenyl)ethylphosphonic acid (compound **17c**) with the pAPN 17

Section S8. Molecular docking simulations of the inhibitors **15f, 15g** and **17c** binding to active site of hAPN (PDB: 4FYT). 18

Figure S8-1. Binding mode of the 1-amino-3-(3,4-difluorophenyl)propylphosphonic acid (compound **15f**) with the hAPN 18

Figure S8-2. Binding mode of the 1-amino-3-(4-trifluoromethylphenyl)propylphosphonic acid (compound **15g**) with the hAPN 18

Figure S8-3. Binding mode of the 1-amino-2-(3-bromo-4-fluorophenyl)ethylphosphonic acid (compound **17c**) with the hAPN 19

Section S9. X-Ray analysis of compounds **13a, 13c** and **14c** 19

Figure S9. Molecular structures of (**13a**)(**A**), (**13c**) (**B**) and (**14c**) (**C**) in the asymmetric part of unit cell 20

Table S9-1. Crystal parameters and experimental details of the X-Ray data collection for structure **13a, 13c** and **14c**. 21

Table S9-2. Selected geometric parameters for crystal structure **13a** (Å, º). 22

Table S9-3. Selected hydrogen-bond parameters for structure **13a** 25

Table S9-4. Selected geometric parameters for crystal structure **13c** (Å, º). 25

Table S9-5. Selected geometric parameters for crystal structure **14c** (Å, º). 29

Table S9-6. Selected hydrogen-bond parameters for structure **14c** 31

Section S10. Characterization of the Final Compounds **15a-15h** and **17a-17e** by ^{1}H, ^{13}C, ^{19}F, ^{31}P NMR. 32

Figure S10-1. ^{1}H (**A**), ^{31}P (**B**) NMR spectra for compound **15a**. 32

Figure S10-2. ^{1}H (**A**),^{13}C (**B**), ^{19}F (**C**), ^{31}P (**D**) NMR spectra for compound **15b**. 33

Figure S10-3. ^{1}H (**A**),^{13}C (**B**), ^{19}F (**C**), ^{31}P (**D**) NMR spectra for compound **15c**. 35

Figure S10-4. ^{1}H (**A**),^{13}C (**B**), ^{19}F (**C**), ^{31}P (**D**) NMR spectra for compound **15d**. 37

Figure S10-5. ^{1}H (**A**),^{13}C (**B**), ^{19}F (**C**), ^{31}P (**D**) NMR spectra for compound **15e** 39

Figure S10-6. ^{1}H (**A**),^{13}C (**B**), ^{19}F (**C**), ^{31}P (**D**) NMR spectra for compound **15f**. 41

Figure S10-7. ^{1}H (**A**),^{13}C (**B**), ^{19}F (**C**), ^{31}P (**D**) NMR spectra for compound **15g** 43

Figure S10-8. ^{1}H (**A**),^{13}C (**B**), ^{19}F (**C**), ^{31}P (**D**) NMR spectra for compound **15h**. 45

Figure S10-9. ^{1}H (**A**),^{13}C (**B**), ^{19}F (**C**), ^{31}P (**D**) NMR spectra for compound **17a** 47

Figure S10-10. ^{1}H (**A**), ^{19}F (**B**), ^{31}P (**C**) NMR spectra for compound **17b**. 49

Figure S10-11. ^{1}H (**A**),^{13}C (**B**), ^{19}F (**C**), ^{31}P (**D**) NMR spectra for compound **17c**. 51

Figure S10-12. ^{1}H (**A**),^{13}C (**B**), ^{19}F (**C**), ^{31}P (**D**) NMR spectra and HPLC (**E**) for compound **17d**. 53

Figure S10-13. ^{1}H (**A**),^{13}C (**B**), ^{19}F (**C**), ^{31}P (**D**) NMR spectra for compound **17e** 55

Section S1.** The characterization data of the compounds* ***2c-2h *and* ***8a-8e****.*

3-(3-fluorophenyl)propionic acid methyl ester (**2c**) [1]

Yellow oil, yield 100%; ^{1}H NMR (400 MHz, $CDCl_3$), δ = 7.27 – 7.18 (m, 1H, CH_{ar}), 6.96 (d, *J* = 7.8 Hz, 1H, CH_{ar}), 6.88 (ddd, *J* = 6.5, 2.4, 0.4 Hz, 1H, CH_{ar}), 3.66 (s, 3H, OCH_3), 2.94 (t, *J* = 7.7 Hz, 2H, CH_2), 2.62 (t, *J* = 7.8 Hz, 2H, CH_2) ppm; ^{13}C NMR (101 MHz, $CDCl_3$), δ = 173.10 (s, **C**$OOCH_3$), 162.99 (d, *J* = 245.6 Hz, C_{ar}-F), 143.10 (d, *J* = 7.3 Hz, C_{ar}), 130.02 (d, *J* = 8.3 Hz, C_{ar}), 124.01 (d, *J* = 2.8 Hz, C_{ar}), 115.26 (d, *J* = 21.1 Hz, C_{ar}), 113.27 (d, *J* = 21.0 Hz, C_{ar}), 51.77 (s, COO**C**H_3), 35.39 (s, CH_2), 30.67 (d, *J* = 1.8 Hz, CH_2) ppm; ^{19}F NMR (376 MHz, $CDCl_3$), δ = -113.30 – -113.38 (m, 1F) ppm.

3-(4-fluorophenyl)propionic acid methyl ester (**2d**) [1,2]

Yellow oil, yield 100%; ^{1}H NMR (400 MHz, $CDCl_3$), δ = 7.14 (ddd, *J* = 8.3, 5.4, 0.5 Hz, 2H, 2xCH_{ar}), 6.99 – 6.92 (m, 2H, 2xCH_{ar}), 3.65 (s, 3H, OCH_3), 2.91 (t, *J* = 7.7 Hz, 2H, CH_2), 2.59 (t, *J* = 7.7 Hz, 2H, CH_2) ppm; ^{13}C NMR (101 MHz, $CDCl_3$), δ = 173.24 (s, **C**$OOCH_3$), 161.57 (d, *J* = 244.1 Hz, C_{ar}-F), 136.20 (d, *J* = 3.2 Hz, C_{ar}), 129.78 (d, *J* = 7.9 Hz, 2xC_{ar}), 115.34 (d, *J* = 21.2 Hz, 2xC_{ar}), 51.71 (s, COO**C**H_3), 35.85 (d, *J* = 1.1 Hz, CH_2), 30.19 (d, *J* = 0.7 Hz, CH_2) ppm; ^{19}F NMR (376 MHz, $CDCl_3$), δ = -116.94 (tt, J = 8.7, 5.2 Hz, 1F) ppm.

3-(2,4-difluorophenyl)propionic acid methyl ester (**2e**)

Yellow oil, yield 100%; ^{1}H NMR (400 MHz, $CDCl_3$), δ = 7.16 (ddd, *J* = 8.5, 6.9, 3.5 Hz, 1H, CH_{ar}), 6.81 – 6.73 (m, 2H, 2xCH_{ar}), 3.65 (s, 3H, OCH_3), 2.92 (t, *J* = 7.6 Hz, 2H, CH_2), 2.60 (t, *J* = 7.6 Hz, 2H, CH_2) ppm; ^{13}C NMR (101 MHz, $CDCl_3$), δ = 173.07 (s, **C**$OOCH_3$), 161.43 (ddd, *J* = 83.7, 71.4, 11.9 Hz, 2xC_{ar}-F), 131.24 (dd, *J* = 9.5, 6.5 Hz, C_{ar}), 123.18 (dd, *J* = 15.8, 3.8 Hz, C_{ar}), 111.13 (dd, *J* = 20.9, 3.8 Hz, C_{ar}), 103.84 (dd, *J* = 26.0, 25.3 Hz, C_{ar}), 51.77 (s, COO**C**H_3), 34.25 (t, *J* = 1.4 Hz, CH_2), 24.13 (d, *J* = 2.3 Hz, CH_2) ppm; ^{19}F NMR (376 MHz, $CDCl_3$), δ = -112.70 – -112.83 (m, 1F), -114.18 (ddd, *J* = 15.8, 8.7, 3.8 Hz, 1F) ppm.

3-(3,4-difluorophenyl)propionic acid methyl ester (**2f**)

Yellow oil, yield 100%; ^{1}H NMR (400 MHz, $CDCl_3$), δ = 7.09 – 6.95 (m, 2H, 2xCH_{ar}), 6.92 – 6.86 (m, 1H, CH_{ar}), 3.66 (s, 3H, OCH_3), 2.89 (t, *J* = 7.6 Hz, 2H, CH_2), 2.59 (t, *J* = 7.6 Hz, 2H, CH_2) ppm; ^{13}C NMR (101 MHz, $CDCl_3$), δ = 172.95 (s, **C**$OOCH_3$), 149.66 (ddd, *J* = 129.2, 117.3, 12.6 Hz, 2xC_{ar}-F), 137.47 (dd, *J* = 5.6, 3.9 Hz, C_{ar}), 124.24 (dd, *J* = 6.1, 3.5 Hz, C_{ar}), 117.23 (ddd, *J* = 16.9, 2.4, 0.6 Hz, 2xC_{ar}), 51.81 (s, COO**C**H_3), 35.46 (d, *J* = 1.0 Hz, CH_2), 30.12 (d, *J* = 1.4 Hz, 30.12 (d, *J* = 1.4 Hz) ppm; ^{19}F NMR (376 MHz, $CDCl_3$), δ = -137.88 – -137.99 (m, 1F), -141.39 – -141.51 (m, 1F) ppm.

3-(4-trifluoromethylphenyl)propionic acid methyl ester (**2g**) [1a,1b,3,4]

Yellow oil, yield 100%; ^{1}H NMR (400 MHz, $CDCl_3$), δ = 7.42 (dd, *J* = 91.2, 8.2 Hz, 4H, 4xCH_{ar}), 3.66 (s, 3H, OCH_3), 3.00 (t, *J* = 7.7 Hz, 2H, CH_2), 2.64 (t, *J* = 7.7 Hz, 2H, CH_2) ppm; ^{13}C NMR (101 MHz, $CDCl_3$), δ = 172.97 (s, **C**$OOCH_3$), 144.64 (q, *J* = 1.3 Hz, C_{ar}), 128.73 (s, 2xC_{ar}), 125.53 (q, *J* = 3.8 Hz, C_{ar}-CF_3), 125.23 (s, C_{ar}), 125.06 (s, C_{ar}) 124.32 (q, *J* = 271.8 Hz, C_{ar}-CF_3), 51.83 (s, COO**C**H_3), 35.85 (d, *J* = 0.5 Hz, CH_2), 30.19 (s, CH_2) ppm; ^{19}F NMR (376 MHz, $CDCl_3$), δ = -62.32 (s, 3F, CF_3) ppm.

3-(2-trifluoromethylphenyl)propionic acid methyl ester (**2h**) [1a,5]

Yellow oil, yield 100%; ^{1}H NMR (400 MHz, $CDCl_3$), δ = 7.62 (d, *J* = 7.9 Hz, 1H, CH_{ar}), 7.46 (t, *J* = 7.6 Hz, 1H, CH_{ar}), 7.31 (dd, *J* = 16.4, 7.8 Hz, 2H, 2xCH_{ar}), 3.68 (s, 3H, OCH_3), 3.13 (t, *J* = 8.7 Hz, 2H, CH_2), 2.62 (t, *J* = 7.8 Hz, 2H, CH_2) ppm; ^{13}C NMR (101 MHz, $CDCl_3$), δ = 173.35 (s, **C**$OOCH_3$), 139.28 (q, *J* = 1.7 Hz, C_{ar}), 132.03 (q, *J* = 1.1 Hz, C_{ar}), 131.02 (s, C_{ar}), 128.66 (q, *J* = 29.8 Hz, C_{ar}-CF_3), 126.57 (s, C_{ar}), 126.19 (q, *J* = 5.7 Hz, C_{ar}), 124.59 (q, *J* = 273.7 Hz, C_{ar}-CF_3), 51.81 (s, COO**C**H_3), 35.70 (q, *J* = 1.1 Hz, CH_2), 30.19 (q, *J* = 1.9 Hz, CH_2) ppm; ^{19}F NMR (376 MHz, $CDCl_3$), δ = -59.72 (s, 3F, CF_3) ppm.

2-(2-bromo-4-fluorophenyl)acetic acid methyl ester (8a) [6]

Yellow oil, yield 100%; ^{1}H NMR (400 MHz, $CDCl_3$), δ = 7.31 (dd, *J* = 8.2, 2.7 Hz, 1H, CH_{ar}), 7.25 (dd, *J* = 8.6, 5.9 Hz, 1H, CH_{ar}), 7.00 (td, *J* = 8.3, 2.6 Hz, 1H, CH_{ar}), 3.75 (s, 2H, CH_2), 3.71 (s, 3H, OCH_3) ppm; ^{13}C NMR (101 MHz, $CDCl_3$), δ = 170.91 (d, *J* = 1.4 Hz, **C**$OOCH_3$), 161.64 (d, *J* = 250.4 Hz, C_{ar}-F), 132.31 (d, *J* = 8.5 Hz, C_{ar}), 130.23 (d, *J* = 3.7 Hz, C_{ar}), 125.03 (d, *J* = 9.6 Hz, C_{ar}), 120.14 (d, *J* = 24.5 Hz, C_{ar}), 114.80 (d, *J* = 21.0 Hz, C_{ar}), 52.33 (s, COO**C**H_3), 40.69 (s, 2H,CH_2) ppm; ^{19}F NMR (376 MHz, $CDCl_3$), δ = -112.92 – -112.99 (m, 1F) ppm.

2-(2-bromo-5-fluorophenyl)acetic acid methyl ester (**8b**) [7]

Yellow oil, yield 100%; ^{1}H NMR (400 MHz, $CDCl_3$), δ = 7.50 (dd, *J* = 8.8, 5.3 Hz, 1H, CH_{ar}), 7.03 (dd, *J* = 9.0, 3.0 Hz, 1H, CH_{ar}), 6.87 (ddd, *J* = 8.5, 8.1, 3.0 Hz, 1H, CH_{ar}), 3.76 (s, 2H, CH_2), 3.72 (s, 3H, OCH_3) ppm; ^{13}C NMR (101 MHz, $CDCl_3$), δ = 170.45 (d, *J* = 0.5 Hz, **C**$OOCH_3$), 161.87 (d, *J* = 247.1 Hz, C_{ar}-F), 136.11 (d, *J* = 8.0 Hz, C_{ar}), 134.00 (d, *J* = 8.1 Hz, C_{ar}), 119.21 (d, *J* = 3.4 Hz, C_{ar}), 118.60 (d, *J* = 23.2 Hz, C_{ar}), 116.19 (d, *J* = 22.4 Hz, C_{ar}), 52.40 (s, CH_2), 41.52 (d, *J* = 1.4 Hz, COO**C**H_3) ppm; ^{19}F NMR (376 MHz, $CDCl_3$), δ = -114.57 – -114.64 (m, 1F) ppm.

2-(3-bromo-4-fluorophenyl)acetic acid methyl ester (**8c**) [8]

Yellow oil, yield 100%; ^{1}H NMR (400 MHz, $CDCl_3$), δ = 7.47 (dd, *J* = 6.5, 2.2 Hz, 1H, CH_{ar}), 7.18 (ddd, *J* = 8.2, 4.6, 2.2 Hz, 1H, CH_{ar}), 7.06 (t, *J* = 8.4 Hz, 1H, CH_{ar}), 3.69 (s, 3H, OCH_3), 3.57 (s, 2H, CH_2) ppm; ^{13}C NMR (101 MHz, $CDCl_3$), δ = 171.39 (d, *J* = 1.3 Hz, **C**$OOCH_3$), 158.43 (d, *J* = 247.0 Hz, C_{ar}-F), 134.34 (d, *J* = 0.7 Hz, C_{ar}), 131.31 (d, *J* = 4.0 Hz, C_{ar}), 129.96 (d, *J* = 7.3 Hz, C_{ar}), 116.53 (d, *J* = 22.4 Hz, C_{ar}), 109.08 (d, *J* = 21.2 Hz, C_{ar}), 52.34 (s, CH_2), 39.95 (s, COO**C**H_3) ppm; ^{19}F NMR (376 MHz, $CDCl_3$), δ = -109.68 (dd, *J* = 12.9, 6.5 Hz, 1F) ppm.

2-(4-bromo-2-fluorophenyl)acetic acid methyl ester (**8d**) [9]

Yellow oil, yield 100%; ^{1}H NMR (400 MHz, $CDCl_3$), δ = 7.26 – 7.21 (m, 2H, 2xCH_{ar}), 7.13 (t, *J* = 8.1 Hz, 1H, CH_{ar}), 3.69 (s, 2H, CH_2), 3.61 (s, 2H, OCH_3) ppm; ^{13}C NMR (101 MHz, $CDCl_3$), δ = 170.69 (d, *J* = 1.1 Hz, **C**$OOCH_3$), 160.90 (d, *J* = 251.3 Hz, C_{ar}-F), 132.59 (d, *J* = 4.6 Hz, C_{ar}), 127.56 (d, *J* = 3.8 Hz, C_{ar}), 121.48 (d, *J* = 9.5 Hz, C_{ar}), 120.62 (d, *J* = 16.0 Hz, C_{ar}), 119.19 (d, *J* = 25.1 Hz, C_{ar}), 52.39 (s, CH_2), 33.96 (d, *J* = 2.9 Hz, COO**C**H_3) ppm; ^{19}F NMR (376 MHz, $CDCl_3$), δ = -114.12 (dd, *J* = 12.8, 4.8 Hz, 1F) ppm.

2-(4-bromo-3-fluorophenyl)acetic acid methyl ester (**8e**) [10]

Yellow oil, yield 100%; ^{1}H NMR (400 MHz, $CDCl_3$), δ = 7.48 (dd, *J* = 7.9, 7.4 Hz, 1H, CH_{ar}), 7.06 (dd, *J* = 9.3, 2.0 Hz, 1H, CH_{ar}), 6.94 (dd, *J* = 8.2, 2.0 Hz, 1H, CH_{ar}), 3.69 (s, 3H, OCH_3), 3.58 (s, 2H, CH_2) ppm; ^{13}C NMR (101 MHz, $CDCl_3$), δ = 171.06 (d, *J* = 0.5 Hz, **C**$OOCH_3$), 159.03 (d, *J* = 247.5 Hz, C_{ar}-F), 135.49 (d, *J* = 7.1 Hz, C_{ar}), 133.56 (d, *J* = 0.8 Hz, C_{ar}), 126.34 (d, *J* = 3.6 Hz, C_{ar}), 117.63 (d, *J* = 22.7 Hz, C_{ar}), 107.82 (d, *J* = 20.8 Hz, C_{ar}), 52.38 (s, CH_2), 40.43 (d, *J* = 1.6 Hz, COO**C**H_3) ppm; ^{19}F NMR (376 MHz, $CDCl_3$), δ = -107.03 (dd, *J* = 9.3, 7.2 Hz, 1F) ppm.

Section S2. *The characterization data of the compounds* ***3c-3h*** *and* ***9a-9e****.*

3-(3-fluorophenyl)propanol (**3c**) [1b,11]

Colourless oil, yield 100%; ^{1}H NMR (400 MHz, $CDCl_3$), δ = 7.23 (ddd, *J* = 13.9, 4.9, 3.8 Hz, 1H, CH_{ar}), 6.96 (d, *J* = 7.6 Hz, 1H, CH_{ar}), 6.89 (ddd, *J* = 13.9, 6.4, 4.9 Hz, 2H, CH_{ar}), 3.66 (t, *J* = 6.4 Hz, 2H, CH_2), 2.73 – 2.66 (m, 2H, CH_2), 1.87 (dt, *J* = 13.7, 6.5 Hz, 2H, CH_2), 1.68 (s, 1H, OH) ppm; ^{13}C NMR (101 MHz, $CDCl_3$), δ = 163.02 (d, *J* = 245.2 Hz, C_{ar}-F), 144.48 (d, *J* = 7.2 Hz, C_{ar}), 129.86 (d, *J* = 8.3 Hz, C_{ar}), 124.16 (d, *J* = 2.7 Hz, C_{ar}), 115.32 (d, *J* = 20.8 Hz, C_{ar}), 112.82 (d, *J* = 21.0 Hz, C_{ar}), 62.10 (s, **C**H_2OH), 33.94 (s, CH_2-C_{ar}), 31.86 (d, *J* = 1.7 Hz, CH_2**C**H_2CH_2OH) ppm; ^{19}F NMR (376 MHz, $CDCl_3$), δ = -113.30 – -113.39 (m, 1F) ppm.

3-(4-fluorophenyl)propanol (**3d**) [12,13]

Colourless oil, yield 100%; ^{1}H NMR (400 MHz, $CDCl_3$), δ = 7.14 (ddd, *J* = 8.3, 5.4, 0.5 Hz, 2H, 2xCH_{ar}), 6.95 (t, *J* = 8.8 Hz, 2H, 2xCH_{ar}), 3.66 (t, *J* = 6.4 Hz, 2H, CH_2), 2.70 – 2.65 (m, 2H, CH_2), 1.89 – 1.82 (m, 2H, CH_2), 1.46 (s, 1H, OH) ppm; ^{13}C NMR (101 MHz, $CDCl_3$), δ = 161.35 (d, *J* = 243.3 Hz, C_{ar}-F), 137.45 (d, *J* = 3.2 Hz, C_{ar}), 129.85 (s, C_{ar}), 129.77 (s, C_{ar}), 115.29 (s, C_{ar}), 115.08 (s, C_{ar}), 62.15 (s, CH_2OH), 34.37 (d, *J* = 1.0 Hz, CH_2-C_{ar}), 31.29 (d, *J* = 0.5 Hz, CH_2**C**H_2CH_2OH) ppm; ^{19}F NMR (376 MHz, $CDCl_3$), δ = -117.64 (tt, *J* = 8.7, 5.2 Hz, 1F) ppm.

3-(2,4-difluorophenyl)propanol (**3e**) [14]

Colourless oil, yield 100%; ^{1}H NMR (400 MHz, $CDCl_3$), δ = 7.17 – 7.11 (m, 1H, CH_{ar}), 6.81 – 6.73 (m, 2H, 2xCH_{ar}), 3.65 (t, *J* = 6.4 Hz, 2H, CH_2), 2.69 (t, *J* = 7.7 Hz, 2H, CH_2), 1.87 – 1.80 (m, 2H, CH_2), 1.63 (s, 1H, OH) ppm; ^{13}C NMR (101 MHz, $CDCl_3$), δ = 161.26 (ddd, *J* = 55.0, 42.7, 11.8 Hz, C_{ar}-F), 131.18 (dd, *J* = 9.4, 6.7 Hz, C_{ar}), 124.40 (dd, *J* = 16.2, 3.8 Hz, C_{ar}), 111.04 (dd, *J* = 20.9, 3.8 Hz, C_{ar}), 103.71 (dd, *J* = 26.4, 25.1 Hz, C_{ar}), 62.04 (s, CH_2OH), 32.98 (d, *J* = 1.1 Hz, CH_2-C_{ar}), 24.83 (d, *J* = 2.1 Hz, CH_2**C**H_2CH_2OH) ppm; ^{19}F NMR (376 MHz, $CDCl_3$), δ = -113.58 (ddd, *J* = 15.1, 8.4, 6.7 Hz, 1F), -114.54 (dd, *J* = 16.2, 8.7 Hz, 1F) ppm.

3-(3,4-difluorophenyl)propanol (**3f**) [15]

Colourless oil, yield 100%; ^{1}H NMR (400 MHz, $CDCl_3$), δ = 7.07 – 6.94 (m, 2H, 2xCH_{ar}), 6.89 – 6.85 (m, 1H, CH_{ar}), 3.64 (t, *J* = 6.4 Hz, 2H, CH_2), 2.71 – 2.57 (m, 2H, CH_2), 2.33 (s, 1H, OH), 1.87 – 1.80 (m, 2H, CH_2) ppm; ^{13}C NMR (101 MHz, $CDCl_3$), δ = 149.51 (ddd, *J* = 154.8, 143.1, 12.6 Hz, C_{ar}-F), 138.80 (dd, *J* = 5.4, 3.9 Hz, C_{ar}), 124.25 (dd, *J* = 6.0, 3.5 Hz, 2xC_{ar}), 117.15 (d, *J* = 16.7 Hz, C_{ar}), 117.05 (dd, *J* = 16.9, 0.8 Hz, C_{ar}), 61.84 (s, CH_2OH), 33.92 (s, CH_2-C_{ar}), 31.26 (d, *J* = 1.3 Hz, CH_2**C**H_2CH_2OH) ppm; ^{19}F NMR (376 MHz, $CDCl_3$), δ = -138.34 – -138.45 (m, 1F), -142.18 – -142.31 (m, 1F) ppm.

3-(4-trifluoromethylphenyl)propanol (**3g**) [1b,16,17]

Colourless oil, yield 100%; ^{1}H NMR (400 MHz, $CDCl_3$), δ = 7.41 (dd, *J* = 91.1, 7.9 Hz, 4H, 4xCH_{ar}), 3.67 (t, *J* = 6.4 Hz, 2H, CH_2), 2.78 – 2.74 (m, 2H, CH_2), 1.93 – 1.85 (m, 2H, CH_2), 1.55 (s, 1H, OH) ppm; ^{13}C NMR (101 MHz, $CDCl_3$), δ = 146.05 (q, *J* = 1.3 Hz, C_{ar}), 128.82 (s, 2xC_{ar}); 128.35 (q, *J* = 32.3 Hz, CF3-C_{ar}), 125.39 (q, *J*= 3.8 Hz, 2xC_{ar}), 124.42 (q, *J* = 271.0 Hz, CF_3-C_{ar}); 62.00 (s, CH_2OH), 33.92 (s, CH_2-C_{ar}), 31.96 (s, CH_2**C**H_2CH_2OH) ppm; ^{19}F NMR (376 MHz, $CDCl_3$), δ = -62.22 (s, 3F, CF_3) ppm.

3-(2-trifluoromethylphenyl)propanol (**3h**) [18,19]

Colourless oil, yield 100%; ^{1}H NMR (400 MHz, $CDCl_3$), δ = 7.61 (d, *J* = 7.9 Hz, 1H, CH_{ar}), 7.46 (t, *J* = 7.3 Hz, 1H, CH_{ar}), 7.34 (d, *J* = 7.7 Hz, 1H, CH_{ar}), 7.28 (t, *J* = 7.6 Hz, 1H, CH_{ar}), 3.71 (t, *J* = 6.4 Hz, 2H, CH_2), 2.86 (dd, *J* = 12.0, 3.9 Hz, 2H, CH_2), 1.89 (ddd, *J* = 14.3, 10.3, 6.3 Hz, 2H, CH_2), 1.66 (s, 1H, OH) ppm; ^{13}C NMR (101 MHz, $CDCl_3$), δ = 140.79 (q, *J* = 1.7 Hz, C_{ar}), 131.84 (q, *J* = 1.1 Hz, C_{ar}), 131.14 (s, C_{ar}), 128.52 (q, *J* = 29.6 Hz, **C**$_{ar}$-CF_3), 126.06 (q, *J* = 5.8 Hz, C_{ar}), 126.05 (s, C_{ar}), 124.72 (q, J = 273.8 Hz, C_{ar}-**C**F_3), 62.45 (s, CH_2OH), 34.59 (d, *J* = 0.4 Hz, CH_2-C_{ar}), 28.98 (q, *J* = 1.8 Hz, CH_2**C**H_2CH_2OH) ppm; ^{19}F NMR (376 MHz, $CDCl_3$), δ = -59.82 (s, 3F, CF_3) ppm.

2-(2-bromo-4-fluorophenyl)ethanol (**9a**) [20,21]

Colourless oil, yield 100%; ^{1}H NMR (400 MHz, $CDCl_3$), δ = 7.29 (dd, *J* = 8.2, 2.7 Hz, 1H, CH_{ar}), 7.24 (dd, *J* = 8.5, 6.0 Hz, 1H, CH_{ar}), 6.97 (td, *J* = 8.3, 2.7 Hz, 1H, CH_{ar}), 3.85 (t, *J* = 6.7 Hz, 2H, CH_2), 2.98 (t, *J* = 6.7 Hz, 2H, CH_2), 1.62 (s, 1H, OH) ppm; ^{13}C NMR (101 MHz, $CDCl_3$), δ = 161.24 (d, *J* = 249.2 Hz, C_{ar}-F), 133.79 (d, *J* = 3.5 Hz, C_{ar}), 131.98 (d, *J* = 8.3 Hz, C_{ar}), 124.48 (d, *J* = 9.4 Hz, C_{ar}), 120.13 (d, *J* = 24.3 Hz, C_{ar}), 114.63 (d, *J* = 20.7 Hz, C_{ar}), 62.45 (d, *J* = 1.4 Hz, CH_2OH), 38.52 (s, CH_2-C_{ar}) ppm; ^{19}F NMR (376 MHz, $CDCl_3$), δ = -114.24 (td, *J* = 8.2, 6.1 Hz, 1F) ppm.

2-(2-bromo-5-fluorophenyl)ethanol (**9b**) [20,22]

Colourless oil, yield 100%; ^{1}H NMR (400 MHz, $CDCl_3$), δ = 7.48 (dd, *J* = 8.8, 5.4 Hz, 1H, CH_{ar}), 7.01 (dd, *J* = 9.2, 3.0 Hz, 1H, CH_{ar}), 6.82 (ddd, *J* = 8.8, 7.9, 3.1 Hz, 1H, CH_{ar}), 3.87 (t, *J* = 6.6 Hz, 2H, CH_2), 2.98 (t, *J* = 6.6 Hz, 2H, CH_2), 1.63 (s, 1H, OH) ppm; ^{13}C NMR (101 MHz, $CDCl_3$), δ = 161.94 (d, *J* = 247.0 Hz, C_{ar}-F), 140.13 (d, *J* = 7.5 Hz, C_{ar}), 134.03 (d, *J* = 8.1 Hz, C_{ar}), 118.76 (d, *J* = 3.2 Hz, C_{ar}), 118.18 (d, *J* = 22.5 Hz, C_{ar}), 115.39 (d, *J* = 22.4 Hz, C_{ar}), 61.80 (d, *J* = 0.6 Hz, CH_2OH), 39.38 (d, *J* = 1.3 Hz, CH_2-C_{ar}) ppm; ^{19}F NMR (376 MHz, $CDCl_3$), δ = -114.92 – -114.64 (td, *J* = 14.0, 8.5, 5.5 Hz, 1F) ppm.

2-(3-bromo-4-fluorophenyl)ethanol (**9c**) [23]

Colourless oil, yield 100%; ^{1}H NMR (400 MHz, $CDCl_3$), δ = 7.41 (dd, *J* = 6.6, 2.1 Hz, 1H, CH_{ar}), 7.14 – 7.10 (m, 1H, CH_{ar}), 7.04 (t, *J* = 8.4 Hz, 1H, CH_{ar}), 3.83 (t, *J* = 6.5 Hz, 2H, CH_2), 2.80 (t, *J* = 6.5 Hz, 2H, CH_2), 1.63 (s, 1H, OH) ppm; ^{13}C NMR (101 MHz, $CDCl_3$), δ = 157.97 (d, *J* = 245.8 Hz, C_{ar}-F), 136.17 (d, *J* = 3.9 Hz, C_{ar}), 133.89 (s, C_{ar}), 129.55 (d, *J* = 7.0 Hz, C_{ar}), 116.46 (d, *J* = 22.1 Hz, C_{ar}), 108.98 (d, *J* = 20.9 Hz, C_{ar}), 63.39 (d, *J* = 1.4 Hz, CH_2OH), 38.04 (s, CH_2-C_{ar}) ppm; ^{19}F NMR (376 MHz, $CDCl_3$), δ = -110.93 (ddd, *J* = 8.3, 6.7, 5.0 Hz, 1F) ppm.

2-(4-bromo-2-fluorophenyl)ethanol (**9d**) [24,25]

Colourless oil, yield 100%; ^{1}H NMR (400 MHz, $CDCl_3$), δ = 7.23 – 7.19 (m, 2H, 2xCH_{ar}), 7.13 (t, *J* = 7.9 Hz, 1H, CH_{ar}), 3.83 (t, *J* = 6.6 Hz, 2H, CH_2), 2.85 (td, *J* = 6.6, 0.9 Hz, 2H, CH_2), 1.57 (s, 1H, OH) ppm; ^{13}C NMR (101 MHz, $CDCl_3$), δ = 161.20 (d, *J* = 249.8 Hz, C_{ar}-F), 132.53 (d, *J* = 5.6 Hz, C_{ar}), 127.43 (d, *J* = 3.7 Hz, C_{ar}), 124.78 (d, *J* = 16.0 Hz, C_{ar}), 120.42 (d, *J* = 9.6 Hz, C_{ar}), 119.11 (d, *J* = 25.6 Hz, C_{ar}), 62.19 (d, *J* = 1.3 Hz, CH_2OH), 32.23 (d, *J* = 1.4 Hz, CH_2-C_{ar}) ppm; ^{19}F NMR (376 MHz, $CDCl_3$), δ = -115.37 (t, *J* = 8.7 Hz, 1F) ppm.

2-(4-bromo-3-fluorophenyl)ethanol (**9e**) [9,25]

Colourless oil, yield 100%; ^{1}H NMR (400 MHz, $CDCl_3$), δ = 7.45 (dd, *J* = 8.0, 7.3 Hz, 1H, CH_{ar}), 7.06 (dd, *J* = 9.5, 2.0 Hz, 1H, CH_{ar}), 6.89 (dd, *J* = 8.2, 1.9 Hz, 1H, CH_{ar}), 3.84 (t, *J* = 6.5 Hz, 2H, CH_2), 2.82 (dd, *J* = 6.4 Hz, 2H, CH_2), 1.64 (s, 1H, OH) ppm; ^{13}C NMR (101 MHz, $CDCl_3$), δ = 159.10 (d, *J* = 247.3 Hz, C_{ar}-F), 140.63 (d, *J* = 6.7 Hz, C_{ar}), 133.48 (d, *J* = 0.8 Hz, C_{ar}), 126.03 (d, *J* = 3.4 Hz, C_{ar}), 117.17 (d, *J* = 21.9 Hz, C_{ar}), 106.78 (d, *J* = 20.8 Hz, C_{ar}), 63.13 (d, *J* = 0.5 Hz, CH_2OH), 38.48 (d, *J* = 1.5 Hz, CH_2-C_{ar}) ppm; ^{19}F NMR (376 MHz, $CDCl_3$), δ = -107.51 (dd, *J* = 9.5, 7.2 Hz, 1F) ppm.

Section S3. *The characterization data of the compounds* ***4e-4h, 10a*** *and* ***10b****.*

3-(2,4-difluorophenyl)propanal (**4e**) [26]

Colourless oil, yield 62%; ^{1}H NMR (400 MHz, $CDCl_3$), δ = 9.80 (t, *J* = 1.1 Hz, 1H, CHO), 7.20 – 7.12 (m, 1H, CH_{ar}), 6.82 – 6.74 (m, 2H, 2xCH_{ar}), 2.93 (t, *J* = 7.6 Hz, 2H, CH_2), 2.64 (t, *J* = 7.6 Hz, 2H, CH_2) ppm; ^{13}C NMR (101 MHz, $CDCl_3$), δ = 178.83 (s, CHO), 161.48 (ddd, *J* = 89.6, 77.2, 11.9 Hz, C_{ar}-F), 131.24 (dd, *J* = 9.9, 6.4 Hz, C_{ar}), 122.82 (dd, *J* = 15.8, 3.8 Hz, 2xC_{ar}), 111.20 (dd, *J* = 21.0, 3.8 Hz, C_{ar}), 103.91 (dd, *J* = 26.0, 25.3 Hz, C_{ar}), 34.15 (t, *J* = 1.4 Hz, **C**H_2-C_{ar}), 23.81 (d, *J* = 2.3 Hz, CH_2**C**H_2CHO) ppm; ^{19}F NMR (376 MHz, $CDCl_3$), δ = -112.51 (ddd, *J* = 15.3, 8.2, 7.0 Hz, 1F), -113.99 - -114.08 (m, 1F) ppm.

3-(3,4-difluorophenyl)propanal (**4f**) [27]

Colourless oil, yield 57%; ^{1}H NMR (400 MHz, $CDCl_3$), 9.79 (t, *J* = 1.1 Hz, 1H, CHO), 7.09 – 6.96 (m, 1H, CH_{ar}), 6.90 (dtd, *J* = 10.2, 4.0, 1.8 Hz, 2H, 2xCH_{ar}), 2.90 (t, *J* = 7.5 Hz, 2H, CH_2), 2.65 (t, *J* = 7.6 Hz, 2H, CH_2) ppm; ^{13}C NMR (101 MHz, $CDCl_3$), 178.40 (s, CHO), 149.72 (ddd, *J* = 125.1, 113.1, 12.7 Hz, C_{ar}-F), 137.08 (dd, *J* = 5.6, 4.0 Hz, C_{ar}), 124.25 (ddd, *J* = 6.1, 3.6, 1.2 Hz, 2xC_{ar}), 117.28 (ddd, J = 16.4, 7.6, 0.6 Hz, 2xC_{ar}), 35.33 (d, *J* = 1.0 Hz, **C**H_2-C_{ar}), 29.75 (d, *J* = 1.4 Hz, CH_2**C**H_2CHO) ppm; ^{19}F NMR (376 MHz, $CDCl_3$), δ = -137.69 – -137.85 (m, 1F), -141.15 – -141.27 (m, 1F) ppm.

3-(4-trifluoromethylphenyl)propanal (**4g**) [28]

Colourless oil, yield 74%; ^{1}H NMR (400 MHz, $CDCl_3$), 9.81 (t, *J* = 0.9 Hz, 1H, CHO), 7.42 (dd, *J* = 94.0, 8.0 Hz, 4H, 4xCH_{ar}), 3.00 (t, *J* = 7.4 Hz, 2H, CH_2), 2.81 (t, *J* = 7.3 Hz, 2H, CH_2) ppm; ^{13}C NMR (101 MHz, $CDCl_3$), δ = 178.57 (s, CHO), 140.05 (q, *J* = 1.3 Hz, C_{ar}), 129.43 (s, 2xC_{ar}); 128.85 (q, *J* = 31.7 Hz, CF_3-C_{ar}), 125.78 (q, *J*= 3.2 Hz, 2xC_{ar}), 124.56 (q, *J* = 271.2 Hz, CF_3-C_{ar}); 34.36 (s, **C**H_2-C_{ar}), 29.47 (s, CH_2**C**H_2CHO) ppm; ^{19}F NMR (376 MHz, $CDCl_3$), δ = -62.33 (s, 3F, CF_3) ppm.

3-(2-trifluoromethylphenyl)propanal (**4h**) [29]

Colourless oil, yield 51%; ^{1}H NMR (400 MHz, $CDCl_3$), 9.81 (s, 1H, CHO), 7.63 (d, *J* = 7.8 Hz, 1H, CH_{ar}), 7.48 (t, *J* = 7.5 Hz, 1H, CH_{ar}), 7.33 (dd, *J* = 18.4, 7.7 Hz, 2H, 2xCH_{ar}) 3.14 (t, *J* = 7.8 Hz, 2H, CH_2), 2.70 – 2.66 (m, 2H, CH_2) ppm; ^{13}C NMR (101 MHz, $CDCl_3$), δ = 178.63 (s, CHO), 138.95 (s, C_{ar}), 132.12 (q, *J* = 0.9 Hz, C_{ar}), 130.96 (s, C_{ar}), 128.66 (q, *J* = 27.8 Hz, **C**$_{ar}$-CF_3), 126.69 (q, *J* = 4.8 Hz, C_{ar}), 126.24 (s, C_{ar}), 124.20 (q, *J* = 273.3 Hz, C_{ar}-**C**F_3), 35.55 (s, **C**H_2-C_{ar}), 27.52 (q, *J* = 1.8 Hz, CH_2**C**H_2CHO) ppm; ^{19}F NMR (376 MHz, $CDCl_3$), δ = -59.74 (s, 3F, CF_3) ppm.

2-(2-bromo-4-fluorophenyl)ethanal (**10a**) [30]

Colourless oil, yield 52%; ^{1}H NMR (400 MHz, $CDCl_3$), δ = 9.74 (t, *J* = 1.6 Hz, 1H, CHO), 7.36 (dd, *J* = 8.2, 2.6 Hz, 1H, CH_{ar}), 7.20 (dd, *J* = 8.5, 5.8 Hz, 1H, CH_{ar}), 7.03 (td, *J* = 8.2, 2.6 Hz, 1H, CH_{ar}), 3.84 (d, *J* = 1.5 Hz, 2H, CH_2) ppm; ^{13}C NMR (101 MHz, $CDCl_3$), -; ^{19}F NMR (376 MHz, $CDCl_3$), δ = -112.24 (td, *J* = 8.0, 5.9 Hz, 1F) ppm.

2-(2-bromo-5-fluorophenyl)ethanal (**10b**) [31]

Colourless oil, yield 32%; ^{1}H NMR (400 MHz, $CDCl_3$), δ = 9.75 (t, *J* = 1.5 Hz, 1H, CHO), 7.56 (dd, *J* = 8.8, 5.3 Hz, 1H, CH_{ar}), 6.97 (dd, *J* = 8.8, 3.0 Hz, 1H, CH_{ar}), 7.03 (td, *J* = 8.2, 2.6 Hz, 1H, CH_{ar}), 6.90 (ddd, *J* = 8.6, 8.0, 3.2 Hz, 1H, CH_{ar}), 3.85 (d, *J* = 1.5 Hz, 2H, CH_2) ppm; ^{13}C NMR (101 MHz, $CDCl_3$), δ = 197.37 (s, CHO), 162.01 (d, *J* = 248.0 Hz, C_{ar}-F), 134.66 (d, *J* = 7.8 Hz, C_{ar}), 134.25 (d, *J* = 8.1 Hz, C_{ar}), 119.17 (d, *J* = 3.3 Hz, C_{ar}), 118.85 (d, *J* = 23.1 Hz, C_{ar}), 116.57 (d, *J* = 22.3 Hz, C_{ar}), 50.35 (d, *J* = 1.4 Hz, CH_2-C_{ar}) ppm; ^{19}F NMR (376 MHz, $CDCl_3$), δ = -114.06 - -114.12 (m, 1F) ppm.

Section S4. *The characterization data of the compounds* ***4b, 4c, 10c-10e***.

3-(2-fluorophenyl)propanal (**4b**) [32,33]

Colourless oil, yield 67.5%; ^{1}H NMR (400 MHz, $CDCl_3$), δ = 9.81 (s, 1H, CHO), 7.24 – 7.15 (m, 2H, 2xCH_{ar}), 7.09 – 6.96 (m, 2H, 2xCH_{ar}), 2.97 (t, *J* = 7.4 Hz, 2H, CH_2), 2.77 (t, *J* = 7.3, 2H, CH_2) ppm; ^{13}C NMR (101 MHz, $CDCl_3$), δ = 177.42 (s, CHO), 161.21 (d, *J* = 245.2 Hz, C_{ar}-F), 130.71 (d, *J* = 4.8 Hz, C_{ar}), 128.24 (d, *J* = 8.1 Hz, C_{ar}), 127.16 (d, *J* = 20.4 Hz, C_{ar}), 124.24 (d, *J* = 3.6 Hz, C_{ar}), 115.45 (d, *J* = 21.9 Hz, C_{ar}), 33.92 (d, *J* = 1.6 Hz, **C**H_2-C_{ar}), 24.38 (d, *J* = 2.8 Hz, CH_2**C**H_2CHO) ppm; ^{19}F NMR (376 MHz, $CDCl_3$), δ = -118.30 (s, 1F) ppm.

3-(3-fluorophenyl)propanal (**4c**) [11]

Colourless oil, yield 71%; ^{1}H NMR (400 MHz, $CDCl_3$), δ = 9.81 (t, *J* = 1.2 Hz, 1H, CHO), 7.24 (qd, *J* = 7.7, 6.2 Hz, 1H, CH_{ar}), 6.99 – 6.96 (m, 1H, CH_{ar}), 6.93 – 6.87 (m, 2H, 2xCH_{ar}), 2.95 (t, *J* = 7.7 Hz, 2H, CH_2), 2.68 (t, *J* = 7.7 Hz, 2H, CH_2) ppm; ^{13}C NMR (101 MHz, $CDCl_3$), δ = 178.72 (s, CHO), 162.99 (d, *J* = 245.7 Hz, C_{ar}-F), 142.70 (d, *J* = 7.3 Hz, C_{ar}), 130.10 (d, *J* = 8.4 Hz, C_{ar}), 124.00 (d, *J* = 2.8 Hz, C_{ar}), 115.30 (d, *J* = 21.2 Hz, C_{ar}), 113.41 (d, *J* = 21.0 Hz, C_{ar}), 35.29 (s, **C**H_2-C_{ar}), 30.30 (d, *J* = 1.8 Hz, CH_2**C**H_2CHO) ppm; ^{19}F NMR (376 MHz, $CDCl_3$), δ = -113.21 (td, *J* = 9.5, 6.3 Hz, 1F) ppm.

2-(3-bromo-4-fluorophenyl)ethanal (**10c**) [23]

Colourless oil, yield 61%; ^{1}H NMR (400 MHz, $CDCl_3$), δ = 9.74 (t, *J* = 1.9 Hz, 1H, CHO), 7.42 – 7.38 (m, 1H, CH_{ar}), 7.12 – 7.10 (m, 2H, 2xCH_{ar}), 3.67 (d, *J* = 1.9 Hz, 2H, CH_2) ppm; ^{13}C NMR (101 MHz, $CDCl_3$), δ = 198.22 (s, CHO), 158.62 (d, *J* = 247.6 Hz, C_{ar}-F), 134.64 (s, C_{ar}), 133.89 (s, C_{ar}), 130.24 (d, *J* = 7.3 Hz, C_{ar}), 116.94 (d, *J* = 22.4 Hz, C_{ar}), 109.56 (d, *J* = 21.2 Hz, C_{ar}), 49.21 (s, **C**H_2-C_{ar}) ppm; ^{19}F NMR (376 MHz, $CDCl_3$), δ = -109.08 (dd, *J* = 13.0, 6.5 Hz, 1F) ppm.

2-(4-bromo-2-fluorophenyl)ethanal (**10d**)

Colourless oil, yield 67%; ^{1}H NMR (400 MHz, CDCl$_3$), δ = 9.73 (dd, *J* = 2.9, 1.6 Hz, 1H, CHO), 7.28 (d, *J* = 8.0 Hz, 2H, 2xCH$_{ar}$), 7.06 (t, *J* = 7.5 Hz, CH$_{ar}$), 3.70 (s, 2H, CH$_2$) ppm; ^{13}C NMR (101 MHz, CDCl$_3$), δ = 197.15 (s, CHO), 160.99 (d, *J* = 251.5 Hz, C$_{ar}$-F), 132.75 (d, *J* = 4.7 Hz, C$_{ar}$), 127.90 (s, C$_{ar}$), 121.87 (d, *J* = 9.3 Hz, C$_{ar}$), 119.39 (d, *J* = 25.1 Hz, C$_{ar}$), 118.74 (d, *J* = 16.5 Hz, C$_{ar}$), 43.59 (s, **C**H$_2$-C$_{ar}$) ppm; ^{19}F NMR (376 MHz, CDCl$_3$), δ = -113.97 – -114.02 (m, 1F) ppm.

2-(4-bromo-3-fluorophenyl)ethanal (**10e**) [9]

Colourless oil, yield 50%; ^{1}H NMR (400 MHz, CDCl$_3$), δ = 9.74 (d, *J* = 1.9 Hz, 1H, CHO), 7.52 (dd, *J* = 8.1, 7.2 Hz, 1H, CH$_{ar}$), 6.99 (dd, *J* = 9.1, 2.0 Hz, 1H, CH$_{ar}$), 6.87 (dd, *J* = 8.1, 2.0 Hz, 1H, CH$_{ar}$), 3.68 (d, *J* = 1.9 Hz, CH$_2$) ppm; ^{13}C NMR (101 MHz, CDCl$_3$), δ = 197.86 (s, CHO), 159.29 (d, *J* = 248.53 Hz, C$_{ar}$-F), 134.00 (s, C$_{ar}$), 126.61 (d, *J* = 3.6 Hz, C$_{ar}$), 117.87 (d, *J* = 22.5 Hz, C$_{ar}$), 108.20 (d, *J* = 20.8 Hz, C$_{ar}$), 98.35 (d, *J* = 20.5 Hz, C$_{ar}$), 49.68 (s, **C**H$_2$-C$_{ar}$) ppm; ^{19}F NMR (376 MHz, CDCl$_3$), δ = -106.37 (dd, *J* = 9.1, 7.2 Hz, 1F) ppm.

Section S5. *The characterization data of the compounds* ***6b-6h and 13a-13e.***

Diphenyl 1-{[(N-benzyloxy)carbonyl]amino}-3-(2-fluorophenyl)propylphosphonate (**6b**)

White solid, yield 63%; ^{1}H NMR (400 MHz, CDCl$_3$), δ = 7.37 – 6.96 (m, 19H, CH$_{ar}$), 5.23 (br d, *J* = 10.3 Hz, 1H, NH), 5.13 (d, *J* = 4.6 Hz, 2H, CH$_2$OC, *trans*), 5.13 (d, *J* = 29.0 Hz, 2H, CH$_2$OC, *cis*), 4.57 – 4.46 (m, 1H, CHP, *trans*), 4.39 – 4.26 (m, 1H, CHP, *cis*), 2.97 – 2.89 (m, 1H, CH$_2$), 2.79 – 2.70 (m, 1H, CH$_2$), 2.43 – 2.30 (br m, 1H, CH$_2$), 2.12 – 1.99 (br m, 1H, CH$_2$) ppm; ^{13}C NMR (101 MHz, CDCl$_3$), δ = 161.20 (d, *J* = 245.2 Hz, C$_{ar}$-F), 155.97 (dd, *J* = 5.7, 4.8 Hz, CONH), 150.15 (dd, *J* = 23.5, 9.8 Hz, 2xC$_{ar}$), 136.13 (s, C$_{ar}$), 130.90 (d, *J* = 4.8 Hz, C$_{ar}$), 129.87 (dd, *J* = 10.9, 0.8 Hz, 4xC$_{ar}$), 128.67 (s, 2xC$_{ar}$), 128.40 (s, 2xC$_{ar}$), 128.27 (s, 2xC$_{ar}$), 125.48 (dd, *J* = 15.0, 1.0 Hz, 2xC$_{ar}$), 124.23 (d, *J* = 3.6 Hz, 2xC$_{ar}$), 120.59 (dd, *J* = 22.3, 4.1 Hz, 4xC$_{ar}$), 115.45 (d, *J* = 21.9 Hz, C$_{ar}$), 67.52 (s, **C**H$_2$Ph), 48.22 (dd, *J* = 158.1, 10.8 Hz, **C**HP), 30.49 (d, *J* = 3.4 Hz, **C**H$_2$CH$_2$CHP), 25.68 (dd, *J* = 14.7, 2.4 Hz, CH$_2$**C**H$_2$CHP) ppm; ^{19}F NMR (376 MHz, CDCl$_3$), δ = -118.12 – -118.21 (m, F-H, *cis*), -118.30 – -118.41 (m, F-H, *trans*) ppm; ^{31}P NMR (162 MHz, CDCl$_3$), δ=17.72 (s, 1P, *trans*), 17.38 (s, 1P, *cis*) ppm; HRMS (ESI-MS) *m/z* [MH]$^+$ calculated for $C_{29}H_{27}FNO_5P$: 520.1689, found: 520.1691; [M+Na]$^+$ calculated for $C_{29}H_{27}FNO_5PNa$: 542.1509, found: 524.1150.

Diphenyl 1-{[(N-benzyloxy)carbonyl]amino}-3-(3-fluorophenyl)propylphosphonate (**6c**)

White solid, yield 57%; ^{1}H NMR (400 MHz, CDCl$_3$), δ = 7.38 – 6.82 (m, 19H, CH$_{ar}$), 5.30 (br d, *J* = 9.0 Hz, 1H, NH), 5.13 (d, *J* = 3.3 Hz, 2H, CH$_2$OC, *trans*), 5.13 (d, *J* = 28.2 Hz, 2H, CH$_2$OC, *cis*), 4.59 – 4.43 (m, 1H, CHP, *trans*), 4.38 – 4.26 (m, 1H, CHP, *cis*), 2.91 – 2.79 (m, 1H, CH$_2$), 2.79 – 2.67 (m, 1H, CH$_2$), 2.41 – 2.28 (br m, 1H, CH$_2$), 2.15 – 1.99 (br m, 1H, CH$_2$) ppm; ^{13}C NMR (101 MHz, CDCl$_3$), δ = 163.01 (d, *J* = 245.7 Hz, C$_{ar}$-F), 156.01 (d, *J* = 6.2 Hz, CONH), 150.14 (dd, *J* = 24.0, 9.7 Hz, 2xC$_{ar}$), 136.11 (s, C$_{ar}$), 130.08 (d, *J* = 8.3 Hz, C$_{ar}$), 129.91 (d, *J* = 12.3 Hz, 2xC$_{ar}$), 128.69 (s, 2xC$_{ar}$), 128.45 (s, 2xC$_{ar}$), 128.32 (s, 2xC$_{ar}$), 125.54 (d, *J* = 16.2 Hz, 2xC$_{ar}$), 124.26 (d, *J* = 2.8 Hz, 2xC$_{ar}$), 120.57 (dd, *J* = 20.3, 4.0 Hz, 4xC$_{ar}$), 115.46 (d, *J* = 21.1 Hz, C$_{ar}$), 113.30 (d, *J* = 21.0 Hz, C$_{ar}$), 67.58 (s, **C**H$_2$Ph), 48.13 (d, *J* = 158.2 Hz, **C**HP), 31.88 (s, **C**H$_2$CH$_2$CHP), 31.77 (d, *J* = 4.6 Hz, CH$_2$**C**H$_2$CHP) ppm; ^{19}F NMR (376 MHz, CDCl$_3$), δ = -112.95 – -113.09 (m, F-H, *cis*), -113.18 (td, *J* = 9.2, 6.1 Hz, F-H, *trans*) ppm; ^{31}P NMR (162 MHz, CDCl$_3$), δ=17.70 (s, 1P, *trans*), 17.41 (s, 1P, *cis*) ppm; HRMS (ESI-MS) *m/z* [MH]$^+$ calculated for $C_{29}H_{27}FNO_5P$: 520.1689, found: 520.1741; [M+Na]$^+$ calculated for $C_{29}H_{27}FNO_5PNa$: 542.1509, found: 524.1556.

Diphenyl 1-{[(N-benzyloxy)carbonyl]amino}-3-(4-fluorophenyl)propylphosphonate (**6d**)

White solid, yield 37%; ^{1}H NMR (400 MHz, CDCl$_3$), δ = 7.36 – 6.89 (m, 19H, CH$_{ar}$), 5.30 (br d, *J* = 10.2 Hz, 1H, NH), 5.13 (d, *J* = 2.7 Hz, 2H, CH$_2$OC, *trans*), 5.13 (d, *J* = 27.1 Hz, 2H, CH$_2$OC, *cis*), 4.49 (dtd, *J* = 17.3, 10.6, 3.6 Hz, 1H, CHP, *trans*), 4.29 (dd, *J* = 25.1, 12.6 Hz, 1H, CHP, *cis*), 2.83 (ddd, *J* = 14.3, 9.3, 5.3 Hz, 1H, CH$_2$), 2.75 – 2.64 (br m, 1H, CH$_2$), 2.37 – 2.25 (br m, 1H, CH$_2$), 2.11 – 1.98 (br m, 1H, CH$_2$) ppm; ^{13}C NMR (101 MHz, CDCl$_3$), δ = 161.57 (d, *J* = 244.1 Hz, C$_{ar}$-F), 156.01 (d, *J* = 6.0 Hz, CONH), 150.16 (dd, *J* = 23.9, 9.7 Hz, 2xC$_{ar}$), 136.13 (s, C$_{ar}$), 136.09 (dd, *J* = 3.3, 0.8 Hz, C$_{ar}$), 130.01 (d, *J* =

7.9 Hz, 2xC_{ar}), 129.88 (dd, *J* = 12.2, 0.8 Hz, 4xC_{ar}), 128.68 (s, C_{ar}), 128.44 (s, 2xC_{ar}), 128.30 (s, 2xC_{ar}), 125.50 (dd, *J* = 16.0, 1.0 Hz, 2xC_{ar}), 120.65 (d, *J* = 4.1 Hz, 2xC_{ar}), 120.45 (d, *J* = 4.2 Hz, 2xC_{ar}), 115.40 (d, *J* = 21.2 Hz, 2xC_{ar}), 67.53 (s, **C**H_2Ph), 48.00 (d, *J* = 158.0 Hz, **C**HP), 32.10 (d, *J* = 4.5 Hz, **C**H_2CH_2CHP), 31.19 (d, *J* = 13.9 Hz, CH_2CH_2CHP) ppm; ^{19}F NMR (376 MHz, $CDCl_3$), δ = -116.62 – -116.72 (m, F-H, *cis*), -116.87 (dq, *J* = 8.8, 5.4 Hz, F-H, *trans*) ppm; ^{31}P NMR (162 MHz, $CDCl_3$), δ = 17.79 (s, 1P, *trans*), 17.53 (s, 1P, *cis*) ppm; HRMS (ESI-MS) *m/z* $[MH]^+$ calculated for $C_{29}H_{27}FNO_5P$: 520.1689, found: 520.1741; $[M+Na]^+$ calculated for $C_{29}H_{27}FNO_5PNa$: 542.1509, found: 524.1500.

Diphenyl 1-{[(N-benzyloxy)carbonyl]amino}-3-(2,4-difluorophenyl)propylphosphonate (**6e**)

White solid, yield 37%; ^{1}H NMR (400 MHz, $CDCl_3$), δ = 7.37 – 7.04 (m, 2H, CH_{ar}), 6.80 – 6.72 (m, 16H, CH_{ar}), 5.26 (br d, *J* = 10.4 Hz, 1H, NH), 5.13 (d, *J* = 3.5 Hz, 2H, CH_2OC, *trans*), 5.13 (d, *J* = 27.9 Hz, 2H, CH_2OC, *cis*), 4.54 – 4.42 (m, 1H, CHP, *trans*), 4.29 (dd, *J* = 26.1, 11.6 Hz, 1H, CHP, *cis*), 2.88 (ddd, *J* = 14.3, 9.4, 5.1 Hz, 1H, CH_2), 2.75 – 2.65 (m, 1H, CH_2), 2.38 – 2.26 (br m, 1H, CH_2), 2.10 – 1.96 (br m, 1H, CH_2) ppm; ^{13}C NMR (101 MHz, $CDCl_3$), δ = 161.42 (ddd, *J* = 85.1, 75.3, 11.4 Hz, 2xC_{ar}, C_{ar}-F), 156.00 (d, *J* = 6.1 Hz, CONH), 150.14 (dd, *J* = 25.6, 7.5 Hz, 2xC_{ar}), 136.10 (s, C_{ar}), 131.46 (s, C_{ar}), 129.88 (d, *J* = 10.9 Hz, 4xC_{ar}), 129.15 – 127.91 (m, 5xC_{ar}), 125.51 (d, *J* = 15.2 Hz, 2xC_{ar}), 123.10 (d, *J* = 13.2 Hz, C_{ar}), 120.56 (d, *J* = 21.0 Hz, 4xC_{ar}), 111.21 (d, *J* = 22.0 Hz, C_{ar}), 103.91 (t, *J* = 26.2 Hz, C_{ar}), 67.59 (s, **C**H_2Ph), 48.06 (d, *J* = 158.1 Hz, **C**HP), 30.51 (s, **C**H_2CH_2CHP), 25.10 (d, *J* = 14.5 Hz, CH_2**C**H_2CHP) ppm; ^{19}F NMR (376 MHz, $CDCl_3$), δ = -112.43 – -112.49 (m, F-H, *cis*), -112.64 (dd, *J* = 15.0, 7.3 Hz, F-H, *trans*), -113.76 – -113.87 (m, F-H, *cis*), -113.99 (dd, *J* = 16.8, 8.4 Hz, F-H, *trans*) ppm; ^{31}P NMR (162 MHz, $CDCl_3$), δ = 17.60 (s, 1P, *trans*), 17.24 (s, 1P, *cis*) ppm; HRMS (ESI-MS) *m/z* $[MH]^+$ calculated for $C_{29}H_{26}F_2NO_5P$: 538.1595, found: 538.1605; $[M+Na]^+$ calculated for $C_{29}H_{26}F_2NO_5PNa$: 560.1414, found: 560.1414.

Diphenyl 1-{[(N-benzyloxy)carbonyl]amino}-3-(3,4-difluorophenyl)propylphosphonate (**6f**)

White solid, yield 56%; ^{1}H NMR (400 MHz, $CDCl_3$), δ = 7.38 – 6.82 (m, 18H, CH_{ar}), 5.35 (br d, *J* = 10.2 Hz, 1H, NH), 5.13 (d, *J* = 2.7 Hz, 1H, CH_2OC, *trans*), 5.13 (d, *J* = 27.2 Hz, 1H, CH_2OC, *cis*), 4.48 (dtd, *J* = 17.4, 10.5, 3.3 Hz, 1H, CHP, *trans*), 4.27 (dd, *J* = 21.2, 9.9 Hz, 1H, CHP, *cis*), 2.80 (ddd, *J* = 14.2, 9.3, 5.3 Hz, 1H, CH_2), 2.72 – 2.63 (m, 1H, CH_2), 2.34 – 2.22 (br m, 1H, CH_2), 2.10 – 1.96 (br m, 1H, CH_2) ppm; ^{13}C NMR (101 MHz, $CDCl_3$), δ = 156.02 (d, *J* = 6.1 Hz, **C**ONH), 150.27 (dd, *J* = 11.3, 7.7 Hz, 2xC_{ar}), 149.60 (ddd, *J* = 136.6, 131.7, 12.6 Hz, 2xC_{ar}, C_{ar}-F), 137.38 (t, *J* = 4.7 Hz, C_{ar}), 136.07 (s, C_{ar}), 129.90 (d, *J* = 12.9 Hz, 4xC_{ar}), 128.75 – 128.20 (m, 5xC_{ar}), 125.56 (d, *J* = 16.7 Hz, 2xC_{ar}), 124.46 (dd, *J* = 6.0, 3.5 Hz, C_{ar}), 120.52 (dd, *J* = 19.0, 4.1 Hz, 4xC_{ar}), 117.31 (dd, *J* = 17.0, 8.6 Hz, 2xC_{ar}), 67.59 (s, **C**H_2Ph), 47.87 (d, *J* = 158.3 Hz), 31.83 (d, *J* = 4.2 Hz), 31.19 (d, *J* = 14.3 Hz) ppm; ^{19}F NMR (376 MHz, $CDCl_3$), δ = -137.48 – -137.64 (m, F-H, *trans*), -137.66 – -137.85 (m, F-H, *cis*), -141.07 – -141.22 (m, F-H, *trans*), -141.29 – -141.46 (m, F-H, *cis*) ppm; ^{31}P NMR (162 MHz, $CDCl_3$), δ = 17.58 (s, 1P, *cis*), 17.28 (s, 1P, *trans*) ppm; HRMS (ESI-MS) *m/z* $[MH]^+$ calculated for $C_{29}H_{26}F_2NO_5P$: 538.1595, found: 538.1714; $[M+Na]^+$ calculated for $C_{29}H_{26}F_2NO_5PNa$: 560.1414, found: 560.1416.

Diphenyl 1-{[(N-benzyloxy)carbonyl]amino}-3-(4-trifluoromethylphenyl)propylphosphonate (**6g**)

White solid, yield 40%; ^{1}H NMR (400 MHz, $CDCl_3$), δ = 7.51 (d, *J* = 8.1 Hz, 2H, 2xCH_{ar}), 7.43 – 7.08 (m, 15H, CH_{ar}), 7.06 (d, *J* = 8.5 Hz, 2H, 2xCH_{ar}), 5.73 (d, *J* = 10.2 Hz, 1H, NH), 5.14 (dd, *J* = 5.9 Hz, 2H, CH_2OC, *trans*), 5.14 (d, *J* = 30.4 Hz, 2H, CH_2OC, *cis*), 4.53 (dtd, *J* = 17.5, 10.6, 3.5 Hz, 1H, CHP, *trans*), 4.28 (dd, *J* = 22.6, 10.3 Hz, 1H, CHP, *cis*), 2.89 (ddd, *J* = 14.3, 9.3, 5.3 Hz, 1H, CH_2), 2.83 – 2.73 (m, 1H, CH_2), 2.38 – 2.26 (br m, 1H, CH_2), 2.17 – 2.05 (br m, 1H, CH_2) ppm; ^{13}C NMR (101 MHz, $CDCl_3$), δ = 156.18 (d, *J* = 6.1 Hz, **C**ONH), 150.16 (dd, *J* = 27.5, 9.8 Hz, 2xC_{ar}), 144.67 (s, C_{ar}), 136.20 (s, C_{ar}), 129.89 (d, *J* = 15.6 Hz, 4xC_{ar}), 128.94 (s, 3xC_{ar}), 128.67 (s, 2xC_{ar}), 128.72 (q, *J* = 32.4 Hz, **C**$_{ar}$-CF_3), 128.41 (s, 2xC_{ar}), 128.25 (s, 2xC_{ar}), 125.55 (dd, *J* = 11.5, 7.8 Hz, 2xC_{ar}), 124.37 (q, *J* = 271.8 Hz, **C**F_3-C_{ar}), 120.60 (d, *J* = 4.1 Hz, 2xC_{ar}), 120.43 (d, *J* = 4.2 Hz, 2xC_{ar}), 67.50 (s, **C**H_2Ph), 48.02 (d, *J* = 158.5 Hz, **C**HP), 31.81 (d, *J* = 14.2 Hz, **C**H_2CH_2CHP), 31.58 (d, *J* = 4.5 Hz, CH_2**C**H_2CHP) ppm; ^{19}F NMR (376 MHz, $CDCl_3$), δ = -62.22 (s, F-H, *trans*), -62.24 (s, F-H, *cis*) ppm; ^{31}P NMR (162 MHz, $CDCl_3$), δ = 17.65 (s, 1P, *trans*), 17.34

(s, 1P, *cis*) ppm; HRMS (ESI-MS) *m/z* $[MH]^+$ calculated for $C_{30}H_{27}F_3NO_5P$: 570.1657, found: 570.1650; $[M+Na]^+$ calculated for $C_{30}H_{27}F_3NO_5PNa$: 592.1476, found: 592.1459.

Diphenyl 1-{[(N-benzyloxy)carbonyl]amino}-3-(2-trifluoromethylphenyl)propylphosphonate (**6h**)

White solid, yield 64%; ^{1}H NMR (400 MHz, $CDCl_3$), δ = 7.61 (d, *J* = 7.8 Hz, 1H, CH_{ar}), 7.44 (t, *J* = 7.4 Hz, 1H, CH_{ar}), 7.37 – 7.06 (m, 17H, CH_{ar}), 5.30 (d, *J* = 10.4 Hz, 1H, NH), 5.15 (s, 1H, CH_2OC, *trans),* 5.15 (d, *J* = 25.4 Hz, 1H, CH_2OC, *cis*), 4.57 (dtd, *J* = 17.4, 10.6, 3.4 Hz, 1H, CHP, *trans*), 4.47 – 4.33 (m, 1H, CHP, *cis*), 3.11 – 3.03 (m, 1H, CH_2), 2.94 – 2.83 (m, 1H, CH_2), 2.43 – 2.29 (br m, 1H, CH_2), 2.10 – 1.96 (br m, 1H, CH_2) ppm; ^{13}C NMR (101 MHz, $CDCl_3$), δ =156.11 (d, *J* = 6.2 Hz, **C**ONH), 150.13 (dd, *J* = 21.3, 9.7 Hz, $2xC_{ar}$), 139.38 (s, $2xC_{ar}$), 136.14 (s, $2xC_{ar}$), 132.09 (s, $2xC_{ar}$), 129.88 (d, *J* = 10.5 Hz, $4xC_{ar}$), 128.81 – 128.10 (m, $5xC_{ar}$), 127.34 (q, *J* = 273.8 Hz, **C**F_3-C_{ar}), 126.23 (q, *J* = 5.9 Hz, C_{ar}), 125.51 (d, *J* = 14.2 Hz, $2xC_{ar}$), 120.59 (dd, *J* = 21.4, 4.0 Hz, $4xC_{ar}$), 67.57 (s, **C**H_2Ph), 48.39 (d, *J* = 157.9 Hz, **C**HP), 32.22 (s, **C**H_2CH_2CHP), 29.12 (d, *J* = 12.9 Hz, CH_2**C**H_2CHP) ppm; ^{19}F NMR (376 MHz, $CDCl_3$), δ = -59.46 (s, 3F, CF_3, *trans*), -59.49 (s, 3F, CF_3, *cis*) ppm; ^{31}P NMR (162 MHz, $CDCl_3$), δ = 17.48 (s, 1P, *trans*), 17.10 (s, 1P, *cis*) ppm; HRMS (ESI-MS) *m/z* $[MH]^+$ calculated for $C_{30}H_{27}F_3NO_5P$: 570.1657, found: 570.1656; $[M+Na]^+$ calculated for $C_{30}H_{27}F_3NO_5PNa$: 592.1476, found: 592.1470.

Diphenyl 1-{[(N-benzyloxy)carbonyl]amino}-2-(2-bromo-4-fluorophenyl)ethylphosphonate (**13a**)

White solid, yield 18%; ^{1}H NMR (400 MHz, $CDCl_3$), δ = 7.35 – 7.04 (m, 17H, $17xCH_{ar}$), 6.84 (td, *J* = 8.2, 2.6 Hz, 1H, CH_{ar}), 5.48 (d, *J* = 10.5 Hz, 1H, NH), 4.95 (d, *J* = 12.9 Hz, 2H, CH_2OC, *trans),* 4.95 (d, *J* = 37.5 Hz, 2H, CH_2OC, *cis*), 4.93 - 4.81 (m, 1H, CHP, *trans*), 3.48 (dt, *J* = 14.2, 4.3 Hz, 1H, CH_2), 3.11 (ddd, *J* = 14.2, 11.7, 9.4 Hz, 1H, CH_2) ppm; ^{13}C NMR (101 MHz, $CDCl_3$), δ = 161.51 (d, *J* = 250.3 Hz, C_{ar}-F), 155.68 (d, *J* = 7.3 Hz, **C**ONH), 150.34 (d, *J* = 9.8 Hz, C_{ar}), 150.06 (d, *J* = 9.7 Hz, C_{ar}), 136.17 (s, C_{ar}), 132.41 (d, *J* = 8.4 Hz, C_{ar}), 131.50 (dd, *J* = 16.2, 3.6 Hz, $2xC_{ar}$), 129.99 (d, *J* = 1.0 Hz, $2xC_{ar}$), 129.84 (d, *J* = 0.8 Hz, $2xC_{ar}$), 128.55 (s, C_{ar}), 128.28 (s, C_{ar}), 128.10 (s, C_{ar}), 125.57 (d, *J* = 15.8 Hz, $2xC_{ar}$), 124.91 (d, *J* = 9.6 Hz, $2xC_{ar}$), 120.56 (dd, *J* = 16.1, 4.2 Hz, $2xC_{ar}$), 120.22 (d, *J* = 24.4 Hz, $2xC_{ar}$), 114.69 (d, *J* = 20.8 Hz, $2xC_{ar}$), 67.19 (s, **C**H_2Ph), 48.49 (dd, *J* = 159.4, 1.0 Hz, **C**HP), 35.51 (d, *J* = 6.6 Hz, **C**H_2CHP) ppm; ^{19}F NMR (376 MHz, $CDCl_3$), δ = -112.52 (dd, *J* = 14.1, 7.4 Hz, F-H, *cis*), -112.82 (dd, *J* = 14.1, 7.9 Hz, F-H, *cis*) ppm; ^{31}P NMR (162 MHz, $CDCl_3$), δ = 16.76 (s, 1P, *trans*), 16.30 (s, 1P, *cis*) ppm; HRMS (ESI-MS) *m/z* $[MH]^+$ calculated for $C_{28}H_{24}BrFNO_5P$: 584.0638, found: 584.0640; $[M+Na]^+$ calculated for $C_{28}H_{24}BrFNO_5PNa$: 606.0457, found: 606.0466.

Diphenyl 1-{[(N-benzyloxy)carbonyl]amino}-2-(2-bromo-5-fluorophenyl)ethylphosphonate (**13b**)

White solid, yield 17%; ^{1}H NMR (400 MHz, $CDCl_3$), δ = 7.46 (dd, *J* = 8.8, 5.3 Hz, 1H, CH_{ar}), 7.35 – 7.07 (m, 15H, $15xCH_{ar}$), 7.00 (dd, *J* = 9.0, 3.0 Hz, 1H, CH_{ar}), 6.86 – 6.79 (m, 1H, CH_{ar}), 5.37 (d, *J* = 10.6 Hz, 1H, NH), 5.08 – 4.83 (m, 1H, CHP, *trans*), 4.96 (d, *J* = 3.0 Hz, 2H, CH_2OC, *trans*), 4.96 (d, *J* = 17 Hz, 2H, CH_2OC, *cis),* 4.95 (d, *J* = 37.5 Hz, 2H, CH_2OC, *cis*), 4.93 - 4.81 (m, 1H, CHP, *trans*), 3.51 (dt, *J* = 14.4 4.3 Hz, 1H, CH_2), 3.11 (ddd, *J* = 14.2, 11.7, 9.2 Hz, 1H, CH_2) ppm; ^{13}C NMR (101 MHz, $CDCl_3$), δ = 161.76 (d, *J* = 247.4 Hz, C_{ar}-F), 155.72 (d, *J* = 7.1 Hz, **C**ONH), 150.31 (d, *J* = 9.4 Hz, C_{ar}), 150.03 (d, *J* = 9.7 Hz, C_{ar}), 137.75 (dd, *J* = 15.9, 7.7 Hz, $2xC_{ar}$), 136.17 (s, C_{ar}), 134.11 (d, *J* = 8.0 Hz, C_{ar}), 129.92 (d, *J* = 16.8 Hz, $2xC_{ar}$), 128.60 (d, *J* = 8.5 Hz, $2xC_{ar}$), 128.29 (d, *J* = 8.8 Hz, $2xC_{ar}$), 128.02 (s, C_{ar}), 125.58 (d, *J* = 16.6 Hz, $2xC_{ar}$), 120.54 (dd, *J* = 16.3, 4.2 Hz, $2xC_{ar}$), 119.12 (d, *J* = 3.2 Hz, $2xC_{ar}$), 118.73 (d, *J* = 22.7 Hz, $2xC_{ar}$), 116.09 (d, *J* = 22.6 H, $2xC_{ar}$), 67.23 (s, **C**H_2Ph), 48.31 (d, *J* = 160.2 Hz, **C**HP), 36.40 (d, *J* = 6.7 Hz, **C**H_2CHP) ppm; ^{19}F NMR (376 MHz, $CDCl_3$), δ = -113.99 – -114.15 (m, F-H, *cis*), -114.37 (dd, *J* = 13.8, 8.3 Hz, F-H, *trans*) ppm; ^{31}P NMR (162 MHz, $CDCl_3$), δ = 16.51 (s, 1P, *trans*), 16.06 (s, 1P, *cis*) ppm; HRMS (ESI-MS) *m/z* $[MH]^+$ calculated for $C_{28}H_{24}BrFNO_5P$: 584.0638, found: 584.0635; $[M+Na]^+$ calculated for $C_{28}H_{24}BrFNO_5PNa$: 606.0457, found: 606.0455.

Diphenyl 1-{[(N-benzyloxy)carbonyl]amino}-2-(3-bromo-4-fluorophenyl)ethylphosphonate (**13c**)

White solid, yield 15%; ^{1}H NMR (400 MHz, $CDCl_3$), δ = 7.42 (dd, *J* = 6.5, 2.0 Hz, 1H, CH_{ar}), 7.34 – 7.01 (m, 16H, 16xCH_{ar}), 6.96 (t, *J* = 8.4 Hz, 1H, CH_{ar}), 5.47 (d, *J* = 10.5 Hz, 1H, NH), 5.02 (d, *J* = 13.3 Hz, 2H, CH_2OC, *trans*), 5.02 (d, *J* = 37.8 Hz, 2H, CH_2OC, *cis*), 4.79 – 4.65 (m, 1H, CHP, *trans*), 3.35 – 3.25 (m, 1H, CH_2), 2.96 (dt, *J* = 14.3, 10.1 Hz, 1H, CH_2) ppm; ^{13}C NMR (101 MHz, $CDCl_3$), δ = 158.29 (d, *J* = 246.8 Hz, C_{ar}-F), 155.81 (d, *J* = 7.3 Hz, **C**ONH), 150.19 (d, *J* = 9.8 Hz, C_{ar}), 149.94 (d, *J* = 9.7 Hz, C_{ar}), 136.11 (s, C_{ar}), 134.47 (s, C_{ar}), 133.48 (dd, *J* = 14.4, 3.8 Hz, 2xC_{ar}), 130.02 (d, *J* = 0.9 Hz, 2xC_{ar}), 129.85 (s, C_{ar}), 128.63 (s, 2xC_{ar}), 128.35 (s, C_{ar}), 128.09 (d, *J* = 16.6 Hz, 2xC_{ar}), 125.72 (d, *J* = 1.0 Hz, 2xC_{ar}), 125.54 (s, C_{ar}), 120.55 (dd, *J* = 20.6, 4.2 Hz, 2xC_{ar}), 116.53 (d, *J* = 22.3 Hz, 2xC_{ar}), 108.95 (d, *J* = 20.9 Hz, 2xC_{ar}), 67.37 (s, **C**H_2Ph), 49.32 (d, *J* = 158.5 Hz, **C**HP), 35.02 (d, *J* = 5.7 Hz, **C**H_2CHP) ppm; ^{19}F NMR (376 MHz, $CDCl_3$), δ = -109.37 (s, F-H, *cis*), -109.62 – -109.70 (m, F-H, *trans*) ppm; ^{31}P NMR (162 MHz, $CDCl_3$), δ = 16.84 (s, 1P, *trans*), 16.41 (s, 1P, *cis*) ppm; HRMS (ESI-MS) *m/z* [MH]$^+$ calculated for $C_{28}H_{24}BrFNO_5P$: 584.0638, found: 584.0638; [M+Na]$^+$ calculated for $C_{28}H_{24}BrFNO_5PNa$: 606.0457, found: 606.0457.

Diphenyl 1-{[(N-benzyloxy)carbonyl}amino}-2-(4-bromo-2-fluorophenyl)ethylphosphonate (**13d**)

White solid, yield 21%; ^{1}H NMR (400 MHz, $CDCl_3$), δ = 7.38 – 7.03 (m, 18H, CH_{ar}), 5.37 (d, *J* = 10.4 Hz, 1H, NH), 4.98 (d, *J* = 14.9 Hz, 2H, CH_2OC, *trans*), 4.98 (d, *J* = 39.5 Hz, 2H, CH_2OC, *cis*), 4.82 – 4.69 (m, 1H, CHP, *trans*), 3.38 – 3.29 (m, 1H, CH_2), 3.04 (dt, *J* = 14.0, 10.5 Hz, 1H, CH_2) ppm; ^{13}C NMR (101 MHz, $CDCl_3$), δ = 161.24 (d, *J* = 250.2 Hz, C_{ar}-F), 155.72 (d, *J* = 7.1 Hz, **C**ONH), 150.23 (d, *J* = 9.6 Hz, C_{ar}), 149.97 (d, *J* = 9.7 Hz, C_{ar}), 136.11 (s, C_{ar}), 132.63 (d, *J* = 4.9 Hz, 2xC_{ar}), 130.00 (d, *J* = 1.0 Hz, 2xC_{ar}), 129.85 (d, *J* = 0.8 Hz, C_{ar}), 128.61 (s, 2xC_{ar}), 128.33 (s, C_{ar}), 128.08 (s, 2xC_{ar}), 127.61 (d, *J* = 3.7 Hz, 2xC_{ar}), 125.69 (d, *J* = 1.2 Hz, C_{ar}), 125.52 (d, *J* = 0.9 Hz, 2xC_{ar}), 120.57 (dd, *J* = 21.1, 4.2 Hz, 2xC_{ar}), 119.15 (d, *J* = 25.4 Hz, 2xC_{ar}), 67.31 (s, **C**H_2Ph), 48.50 (d, *J* = 159.5 Hz, **C**HP), 29.37 (d, *J* = 5.8 Hz, **C**H_2CHP) ppm; ^{19}F NMR (376 MHz, $CDCl_3$), δ = -114.32 (t, *J* = 8.4 Hz, F-H, *trans*), -114.43 (t, *J* = 7.8 Hz, F-H, *cis*) ppm; ^{31}P NMR (162 MHz, $CDCl_3$), δ = 16.61 (s, 1P, *trans*), 16.16 (s, 1P, *cis*) ppm; HRMS (ESI-MS) *m/z* [MH]$^+$ calculated for $C_{28}H_{24}BrFNO_5P$: 584.0638, found: 584.0758; [M+Na]$^+$ calculated for $C_{28}H_{24}BrFNO_5PNa$: 606.0457, found: 606.0462.

Diphenyl 1-{[(N-benzyloxy]carbonylamino}-2-(4-bromo-3-fluorophenyl)ethylphosphonate (**13e**)

White solid, yield 20%; ^{1}H NMR (400 MHz, $CDCl_3$), δ = 7.38 (t, *J* = 7.7 Hz, 1H, CH_{ar}), 7.34 – 7.27 (m, 5H, 5xCH_{ar}), 7.24 – 7.10 (m, 7H, CH_{ar}), 7.04 (d, *J* = 8.4 Hz, 2H, 2x CH_{ar}), 7.00 (dd, *J* = 9.3, 1.8 Hz, 1H, CH_{ar}), 6.88 (dd, *J* = 8.2, 1.6 Hz, 1H, CH_{ar}), 5.41 (d, *J* = 10.3 Hz, 1H, NH), 5.02 (d, *J* = 14.1 Hz, 2H, CH_2OC, *trans*), 5.02 (d, *J* = 38.6 Hz, 2H, CH_2OC, *cis*), 4.74 (dtd, *J* = 17.9, 10.4, 4.4 Hz, 1H, CHP, *trans*), 3.36 – 3.26 (m, 1H, CH_2), 3.04 (dt, *J* = 14.4, 10.0 Hz, 1H, CH_2) ppm; ^{13}C NMR (101 MHz, $CDCl_3$), δ = 158.97 (d, *J* = 248.0 Hz, C_{ar}-F), 155.72 (d, *J* = 6.9 Hz, **C**ONH), 150.03 (dd, *J* = 24.2, 9.7 Hz, 2xC_{ar}), 137.79 (dd, J = 14.3, 7.0 Hz, 2xC_{ar}), 136.03 (s, C_{ar}), 133.57 (d, *J* = 0.7 Hz, 2xC_{ar}), 130.03 (d, *J* = 1.0 Hz, 2xC_{ar}), 129.87 (d, *J* = 0.4 Hz, C_{ar}), 128.65 (s, C_{ar}), 128.40 (s, C_{ar}), 128.11 (s, C_{ar}), 125.69 (d, *J* = 1.2 Hz, C_{ar}), 125.52 (d, *J* = 0.9 Hz, 2xC_{ar}), 120.57 (dd, *J* = 21.1, 4.2 Hz, 2xC_{ar}), 119.15 (d, *J* = 25.4 Hz, 2xC_{ar}), 67.31 (s, **C**H_2Ph), 48.50 (d, *J* = 159.5 Hz, **C**HP), 29.37 (d, *J* = 5.8 Hz, **C**H_2CHP) ppm; ^{19}F NMR (376 MHz, $CDCl_3$), δ = -106.68 (t, *J* = 7.7 Hz, F-H, *cis*), -106.92 (t, *J* = 8.2 Hz, F-H, *cis*) ppm; ^{31}P NMR (162 MHz, $CDCl_3$), δ = 16.75 (s, 1P, *trans*), 16.31 (s, 1P, *cis*) ppm; HRMS (ESI-MS) *m/z* [MH]$^+$ calculated for $C_{28}H_{24}BrFNO_5P$: 584.0638, found: 584.0629; [M+Na]$^+$ calculated for $C_{28}H_{24}BrFNO_5PNa$: 606.0457, found: 606.0464.

Section S6. *The characterization data of the compounds* ***14c, 14f, 14h, 16d and 16e.***

Dimethyl 1-{[(N-benzyloxy)carbonyl]amino}-3-(3-fluorophenyl)propylphosphonate (**14c**)

White solid, yield 63%; ^{1}H NMR (400 MHz, $CDCl_3$), δ = 7.37 – 7.27 (m, 5H, 5xCH_{ar}), 7.20 (dd, *J* = 15.1, 7.6 Hz, 1H, CH_{ar}), 6.94 – 6.83 (m, 3H, 3xCH_{ar}), 5.22 (d, *J* = 9.6 Hz, 1H, NH), 5.13 (d, *J* = 3.6 Hz, 2H, CH_2OC, *trans*), 5.13 (d, *J* = 28.0 Hz, 2H, CH_2OC, *cis*), 4.18 – 4.07 (m, 1H, CHP, *trans*), 3.71 (t, *J* = 11.0

Hz, 6H, 2xCH_3), 2.82 – 2.73 (m, 1H, CH_2), 2.69 – 2.60 (m, 1H, CH_2), 2.19 – 2.07 (m, 1H, CH_2), 1.95 – 1.81 (m, 1H, CH_2) ppm; ^{13}C NMR (101 MHz, $CDCl_3$), δ = 162.99 (d, *J* = 245.6 Hz, C_{ar}-F), 156.13 (d, *J* = 5.3 Hz, CONH), 143.31 (d, *J* = 7.4 Hz, C_{ar}), 136.26 (s, C_{ar}), 129.99 (d, *J* = 8.3 Hz, C_{ar}), 128.64 (s, 2xC_{ar}), 128.40 (s, 2xC_{ar}), 128.37 (s, C_{ar}), 124.21 (d, *J* = 2.8 Hz, C_{ar}), 115.39 (d, *J* = 21.0 Hz, C_{ar}), 113.18 (d, *J* = 21.0 Hz, C_{ar}), 67.39 (s, **C**H_2Ph), 53.40 (d, *J* = 7.1 Hz, OCH_3), 53.23 (d, *J* = 6.5 Hz, OCH_3), 46.93 (d, *J* = 156.3 Hz, **C**HP), 31.89 (d, *J* = 12.0 Hz, CH_2**C**H_2CHP), 25.68 (d, *J* = 3.3 Hz, **C**H_2CH_2CHP) ppm; ^{19}F NMR (376 MHz, $CDCl_3$), δ = -113.24 (dd, *J* = 14.0, 8.4 Hz, F-H, *cis*), -113.35 (td, *J* = 9.3, 6.1 Hz, F-H, *trans*) ppm; ^{31}P NMR (162 MHz, $CDCl_3$), δ = 27.45 (s, 1P, *trans*), 26.97 (s, 1P, *cis*) ppm; HRMS (ESI-MS) *m/z* $[MH]^+$ calculated for $C_{19}H_{23}FNO_5P$: 396.1376, found: 396.1380; $[M+Na]^+$ calculated for $C_{19}H_{23}FNO_5PNa$: 418.1196, found: 418.1165.

Dimethyl 1-{[(N-benzyloxy)carbonyl]amino}-3-(3,4-difluorophenyl)propylphosphonate (**14f**)

Colourless oil, yield 65%; ^{1}H NMR (400 MHz, $CDCl_3$), δ = 7.38 – 7.28 (m, 5H, 5xCH_{ar}), 7.02 (dt, *J* = 10.3, 8.4 Hz, 1H, CH_{ar}), 6.95 (ddd, *J* = 11.1, 7.6, 2.0 Hz, 1H, CH_{ar}), 6.87 – 6.82 (m, 1H, CH_{ar}), 5.18 (d, *J* = 10.4 Hz, 1H, NH), 5.12 (d, *J* = 2.8 Hz, 2H, CH_2OC, *trans*), 5.12 (d, *J* = 27.3 Hz, 2H, CH_2OC, *cis*), 4.16 – 4.03 (m, CHP, *trans*), 3.71 (t, *J* = 10.8 Hz, 6H, 2xCH_3), 2.74 (ddd, *J* = 14.5, 9.6, 5.2 Hz, 1H, CH_2), 2.67 – 2.53 (m, 1H, CH_2), 2.15 – 2.04 (m, 1H, CH_2), 1.92 – 1.77 (m, 1H, CH_2) ppm; ^{13}C NMR (101 MHz, $CDCl_3$), δ = 156.13 (d, *J* = 5.3 Hz, CONH), 149.63 (ddd, *J* = 245.9, 124.2, 12.3 Hz, 2xC_{ar}-F), 137.70 – 137.55 (m, C_{ar}), 136.21 (s, C_{ar}), 128.65 (s, 2xC_{ar}), 128.40 (s, 2xC_{ar}), 128.21 (s, 2xC_{ar}), 124.41 (dd, *J* = 6.0, 3.5 Hz, C_{ar}), 117.25 (dd, *J* = 16.9, 9.5 Hz, C_{ar}), 67.42 (s, **C**H_2Ph), 53.40 (d, *J* = 7.1 Hz, OCH_3), 53.20 (d, *J* = 6.6 Hz, OCH_3), 46.67 (dd, *J* = 156.3, 10.5 Hz, **C**HP, *trans/cis*), 31.56 (d, *J* = 2.3 Hz, **C**H_2CH_2CHP), 31.28 (d, *J* = 13.2 Hz, CH_2**C**H_2CHP) ppm; ^{19}F NMR (376 MHz, $CDCl_3$), δ = -137.85 – -138.02 (m, F-H), -141.46 – -141.66 (m, F-H) ppm; ^{31}P NMR (162 MHz, $CDCl_3$), δ = 27.30 (s, 1P, *trans*), 26.82 (s, 1P, *cis*) ppm; HRMS (ESI-MS) *m/z* $[MH]^+$ calculated for $C_{19}H_{22}F_2NO_5P$: 414.1282, found: 414.1290; $[M+Na]^+$ calculated for $C_{19}H_{22}F_2NO_5PNa$: 436.1101, found: 436.1084.

Dimethyl 1-{[(N-benzyloxy)carbonyl]amino}-3-(2-trifluoromethylphenyl)propylphosphonate (**14h**)

Colourless oil, yield 80%; ^{1}H NMR (400 MHz, $CDCl_3$), δ = 7.59 (d, *J* = 7.8 Hz, 1H, CH_{ar}), 7.44 (t, *J* = 7.4 Hz, 1H, CH_{ar}), 7.37 – 7.25 (m, 5H, 5xCH_{ar}), 5.12 (s, 2H, CH_2OC, *trans*), 5.14 (d, *J* = 25.3 Hz, 2H, CH_2OC, *cis*), 4.26 – 4.14 (m, CHP), 3.72 (dd, *J* = 13.0, 10.7 Hz, 6H, 2xCH_3), 3.03 – 2.94 (m, 1H, CH_2), 2.85 – 2.75 (m, 1H, CH_2), 2.21 – 2.09 (m, 1H, CH_2), 1.93 – 1.76 (m, 1H, CH_2) ppm; ^{13}C NMR (101 MHz, $CDCl_3$), δ = 156.20 (d, *J* = 5.3 Hz, CONH), 139.60 (s, C_{ar}), 136.27 (s, C_{ar}), 132.02 (d, *J* = 1.0 Hz, C_{ar}), 131.39 (s, C_{ar}), 128.64 (s, 2xC_{ar}), 128.33 (s, 2xC_{ar}), 128.10 (dd, *J* = 6.0, 3.5 Hz, 2xC_{ar}), 126.44 (s, C_{ar}), 126.16 (q, *J* = 5.7 Hz, C_{ar}), 124.64 (q, *J* = 273.8 Hz, **C**F_3-C_{ar}), 67.41 (s, **C**H_2Ph), 53.32 (dd, *J* = 8.4, 7.1 Hz, 2xOCH_3), 47.24 (d, *J* = 156.3 Hz, **C**HP), 32.05 (d, *J* = 3.3 Hz, **C**H_2CH_2CHP), 29.19 (d, *J* = 13.0 Hz, CH_2**C**H_2CHP) ppm; ^{19}F NMR (376 MHz, $CDCl_3$), δ = -59.53 (s, 3F, CF_3) ppm; ^{31}P NMR (162 MHz, $CDCl_3$), δ = 27.19 (s, 1P, *trans*), 26.66 (s, 1P, *cis*) ppm; HRMS (ESI-MS) *m/z* $[MH]^+$ calculated for $C_{20}H_{23}F_3NO_5P$: 446.1344, found: 446.1340; $[M+Na]^+$ calculated for $C_{20}H_{23}F_3NO_5PNa$: 468.1164, found: 468.1168.

Dimethyl 1-{[(N-benzyloxy)carbonyl]amino}-2-(4-bromo-2-fluorophenyl)ethylphosphonate (**16d**)

Colourless oil, yield 57%; ^{1}H NMR (400 MHz, $CDCl_3$), δ = 7.36 – 7.27 (m, 5H, 5xCH_{ar}), 7.23 – 7.13 (m, 2H, 2xCH_{ar}), 7.07 (t, *J* = 8.0 Hz, 1H, CH_{ar}), 5.08 (d, *J* = 10.1 Hz, 1H, NH), 4.98 (d, *J* = 37.2 Hz, 2H, CH_2OC, *trans*), 4.98 (d, *J* = 12.5 Hz, 2H, CH_2OC, *cis*), 4.45 – 4.30 (m, CHP), 3.75 (dd, *J* = 15.1, 10.6 Hz, 6H, 2xCH_3), 3.14 (dt, *J* = 13.2, 4.3 Hz, 1H, CH_2), 2.87 (dt, *J* = 13.9, 10.6 Hz, 1H, CH_2) ppm; ^{13}C NMR (101 MHz, $CDCl_3$), δ = 161.20 (d, *J* = 250.0 Hz, C_{ar}-F), 155.79 (d, *J* = 5.9 Hz, CONH), 136.19 (s, C_{ar}), 132.48 (d, *J* = 4.9 Hz, C_{ar}), 128.58 (s, 2xC_{ar}), 128.30 (s, 2xC_{ar}), 127.99 (s, 2xC_{ar}), 127.55 (d, *J* = 3.5 Hz, C_{ar}), 122.89 (t, *J* = 15.1 Hz, C_{ar}), 119.08 (d, *J* = 25.5 Hz, C_{ar}), 67.20 (s, **C**H_2Ph), 53.60 (d, *J* = 6.8 Hz, OCH_3), 53.36 (d, *J* = 6.5 Hz, OCH_3), 47.39 (d, *J* = 157.4 Hz, **C**HP), 29.17 (d, *J* = 3.6 Hz, **C**H_2CHP) ppm; ^{19}F NMR (376 MHz, $CDCl_3$), δ = -114.63 (t, *J* = 8.4 Hz, 1F) ppm; ^{31}P NMR (162 MHz, $CDCl_3$), δ = 26.26 (s, 1P, *trans*), 25.70 (s,

1P, *cis*) ppm; HRMS (ESI-MS) *m/z* [MH]$^+$ calculated for $C_{18}H_{20}BrFNO_5P$: 460.0325, found: 460.0314; [M+Na]$^+$ calculated for $C_{18}H_{20}BrFNO_5P$ Na: 482.0144, found: 482.0197.

Dimethyl 1-{[(N-benzyloxy)carbonyl]amino}-2-(4-bromo-3-fluorophenyl)ethylphosphonate (**16e**)

Colourless oil, yield 63.5%; ^{1}H NMR (400 MHz, $CDCl_3$), δ = 7.39 (t, *J* = 7.7 Hz, 1H, CH_{ar}), 7.36 – 7.26 (m, 5H, 5xCH_{ar}), 7.24 – 7.18 (m, 1H, CH_{ar}), 6.93 (ddd, *J* = 9.3, 8.7, 1.4 Hz, 1H, CH_{ar}), 5.26 (d, *J* = 10.1 Hz, 1H, NH), 5.01 (d, *J* = 35.7 Hz, 2H, CH_2OC, *trans*), 5.01 (d, *J* = 11.2 Hz, 2H, CH_2OC, *cis*), 4.42 – 4.30 (m, CHP), 3.72 (dd, *J* = 19.0, 10.6 Hz, 6H, 2xCH_3), 3.18 – 3.09 (m, 1H, CH_2), 2.82 (dt, *J* = 14.4, 9.9 Hz, 1H, CH_2) ppm; ^{13}C NMR (101 MHz, $CDCl_3$), δ = 158.94 (d, *J* = 247.6 Hz, C_{ar}-F), 155.83 (d, *J* = 5.9 Hz, CONH), 138.37 (dd, *J* = 14.2, 6.8 Hz, C_{ar}), 136.15 (s, C_{ar}), 128.61 (s, 2xC_{ar}), 128.34 (s, 2xC_{ar}), 128.00 (s, 2xC_{ar}), 126.17 (d, *J* = 3.4 Hz, C_{ar}), 117.50 (d, *J* = 22.2 Hz, C_{ar}), 107.41 (d, *J* = 20.9 Hz, C_{ar}), 67.27 (s, **C**H_2Ph), 53.54 (d, *J* = 7.3 Hz, OCH_3), 53.26 (d, *J* = 6.6 Hz, OCH_3), 48.00 (d, *J* = 157.2 Hz, **C**HP), 35.32 (d, *J* = 3.3 Hz, **C**H_2CHP) ppm; ^{19}F NMR (376 MHz, $CDCl_3$), δ = -107.17 (dd, *J* = 9.2, 7.4 Hz, 1F) ppm; ^{31}P NMR (162 MHz, $CDCl_3$), δ = 26.38 (s, 1P, *trans*), 25.79 (s, 1P, *cis*) ppm; HRMS (ESI-MS) *m/z* [MH]$^+$ calculated for $C_{18}H_{20}BrFNO_5P$: 460.0325, found: 460.0327.

Figure S1. ^{1}H (**A**), ^{13}C (**B**) and ^{19}F (**C**) NMR spectra for 3-(4-fluorophenyl)propyl-3-(4-fluorophenyl) propionate (**5d**).

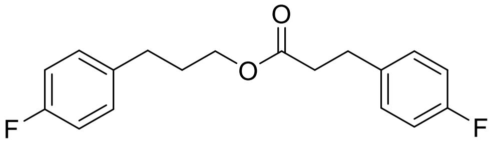

A

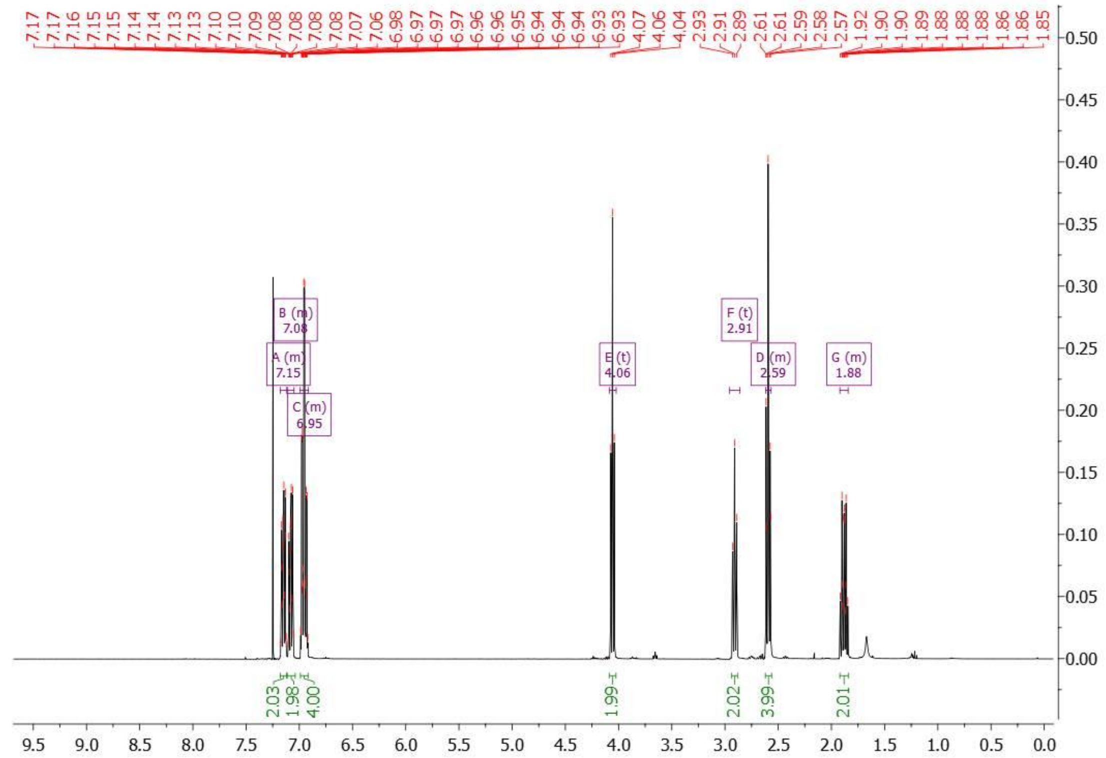

B

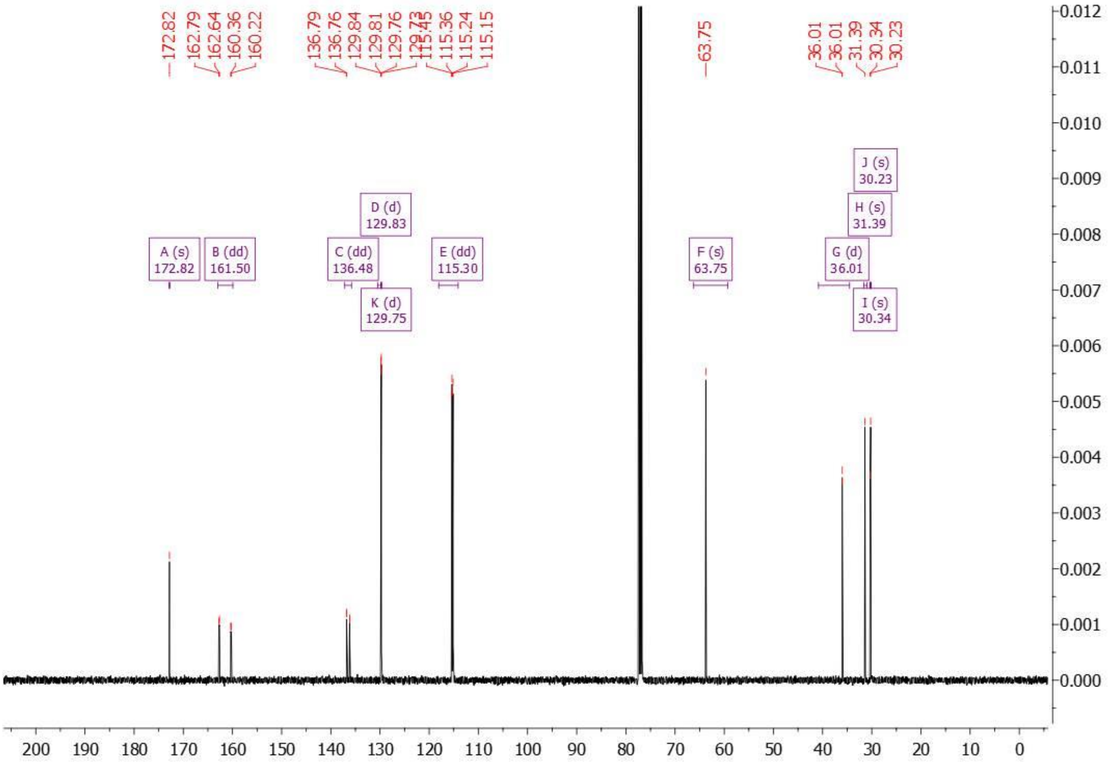

C

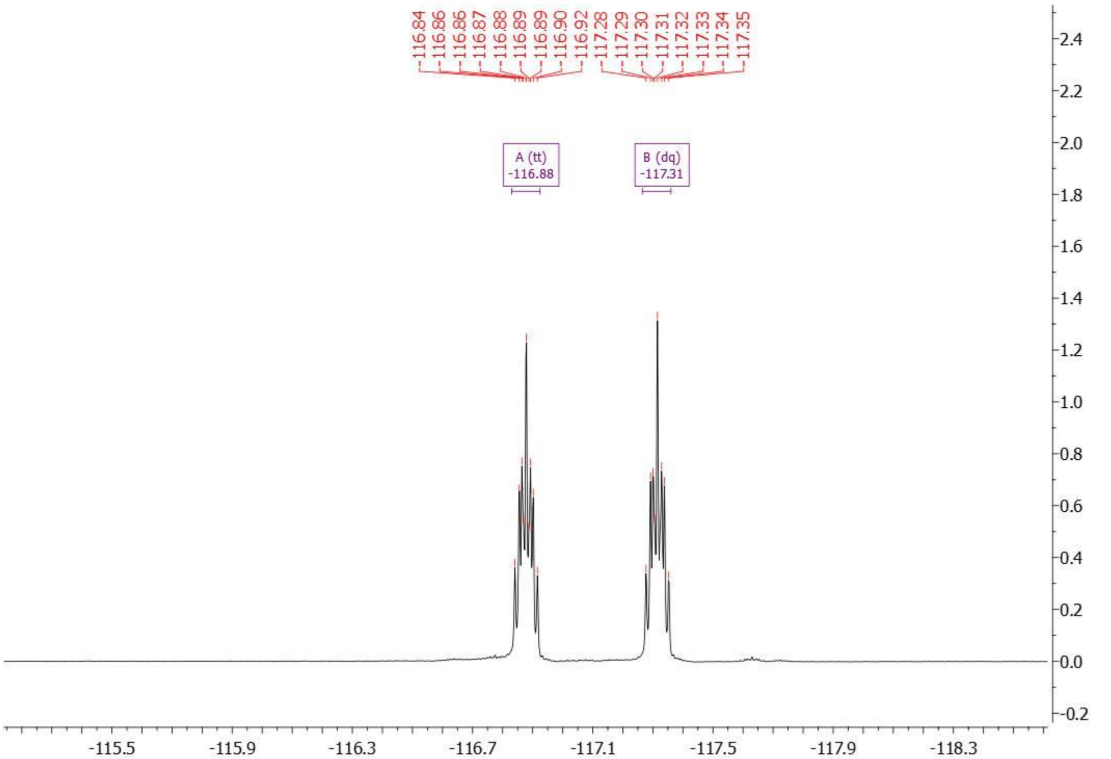

594 **Figure S2.** ^1H (**A**), ^1H-^{31}P HMQC (**B**) and ^1H-^{13}C HMQC (**C**) NMR spectra for
595 1-amino-3-(4-fluorophenyl)propylphosphonic acid (**15d**).

596

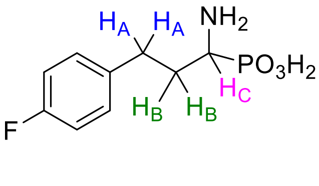

A

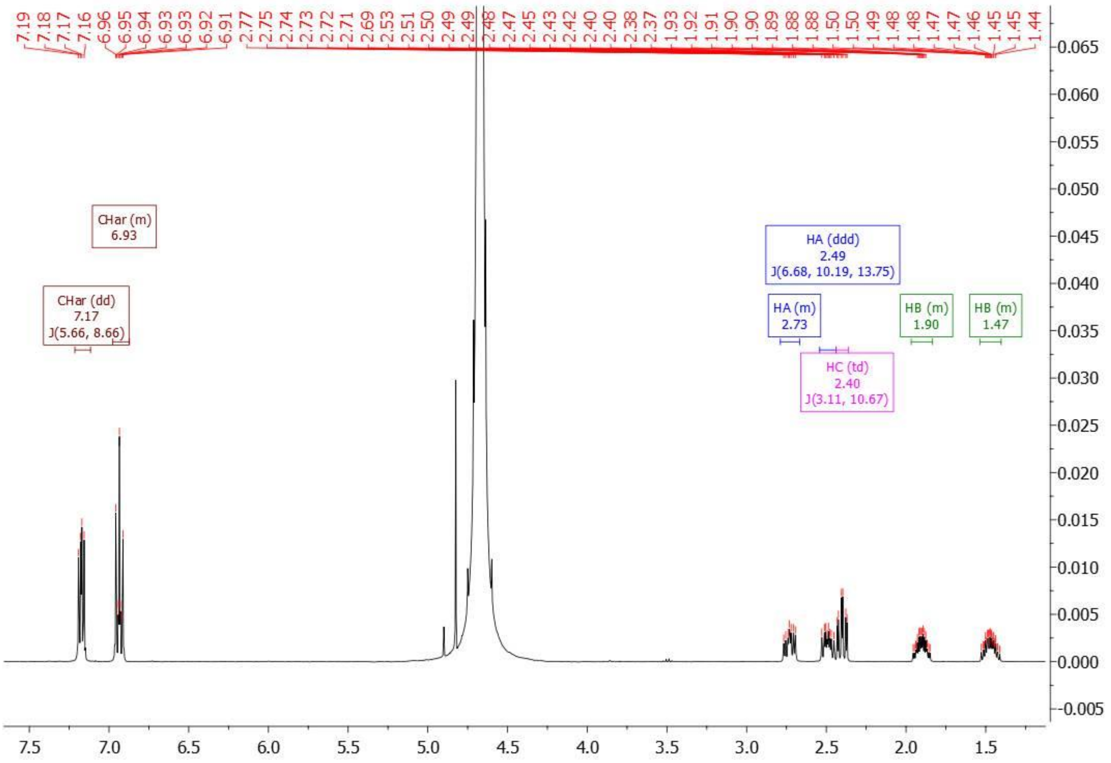
CHar (m)
6.93
CHar (dd)
7.17
J(5.66, 8.66)
HA (ddd)
2.49
J(6.68, 10.19, 13.75)
HA (m)
2.73
HB (m)
1.90
HB (m)
1.47
HC (td)
2.40
J(3.11, 10.67)

B

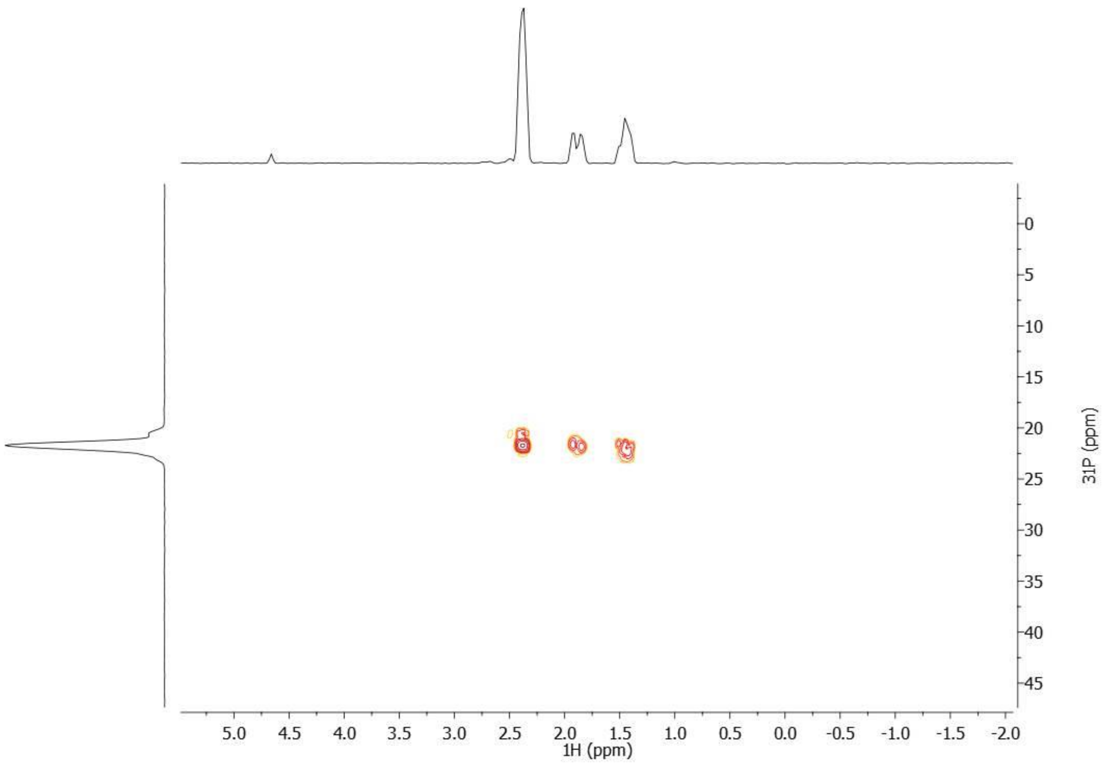
1H (ppm)
31P (ppm)

C

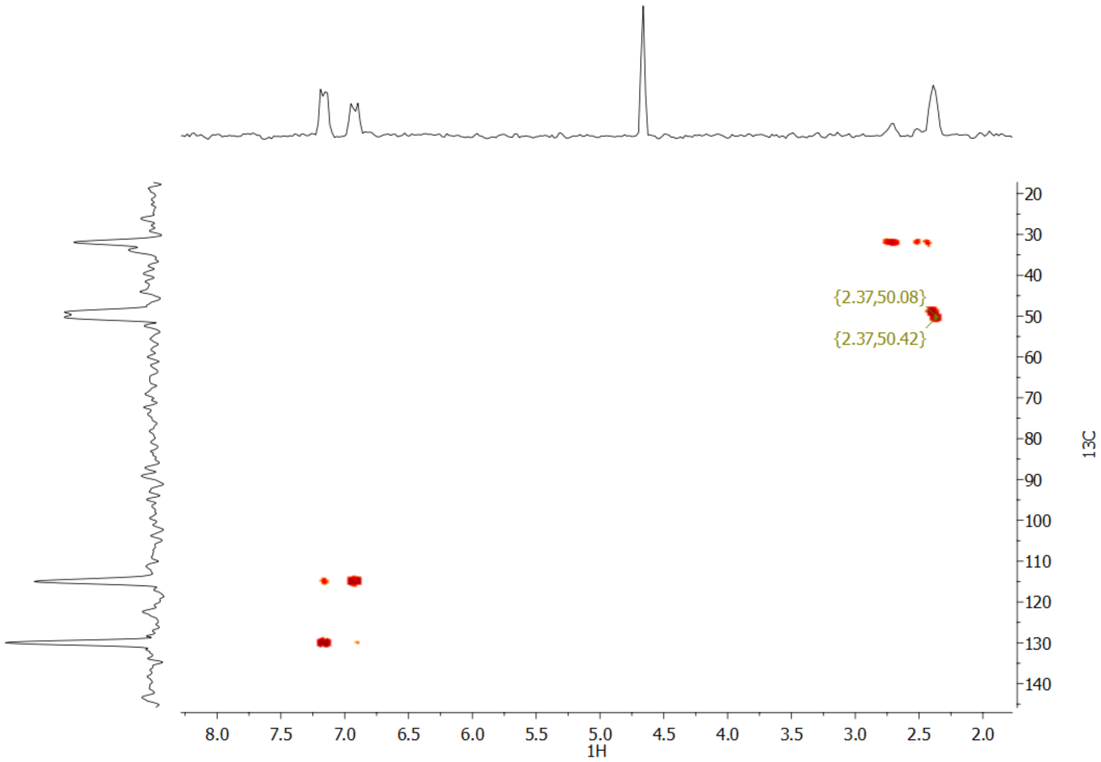

Section S7. *Molecular docking simulations of the inhibitors* ***15c, 15f, 17b*** *and* ***17c*** *binding to active site of pAPN (PDB: 4FKE).*

Figure S7-1. Binding mode of the 1-amino-3-(3-fluorophenyl)propylphosphonic acid (compound **15c**) with the pAPN. The isomer *(S)* is on the left side (**A**), when the *(R)*-isomer is on the right side (**B**). The colouring scheme is identical as in Figure 1.

A **B**

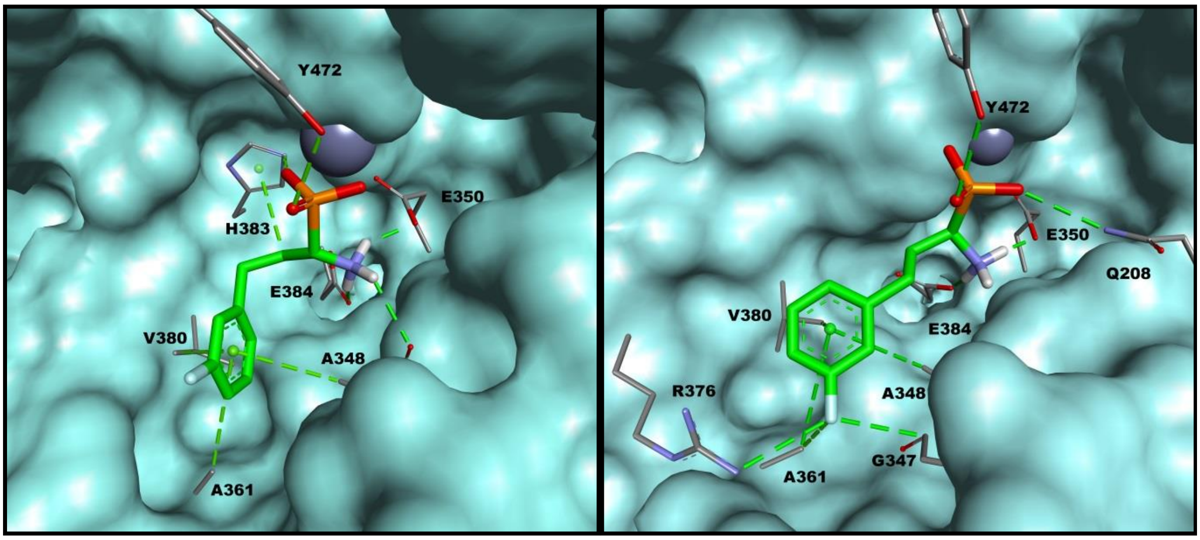

Figure S7-2. Binding mode of the 1-amino-3-(3,4-difluorophenyl)propylphosphonic acid (compound **15f**) with the pAPN. The isomer *(S)* is on the left side (**A**), when the *(R)*-isomer is on the right side (**B**). The colouring scheme is identical as in Figure 1.

A **B**

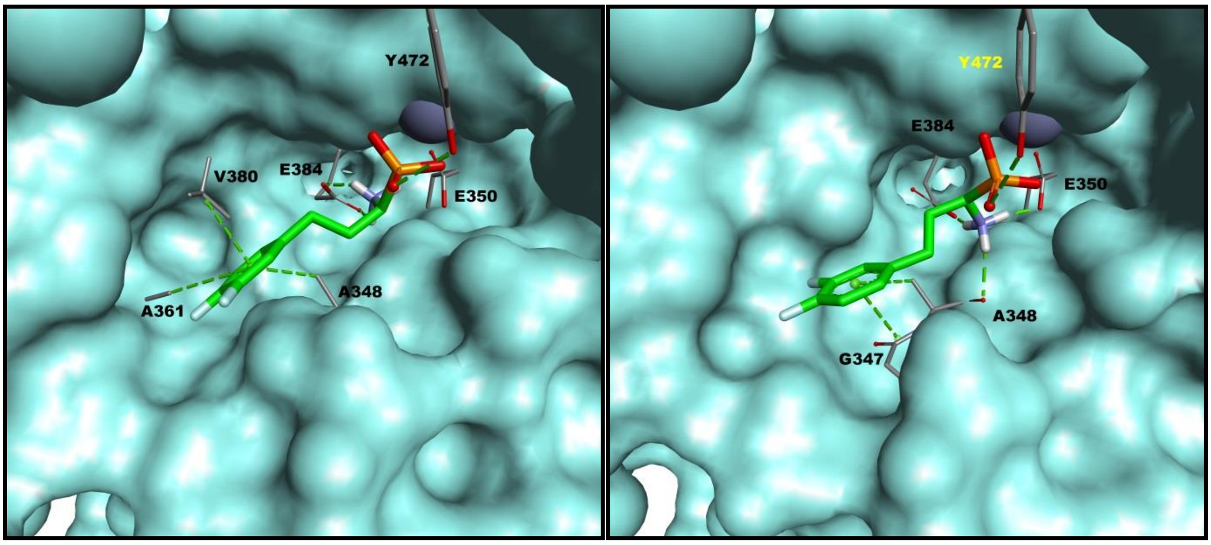

Figure S7-3. Binding mode of the 1-amino-2-(2-bromo-5-fluorophenyl)ethylphosphonic acid (compound **17b**) with the pAPN. The isomer *(S)* is on the left side (**A**), when the *(R)*-isomer is on the right side (**B**). The colouring scheme is identical as in Figure 1. The bromine atom is shown as dark red sphere.

A **B**

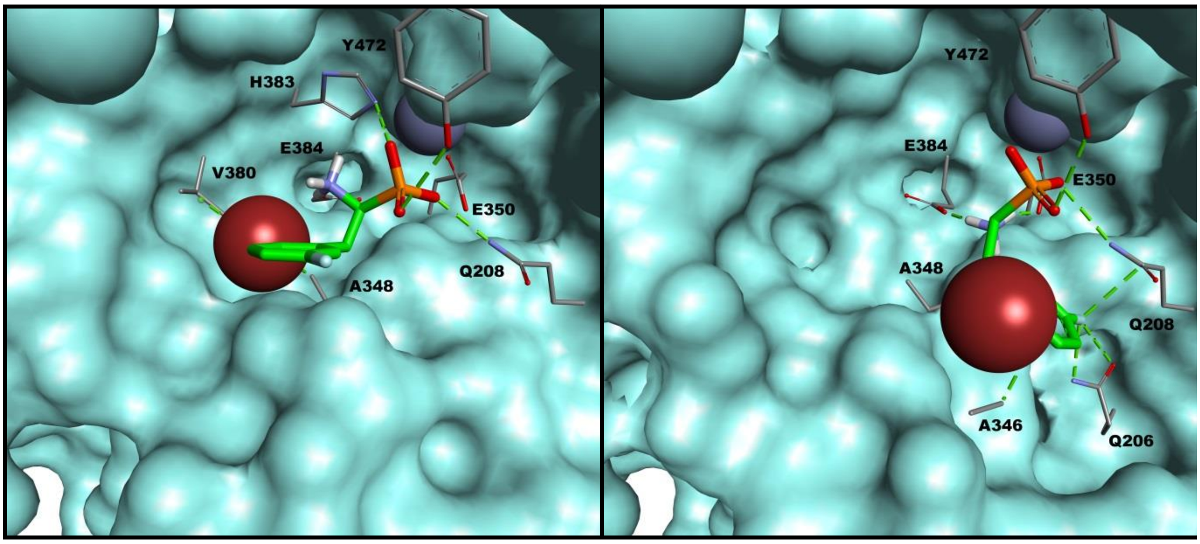

Figure S7-4. Binding mode of the 1-amino-2-(3-bromo-4-fluorophenyl)ethylphosphonic acid (compound **17c**) with the pAPN. The isomer *(S)* is on the left side (**A**), when the *(R)*-isomer is on the right side (**B**). The colouring scheme is identical as in Figure 1. The bromine atom is shown as dark red sphere.

A **B**

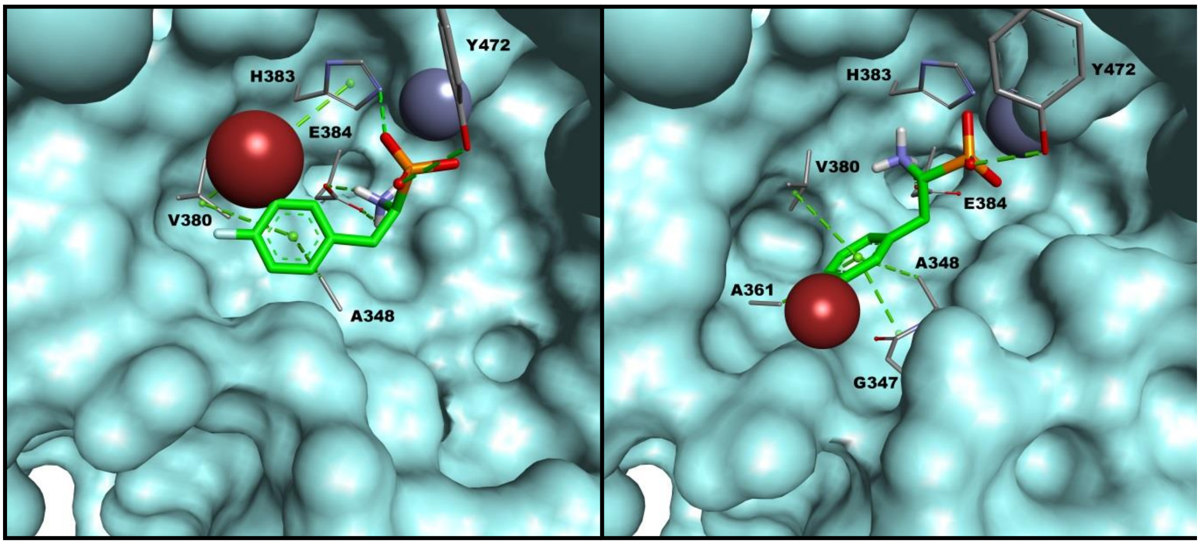

Section S8. Molecular docking simulations of the inhibitors ***15f, 15g*** *and* ***17c*** *binding to active site of hAPN (PDB: 4FYT).*

Figure S8-1. Binding mode of the 1-amino-3-(3,4-difluorophenyl)propylphosphonic acid (compound **15f**) with the hAPN. The isomer *(S)* is on the left side (**A**), when the *(R)*-isomer is on the right side (**B**). The colouring scheme is identical as in Figure 1.

A **B**

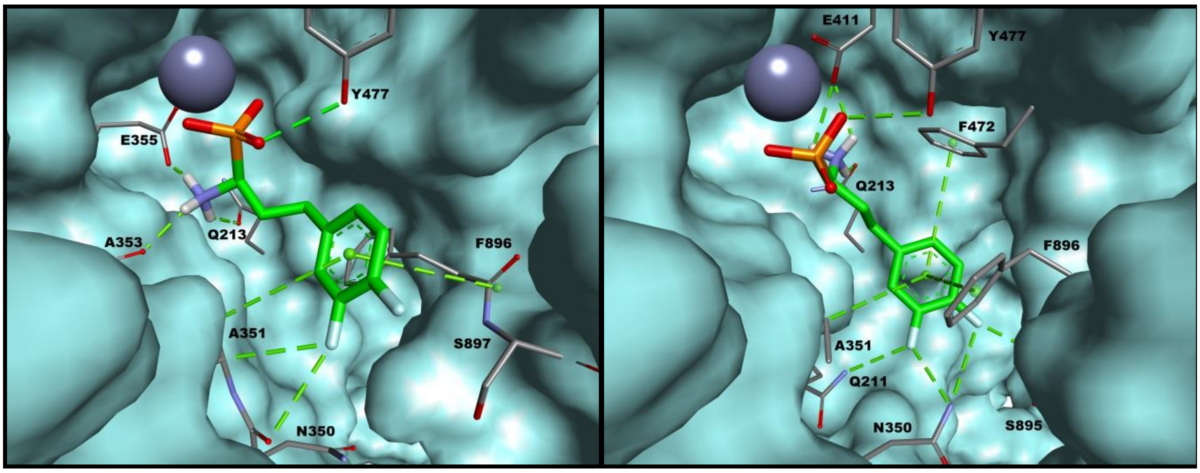

Figure S8-2. Binding mode of the 1-amino-3-(4-trifluoromethylphenyl)propylphosphonic acid (compound **15g**) with the hAPN. The isomer *(S)* is on the left side (**A**), when the *(R)*-isomer is on the right side (**B**). The colouring scheme is identical as in Figure 1.

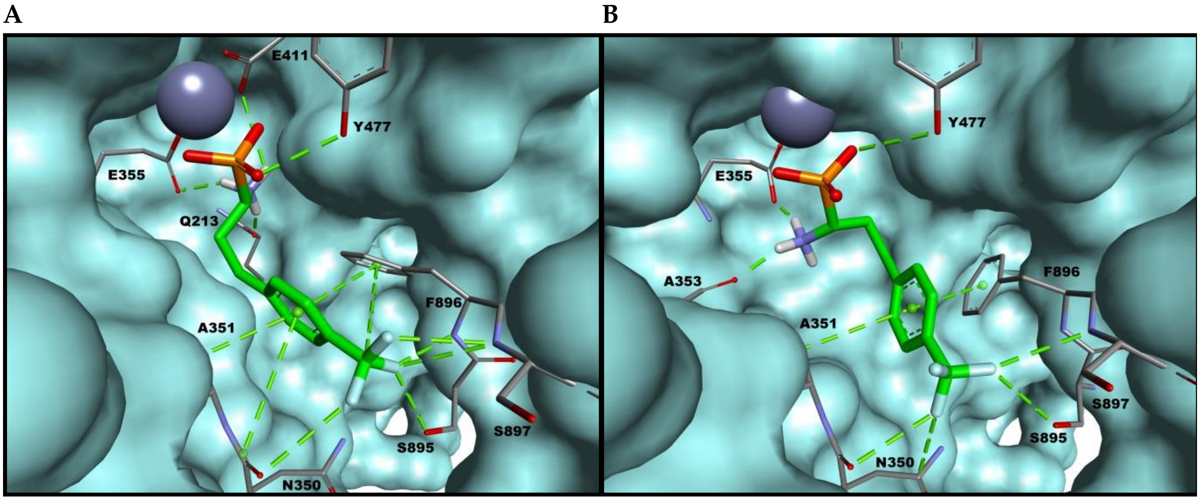

Figure S8-3. Binding mode of the 1-amino-2-(3-bromo-4-fluorophenyl)ethylphosphonic acid (compound **17c**) with the hAPN. The isomer *(S)* is on the left side (**A**), when the *(R)*-isomer is on the right side (**B**). The colouring scheme is identical as in Figure 1. The bromine atom is shown as dark red stick.

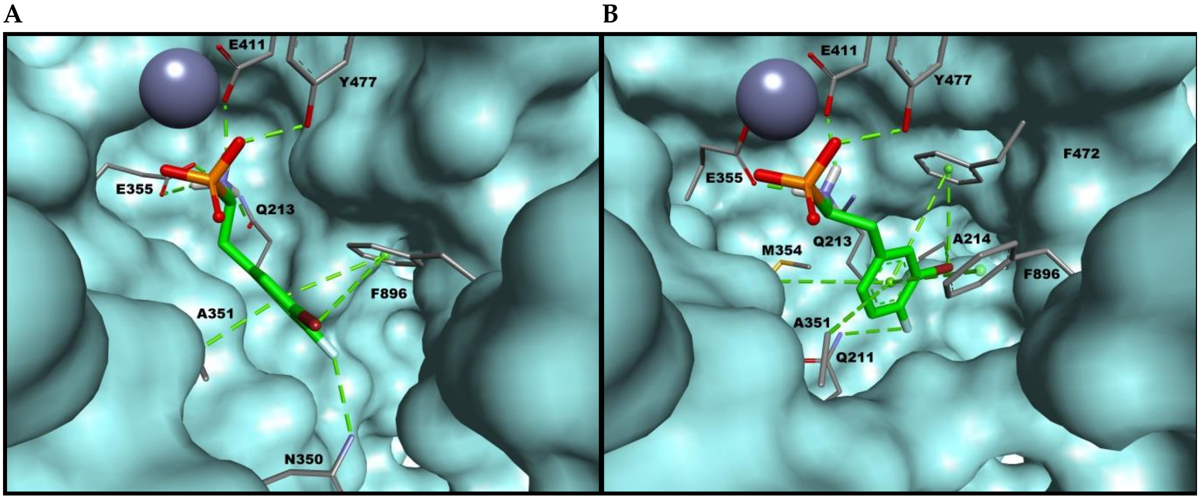

Section S9. *X-Ray analysis of compounds* ***13a, 13c*** *and* ***14c****.*

Figure S9. Molecular structures of diphenyl 1-{[(N-benzyloxy)carbonyl]amino}-2-(2-bromo-4-fluorophenyl)ethylphosphonate (**13a**)(**A**), diphenyl 1-{[(N-benzyloxy)carbonyl]amino}-2-(3-bromo-4-fluorophenyl)ethylphosphonate (**13c**) (**B**) and dimethyl 1-{[(N-benzyloxy)carbonyl]amino}-3-(3-fluorophenyl)propylphosphonate (**14c**) (**C)** in the asymmetric part of unit cell. Displacement ellipsoids are drawn at the 50% probability level.

The geometry around the P atom is distorted tetrahedral, the angles varying from 116.38 (10) ° to 104.49 (9) ° in molecule **13a**, 115.20 (8)° to 102.02 (8)° in **13c** and 114.22 (16)° to 102.41 (14)° in **14c**. All angles involving the non-ester O atom are larger than the others. This corresponds well with other substituted aminophosphonic groups. The arrangement of phenyl groups occurs in molecules **13a** and **13c** (oxygen 04 and O5). In molecule **14c** we can observe the same arrangement of methyl groups. All phenyl rings are planar within experimental error.

A

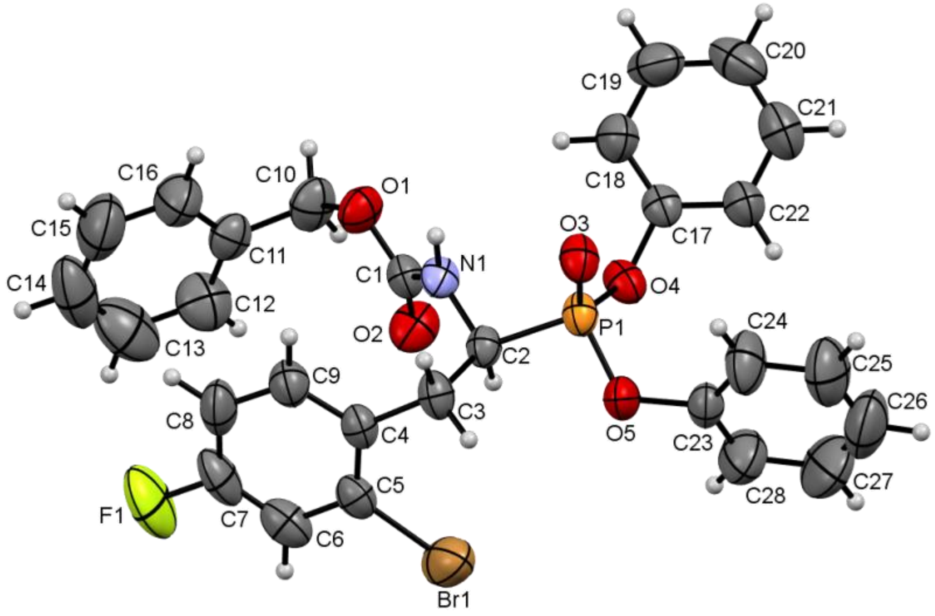

B

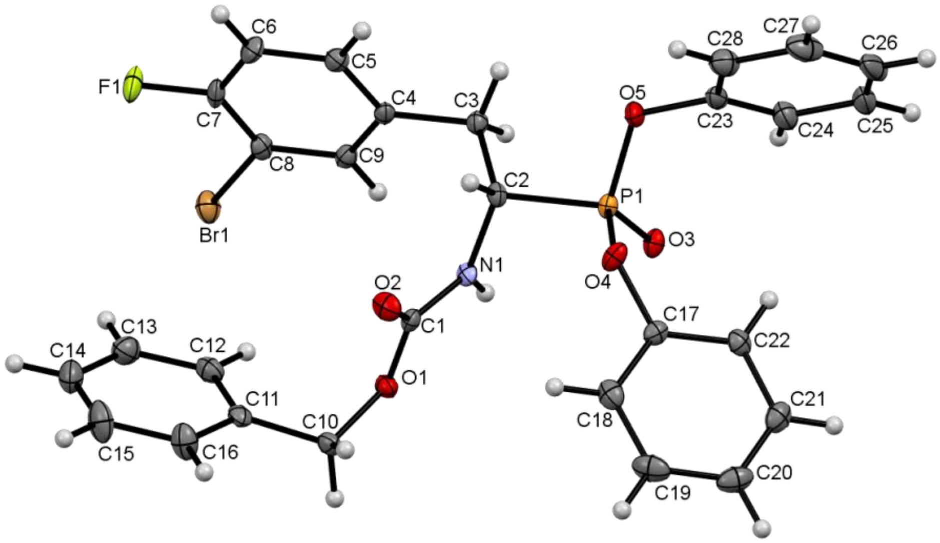

C

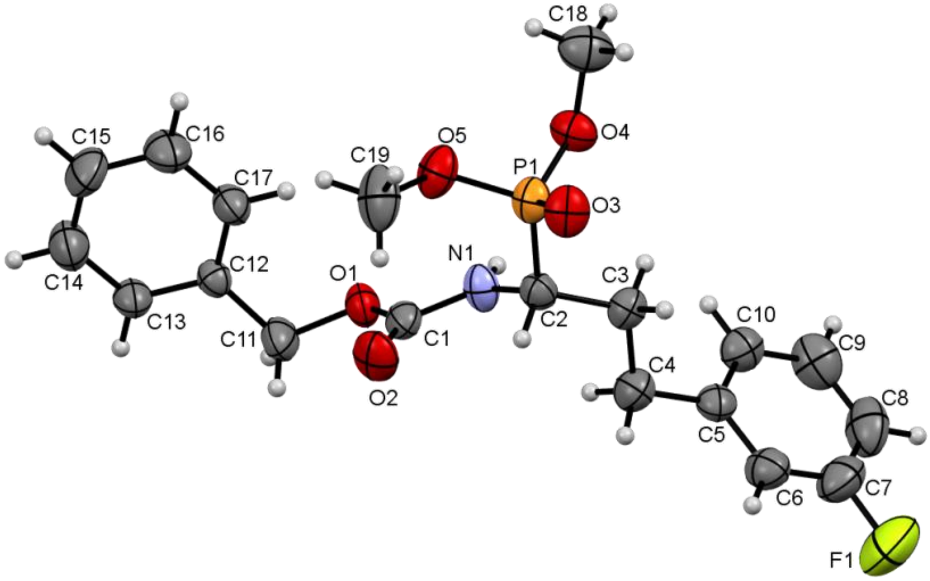

680 **Table S9-1.** Crystal parameters and experimental details of the X-Ray data collection for structure
681 **13a, 13c** and **14c.**

	**13a**	**13c**	**14c**
Crystal data			
Chemical formula	$C_{28}H_{24}BrFNO_5P$	$C_{28}H_{24}BrFNO_5P$	$C_{19}H_{23}FNO_5P$
M_r	584.36	584.36	395.35
Crystal system, space group	Monoclinic, $P2_1/c$	Monoclinic, $P2_1/n$	Monoclinic, $P2_1/c$
Temperature (K)	293	100	293
a, b, c (Å)	13.3129 (7), 10.4517 (4), 19.9282 (12)	8.8213 (2), 15.2255 (3), 19.0081 (4)	11.4935 (12), 18.2514 (16), 10.0442 (11)
β (°)	104.460 (6)	91.843 (2)	111.988 (13)
V (Å^3)	2685.0 (2)	2551.63 (9)	1953.7 (4)
Z	4	4	4
μ (mm^{-1})	1.64	1.72	0.18
Crystal size (mm)	0.5 × 0.3 × 0.1	0.4 × 0.25 × 0.1	0.3 × 0.2 × 0.1
Data collection			
Absorption correction	Multi-scan	Multi-scan	–
T_{min}, T_{max}	0.889, 1.000	0.898, 1.000	
No. of measured, independent and observed [$I > 2\sigma(I)$] reflections	17793, 5237, 2460	17121, 4996, 3862	13191, 3833, 1264
R_{int}	0.035	0.026	0.144
$(\sin \theta/\lambda)_{max}$ (Å^{-1})	0.617	0.617	0.617

Refinement			
$R[F^2 > 2\sigma(F^2)]$, $wR(F^2)$, S	0.034, 0.073, 0.76	0.025, 0.063, 0.95	0.052, 0.071, 0.78
No. of reflections	5237	4996	3833
No. of parameters	334	334	246
$\Delta\rho_{max}$, $\Delta\rho_{min}$ (e Å^{-3})	0.39, −0.49	0.32, −0.33	0.20, −0.23

Table S9-2. Selected geometric parameters for crystal structure **13a** (Å, º).

F1—C7	1.363 (3)	C11—C16	1.367 (3)
P1—O3	1.4574 (15)	C12—C13	1.372 (5)
P1—O4	1.5701 (16)	C12—H12	0.9300
P1—O5	1.5823 (16)	C13—C14	1.354 (5)
P1—C2	1.794 (2)	C13—H13	0.9300
Br1—C5	1.890 (2)	C14—C15	1.362 (5)
N1—C1	1.350 (3)	C14—H14	0.9300
N1—C2	1.440 (3)	C15—C16	1.367 (4)
N1—H1	0.8600	C15—H15	0.9300
O1—C1	1.344 (3)	C16—H16	0.9300
O1—C10	1.451 (3)	C17—C18	1.350 (3)
O2—C1	1.202 (3)	C17—C22	1.364 (3)
O4—C17	1.409 (3)	C18—C19	1.385 (4)
O5—C23	1.410 (3)	C18—H18	0.9300
C2—C3	1.538 (3)	C19—C20	1.368 (4)
C2—H2	0.9800	C19—H19	0.9300
C3—C4	1.498 (3)	C20—C21	1.358 (4)
C3—H3A	0.9700	C20—H20	0.9300
C3—H3B	0.9700	C21—C22	1.371 (3)
C4—C9	1.381 (3)	C21—H21	0.9300
C4—C5	1.387 (3)	C22—H22	0.9300
C5—C6	1.376 (3)	C23—C28	1.345 (3)
C6—C7	1.349 (4)	C23—C24	1.347 (3)
C6—H6	0.9300	C24—C25	1.373 (4)
C7—C8	1.360 (4)	C24—H24	0.9300
C8—C9	1.378 (3)	C25—C26	1.324 (4)
C8—H8	0.9300	C25—H25	0.9300
C9—H9	0.9300	C26—C27	1.361 (4)
C10—C11	1.488 (3)	C26—H26	0.9300

C10—H10A	0.9700	C27—C28	1.421 (4)
C10—H10B	0.9700	C27—H27	0.9300
C11—C12	1.351 (4)	C28—H28	0.9300
O3—P1—O4	114.31 (9)	C16—C11—C10	120.7 (3)
O3—P1—O5	115.56 (9)	C11—C12—C13	120.9 (3)
O4—P1—O5	103.75 (8)	C11—C12—H12	119.5
O3—P1—C2	116.38 (10)	C13—C12—H12	119.5
O4—P1—C2	104.49 (9)	C14—C13—C12	120.5 (4)
O5—P1—C2	100.54 (10)	C14—C13—H13	119.7
C1—N1—C2	120.9 (2)	C12—C13—H13	119.7
C1—N1—H1	119.5	C13—C14—C15	119.5 (4)
C2—N1—H1	119.5	C13—C14—H14	120.3
C1—O1—C10	116.0 (2)	C15—C14—H14	120.3
C17—O4—P1	123.97 (13)	C14—C15—C16	119.3 (4)
C23—O5—P1	121.79 (15)	C14—C15—H15	120.3
O2—C1—O1	125.1 (2)	C16—C15—H15	120.3
O2—C1—N1	125.2 (2)	C11—C16—C15	121.8 (3)
O1—C1—N1	109.7 (2)	C11—C16—H16	119.1
N1—C2—C3	111.73 (18)	C15—C16—H16	119.1
N1—C2—P1	108.55 (15)	C18—C17—C22	122.1 (3)
C3—C2—P1	112.74 (15)	C18—C17—O4	120.3 (2)
N1—C2—H2	107.9	C22—C17—O4	117.5 (2)
C3—C2—H2	107.9	C17—C18—C19	118.6 (3)
P1—C2—H2	107.9	C17—C18—H18	120.7
C4—C3—C2	110.78 (18)	C19—C18—H18	120.7
C4—C3—H3A	109.5	C20—C19—C18	120.2 (3)
C2—C3—H3A	109.5	C20—C19—H19	119.9
C4—C3—H3B	109.5	C18—C19—H19	119.9
C2—C3—H3B	109.5	C21—C20—C19	119.5 (3)
H3A—C3—H3B	108.1	C21—C20—H20	120.2
C9—C4—C5	116.9 (2)	C19—C20—H20	120.2
C9—C4—C3	120.3 (2)	C20—C21—C22	121.0 (3)
C5—C4—C3	122.7 (2)	C20—C21—H21	119.5
C6—C5—C4	122.2 (2)	C22—C21—H21	119.5
C6—C5—Br1	116.8 (2)	C17—C22—C21	118.4 (3)

C4—C5—Br1	120.99 (19)	C17—C22—H22	120.8
C7—C6—C5	117.7 (3)	C21—C22—H22	120.8
C7—C6—H6	121.2	C28—C23—C24	122.5 (3)
C5—C6—H6	121.2	C28—C23—O5	116.4 (3)
C6—C7—C8	123.4 (3)	C24—C23—O5	121.1 (2)
C6—C7—F1	118.7 (3)	C23—C24—C25	119.5 (3)
C8—C7—F1	117.9 (3)	C23—C24—H24	120.3
C7—C8—C9	117.7 (3)	C25—C24—H24	120.3
C7—C8—H8	121.1	C26—C25—C24	120.2 (3)
C9—C8—H8	121.1	C26—C25—H25	119.9
C8—C9—C4	122.0 (3)	C24—C25—H25	119.9
C8—C9—H9	119.0	C25—C26—C27	121.3 (4)
C4—C9—H9	119.0	C25—C26—H26	119.4
O1—C10—C11	111.3 (2)	C27—C26—H26	119.4
O1—C10—H10A	109.4	C26—C27—C28	119.2 (3)
C11—C10—H10A	109.4	C26—C27—H27	120.4
O1—C10—H10B	109.4	C28—C27—H27	120.4
C11—C10—H10B	109.4	C23—C28—C27	117.3 (3)
H10A—C10—H10B	108.0	C23—C28—H28	121.4
C12—C11—C16	118.0 (3)	C27—C28—H28	121.4
C12—C11—C10	121.3 (3)		
O3—P1—O4—C17	0.7 (2)	C5—C4—C9—C8	−1.6 (4)
O5—P1—O4—C17	−125.99 (18)	C3—C4—C9—C8	175.2 (2)
C2—P1—O4—C17	129.07 (19)	C1—O1—C10—C11	92.6 (2)
O3—P1—O5—C23	−71.53 (19)	O1—C10—C11—C12	−116.9 (3)
O4—P1—O5—C23	54.40 (18)	O1—C10—C11—C16	65.1 (3)
C2—P1—O5—C23	162.31 (17)	C16—C11—C12—C13	0.8 (5)
C10—O1—C1—O2	7.5 (3)	C10—C11—C12—C13	−177.3 (3)
C10—O1—C1—N1	−173.87 (18)	C11—C12—C13—C14	0.1 (5)
C2—N1—C1—O2	−8.5 (3)	C12—C13—C14—C15	−1.2 (6)
C2—N1—C1—O1	172.85 (17)	C13—C14—C15—C16	1.4 (6)
C1—N1—C2—C3	−105.3 (2)	C12—C11—C16—C15	−0.6 (5)
C1—N1—C2—P1	129.74 (18)	C10—C11—C16—C15	177.5 (3)
O3—P1—C2—N1	61.02 (18)	C14—C15—C16—C11	−0.5 (5)
O4—P1—C2—N1	−66.05 (16)	P1—O4—C17—C18	−71.9 (3)

O5—P1—C2—N1	−173.37 (14)	P1—O4—C17—C22	109.9 (2)
O3—P1—C2—C3	−63.32 (19)	C22—C17—C18—C19	0.2 (4)
O4—P1—C2—C3	169.61 (15)	O4—C17—C18—C19	−177.9 (2)
O5—P1—C2—C3	62.28 (17)	C17—C18—C19—C20	0.3 (4)
N1—C2—C3—C4	62.0 (2)	C18—C19—C20—C21	−0.1 (5)
P1—C2—C3—C4	−175.41 (17)	C19—C20—C21—C22	−0.7 (5)
C2—C3—C4—C9	−93.8 (3)	C18—C17—C22—C21	−0.9 (4)
C2—C3—C4—C5	82.8 (3)	O4—C17—C22—C21	177.2 (2)
C9—C4—C5—C6	0.2 (3)	C20—C21—C22—C17	1.2 (4)
C3—C4—C5—C6	−176.5 (2)	P1—O5—C23—C28	−123.6 (2)
C9—C4—C5—Br1	−179.15 (17)	P1—O5—C23—C24	56.7 (3)
C3—C4—C5—Br1	4.1 (3)	C28—C23—C24—C25	−0.3 (5)
C4—C5—C6—C7	1.1 (4)	O5—C23—C24—C25	179.4 (3)
Br1—C5—C6—C7	−179.5 (2)	C23—C24—C25—C26	−2.3 (5)
C5—C6—C7—C8	−1.1 (4)	C24—C25—C26—C27	3.8 (6)
C5—C6—C7—F1	179.7 (2)	C25—C26—C27—C28	−2.7 (7)
C6—C7—C8—C9	−0.2 (4)	C24—C23—C28—C27	1.3 (5)
F1—C7—C8—C9	179.0 (2)	O5—C23—C28—C27	−178.4 (3)
C7—C8—C9—C4	1.6 (4)	C26—C27—C28—C23	0.1 (6)

Table S9-3. Selected hydrogen-bond parameters for structure **13a**.

D—H···*A*	*D*—H (Å)	H···*A* (Å)	*D*···*A* (Å)	*D*—H···*A* (°)
N1—H1···O3[i]	0.86	2.14	2.881 (2)	144.6
C2—H2···Br1	0.98	3.09	3.660 (2)	118.3

Symmetry code(s): (i) −*x*+1, −*y*+1, −*z*+1.

Table S9-4. Selected geometric parameters for crystal structure **13c** (Å, º).

F1—C7	1.359 (2)	C11—C16	1.388 (3)
P1—O3	1.4650 (13)	C12—C13	1.379 (3)
P1—O5	1.5815 (14)	C12—H12	0.9300
P1—O4	1.5843 (13)	C13—C14	1.382 (3)
P1—C2	1.8043 (18)	C13—H13	0.9300
Br1—C8	1.8844 (19)	C14—C15	1.379 (3)
N1—C1	1.346 (2)	C14—H14	0.9300
N1—C2	1.451 (2)	C15—C16	1.383 (3)

N1—H1	0.8600	C15—H15	0.9300
O1—C1	1.358 (2)	C16—H16	0.9300
O1—C10	1.446 (2)	C17—C18	1.372 (3)
O2—C1	1.209 (2)	C17—C22	1.383 (3)
O4—C17	1.406 (2)	C18—C19	1.388 (3)
O5—C23	1.419 (2)	C18—H18	0.9300
C2—C3	1.538 (2)	C19—C20	1.382 (3)
C2—H2	0.9800	C19—H19	0.9300
C3—C4	1.512 (2)	C20—C21	1.380 (3)
C3—H3A	0.9700	C20—H20	0.9300
C3—H3B	0.9700	C21—C22	1.384 (3)
C4—C5	1.388 (3)	C21—H21	0.9300
C4—C9	1.390 (3)	C22—H22	0.9300
C5—C6	1.387 (3)	C23—C28	1.378 (3)
C5—H5	0.9300	C23—C24	1.382 (3)
C6—C7	1.369 (3)	C24—C25	1.388 (3)
C6—H6	0.9300	C24—H24	0.9300
C7—C8	1.379 (3)	C25—C26	1.375 (3)
C8—C9	1.385 (3)	C25—H25	0.9300
C9—H9	0.9300	C26—C27	1.375 (3)
C10—C11	1.502 (3)	C26—H26	0.9300
C10—H10A	0.9700	C27—C28	1.390 (3)
C10—H10B	0.9700	C27—H27	0.9300
C11—C12	1.384 (3)	C28—H28	0.9300
O3—P1—O5	115.77 (8)	C16—C11—C10	118.92 (18)
O3—P1—O4	114.45 (7)	C13—C12—C11	120.70 (19)
O5—P1—O4	103.82 (7)	C13—C12—H12	119.7
O3—P1—C2	115.20 (8)	C11—C12—H12	119.7
O5—P1—C2	102.02 (8)	C12—C13—C14	120.2 (2)
O4—P1—C2	103.93 (8)	C12—C13—H13	119.9
C1—N1—C2	121.61 (16)	C14—C13—H13	119.9
C1—N1—H1	119.2	C15—C14—C13	119.5 (2)
C2—N1—H1	119.2	C15—C14—H14	120.2
C1—O1—C10	115.40 (14)	C13—C14—H14	120.2
C17—O4—P1	128.47 (11)	C14—C15—C16	120.3 (2)

C23—O5—P1	120.40 (11)	C14—C15—H15	119.9
O2—C1—N1	125.98 (18)	C16—C15—H15	119.9
O2—C1—O1	124.67 (17)	C15—C16—C11	120.4 (2)
N1—C1—O1	109.33 (16)	C15—C16—H16	119.8
N1—C2—C3	111.67 (15)	C11—C16—H16	119.8
N1—C2—P1	106.64 (12)	C18—C17—C22	121.92 (18)
C3—C2—P1	111.54 (13)	C18—C17—O4	117.18 (17)
N1—C2—H2	109.0	C22—C17—O4	120.78 (17)
C3—C2—H2	109.0	C17—C18—C19	118.88 (19)
P1—C2—H2	109.0	C17—C18—H18	120.6
C4—C3—C2	112.25 (15)	C19—C18—H18	120.6
C4—C3—H3A	109.2	C20—C19—C18	120.3 (2)
C2—C3—H3A	109.2	C20—C19—H19	119.9
C4—C3—H3B	109.2	C18—C19—H19	119.9
C2—C3—H3B	109.2	C21—C20—C19	119.77 (19)
H3A—C3—H3B	107.9	C21—C20—H20	120.1
C5—C4—C9	118.90 (17)	C19—C20—H20	120.1
C5—C4—C3	120.79 (17)	C20—C21—C22	120.72 (19)
C9—C4—C3	120.29 (16)	C20—C21—H21	119.6
C6—C5—C4	120.87 (18)	C22—C21—H21	119.6
C6—C5—H5	119.6	C17—C22—C21	118.41 (19)
C4—C5—H5	119.6	C17—C22—H22	120.8
C7—C6—C5	118.91 (18)	C21—C22—H22	120.8
C7—C6—H6	120.5	C28—C23—C24	122.27 (19)
C5—C6—H6	120.5	C28—C23—O5	117.27 (17)
F1—C7—C6	119.45 (17)	C24—C23—O5	120.46 (18)
F1—C7—C8	118.82 (18)	C23—C24—C25	118.2 (2)
C6—C7—C8	121.73 (17)	C23—C24—H24	120.9
C7—C8—C9	119.06 (18)	C25—C24—H24	120.9
C7—C8—Br1	119.88 (14)	C26—C25—C24	120.4 (2)
C9—C8—Br1	121.05 (14)	C26—C25—H25	119.8
C8—C9—C4	120.52 (17)	C24—C25—H25	119.8
C8—C9—H9	119.7	C25—C26—C27	120.5 (2)
C4—C9—H9	119.7	C25—C26—H26	119.8
O1—C10—C11	112.81 (15)	C27—C26—H26	119.8

O1—C10—H10A	109.0	C26—C27—C28	120.3 (2)
C11—C10—H10A	109.0	C26—C27—H27	119.9
O1—C10—H10B	109.0	C28—C27—H27	119.9
C11—C10—H10B	109.0	C23—C28—C27	118.3 (2)
H10A—C10—H10B	107.8	C23—C28—H28	120.8
C12—C11—C16	118.84 (19)	C27—C28—H28	120.8
C12—C11—C10	122.13 (18)		
O3—P1—O4—C17	−2.54 (18)	C5—C4—C9—C8	−0.5 (3)
O5—P1—O4—C17	−129.67 (15)	C3—C4—C9—C8	−179.04 (17)
C2—P1—O4—C17	123.95 (15)	C1—O1—C10—C11	83.52 (19)
O3—P1—O5—C23	−62.60 (15)	O1—C10—C11—C12	31.2 (3)
O4—P1—O5—C23	63.70 (14)	O1—C10—C11—C16	−152.81 (18)
C2—P1—O5—C23	171.51 (14)	C16—C11—C12—C13	−0.9 (3)
C2—N1—C1—O2	−8.7 (3)	C10—C11—C12—C13	175.08 (18)
C2—N1—C1—O1	172.92 (14)	C11—C12—C13—C14	0.7 (3)
C10—O1—C1—O2	−1.8 (3)	C12—C13—C14—C15	0.2 (3)
C10—O1—C1—N1	176.61 (14)	C13—C14—C15—C16	−0.8 (3)
C1—N1—C2—C3	−120.37 (18)	C14—C15—C16—C11	0.5 (3)
C1—N1—C2—P1	117.55 (16)	C12—C11—C16—C15	0.4 (3)
O3—P1—C2—N1	48.54 (15)	C10—C11—C16—C15	−175.77 (19)
O5—P1—C2—N1	174.79 (12)	P1—O4—C17—C18	−129.37 (16)
O4—P1—C2—N1	−77.48 (13)	P1—O4—C17—C22	54.6 (2)
O3—P1—C2—C3	−73.63 (15)	C22—C17—C18—C19	1.1 (3)
O5—P1—C2—C3	52.63 (14)	O4—C17—C18—C19	−174.98 (17)
O4—P1—C2—C3	160.35 (12)	C17—C18—C19—C20	−1.1 (3)
N1—C2—C3—C4	59.9 (2)	C18—C19—C20—C21	−0.3 (3)
P1—C2—C3—C4	179.11 (13)	C19—C20—C21—C22	1.8 (3)
C2—C3—C4—C5	91.1 (2)	C18—C17—C22—C21	0.4 (3)
C2—C3—C4—C9	−90.3 (2)	O4—C17—C22—C21	176.33 (16)
C9—C4—C5—C6	0.2 (3)	C20—C21—C22—C17	−1.9 (3)
C3—C4—C5—C6	178.81 (18)	P1—O5—C23—C28	−121.49 (17)
C4—C5—C6—C7	−0.1 (3)	P1—O5—C23—C24	59.3 (2)
C5—C6—C7—F1	179.49 (17)	C28—C23—C24—C25	2.6 (3)
C5—C6—C7—C8	0.3 (3)	O5—C23—C24—C25	−178.27 (17)
F1—C7—C8—C9	−179.72 (17)	C23—C24—C25—C26	−0.9 (3)

C6—C7—C8—C9	−0.5 (3)	C24—C25—C26—C27	−0.9 (3)
F1—C7—C8—Br1	−0.8 (2)	C25—C26—C27—C28	1.3 (3)
C6—C7—C8—Br1	178.38 (15)	C24—C23—C28—C27	−2.3 (3)
C7—C8—C9—C4	0.6 (3)	O5—C23—C28—C27	178.57 (17)
Br1—C8—C9—C4	−178.28 (14)	C26—C27—C28—C23	0.3 (3)

687 **Table S9-5.** Selected geometric parameters for crystal structure **14c** (Å, º).

F1—C7	1.365 (4)	C8—C9	1.363 (4)
P1—O3	1.471 (2)	C8—H8	0.9300
P1—O4	1.574 (2)	C9—C10	1.380 (4)
P1—O5	1.579 (2)	C9—H9	0.9300
P1—C2	1.810 (3)	C10—H10	0.9300
N1—C1	1.359 (4)	C11—C12	1.496 (4)
N1—C2	1.436 (3)	C11—H11A	0.9700
N1—H1	0.8600	C11—H11B	0.9700
O1—C1	1.350 (4)	C12—C13	1.381 (4)
O1—C11	1.434 (3)	C12—C17	1.383 (4)
O2—C1	1.198 (4)	C13—C14	1.373 (4)
O4—C18	1.440 (3)	C13—H13	0.9300
O5—C19	1.443 (3)	C14—C15	1.373 (4)
C2—C3	1.533 (4)	C14—H14	0.9300
C2—H2	0.9800	C15—C16	1.377 (4)
C3—C4	1.520 (4)	C15—H15	0.9300
C3—H3A	0.9700	C16—C17	1.381 (4)
C3—H3B	0.9700	C16—H16	0.9300
C4—C5	1.502 (4)	C17—H17	0.9300
C4—H4A	0.9700	C18—H18A	0.9600
C4—H4B	0.9700	C18—H18B	0.9600
C5—C6	1.382 (4)	C18—H18C	0.9600
C5—C10	1.383 (4)	C19—H19A	0.9600
C6—C7	1.377 (5)	C19—H19B	0.9600
C6—H6	0.9300	C19—H19C	0.9600
C7—C8	1.364 (5)		
O3—P1—O4	115.72 (13)	C7—C8—H8	121.4
O3—P1—O5	114.83 (14)	C8—C9—C10	121.2 (4)

O4—P1—O5	101.59 (14)	C8—C9—H9	119.4
O3—P1—C2	114.22 (16)	C10—C9—H9	119.4
O4—P1—C2	102.41 (14)	C9—C10—C5	121.0 (4)
O5—P1—C2	106.52 (15)	C9—C10—H10	119.5
C1—N1—C2	121.0 (3)	C5—C10—H10	119.5
C1—N1—H1	119.5	O1—C11—C12	113.1 (3)
C2—N1—H1	119.5	O1—C11—H11A	109.0
C1—O1—C11	115.4 (3)	C12—C11—H11A	109.0
C18—O4—P1	119.1 (2)	O1—C11—H11B	109.0
C19—O5—P1	121.7 (2)	C12—C11—H11B	109.0
O2—C1—O1	125.7 (4)	H11A—C11—H11B	107.8
O2—C1—N1	125.9 (4)	C13—C12—C17	119.3 (3)
O1—C1—N1	108.5 (3)	C13—C12—C11	117.6 (3)
N1—C2—C3	110.8 (3)	C17—C12—C11	123.1 (3)
N1—C2—P1	112.4 (2)	C14—C13—C12	120.7 (4)
C3—C2—P1	113.0 (2)	C14—C13—H13	119.7
N1—C2—H2	106.8	C12—C13—H13	119.7
C3—C2—H2	106.8	C13—C14—C15	119.9 (4)
P1—C2—H2	106.8	C13—C14—H14	120.1
C4—C3—C2	112.6 (3)	C15—C14—H14	120.1
C4—C3—H3A	109.1	C14—C15—C16	120.1 (4)
C2—C3—H3A	109.1	C14—C15—H15	119.9
C4—C3—H3B	109.1	C16—C15—H15	119.9
C2—C3—H3B	109.1	C15—C16—C17	120.0 (4)
H3A—C3—H3B	107.8	C15—C16—H16	120.0
C5—C4—C3	114.3 (3)	C17—C16—H16	120.0
C5—C4—H4A	108.7	C16—C17—C12	120.0 (4)
C3—C4—H4A	108.7	C16—C17—H17	120.0
C5—C4—H4B	108.7	C12—C17—H17	120.0
C3—C4—H4B	108.7	O4—C18—H18A	109.5
H4A—C4—H4B	107.6	O4—C18—H18B	109.5
C6—C5—C10	118.4 (4)	H18A—C18—H18B	109.5
C6—C5—C4	121.0 (4)	O4—C18—H18C	109.5
C10—C5—C4	120.6 (4)	H18A—C18—H18C	109.5
C7—C6—C5	118.6 (4)	H18B—C18—H18C	109.5

C7—C6—H6	120.7	O5—C19—H19A	109.5
C5—C6—H6	120.7	O5—C19—H19B	109.5
C8—C7—F1	118.9 (5)	H19A—C19—H19B	109.5
C8—C7—C6	123.7 (4)	O5—C19—H19C	109.5
F1—C7—C6	117.4 (5)	H19A—C19—H19C	109.5
C9—C8—C7	117.1 (4)	H19B—C19—H19C	109.5
C9—C8—H8	121.4		
O3—P1—O4—C18	−50.3 (3)	C3—C4—C5—C10	−62.7 (4)
O5—P1—O4—C18	74.8 (3)	C10—C5—C6—C7	−1.0 (6)
C2—P1—O4—C18	−175.2 (2)	C4—C5—C6—C7	178.0 (3)
O3—P1—O5—C19	−33.4 (3)	C5—C6—C7—C8	0.9 (6)
O4—P1—O5—C19	−159.1 (2)	C5—C6—C7—F1	−178.9 (3)
C2—P1—O5—C19	94.1 (3)	F1—C7—C8—C9	179.6 (4)
C11—O1—C1—O2	−3.0 (5)	C6—C7—C8—C9	−0.2 (7)
C11—O1—C1—N1	177.1 (2)	C7—C8—C9—C10	−0.5 (7)
C2—N1—C1—O2	−2.9 (6)	C8—C9—C10—C5	0.5 (6)
C2—N1—C1—O1	177.1 (3)	C6—C5—C10—C9	0.3 (6)
C1—N1—C2—C3	141.8 (3)	C4—C5—C10—C9	−178.7 (3)
C1—N1—C2—P1	−90.8 (3)	C1—O1—C11—C12	94.1 (3)
O3—P1—C2—N1	161.2 (2)	O1—C11—C12—C13	167.0 (3)
O4—P1—C2—N1	−72.9 (2)	O1—C11—C12—C17	−13.3 (5)
O5—P1—C2—N1	33.3 (3)	C17—C12—C13—C14	−0.1 (6)
O3—P1—C2—C3	−72.6 (3)	C11—C12—C13—C14	179.6 (3)
O4—P1—C2—C3	53.3 (3)	C12—C13—C14—C15	0.8 (6)
O5—P1—C2—C3	159.5 (2)	C13—C14—C15—C16	−0.7 (6)
N1—C2—C3—C4	−59.1 (4)	C14—C15—C16—C17	0.0 (6)
P1—C2—C3—C4	173.9 (2)	C15—C16—C17—C12	0.7 (6)
C2—C3—C4—C5	170.6 (3)	C13—C12—C17—C16	−0.6 (6)
C3—C4—C5—C6	118.3 (4)	C11—C12—C17—C16	179.6 (3)

688 **Table S9-6.** Selected hydrogen-bond parameters for structure **14c**.

D—H···*A*	*D*—H (Å)	H···*A* (Å)	*D*···*A* (Å)	*D*—H···*A* (°)
N1—H1···O3^{i}	0.86	2.07	2.916 (3)	167.4
C11—H11B···O1^{ii}	0.97	2.49	3.298 (4)	140.4
C18—H18C···O2^{i}	0.96	2.64	3.243 (4)	121.6

C19—H19B···O1^{iii}	0.96	2.53	3.474 (4)	166.3

689 Symmetry code(s): (i) $x, -y+1/2, z-1/2$; (ii) $-x+1, -y+1, -z+1$; (iii) $x, -y+1/2, z+1/2$.

690 ***Section S10.*** *Characterization of the Final Compounds* ***15a-15h*** *and* ***17a-17e*** *by* ^{1}*H,* ^{13}*C,* ^{19}*F,* ^{31}*P NMR.*

691 **Figure S10-1.** ^{1}H (**A**), ^{31}P (**B**) NMR spectra for compound **15a**.

A

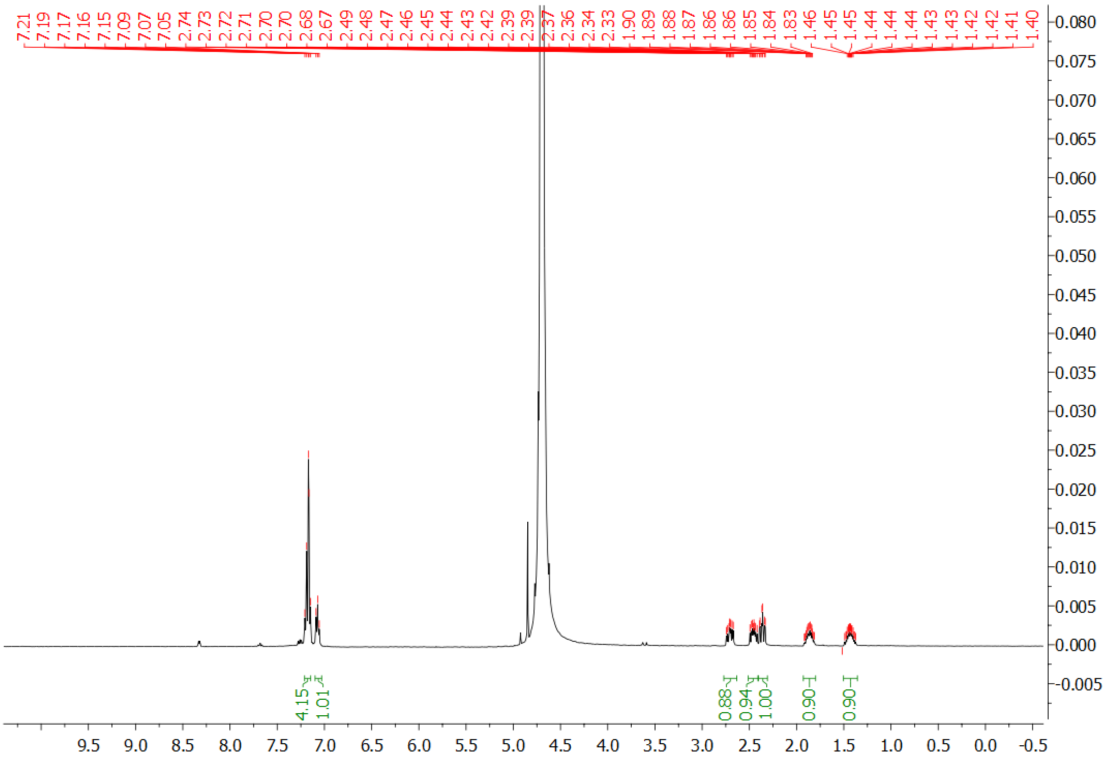

B

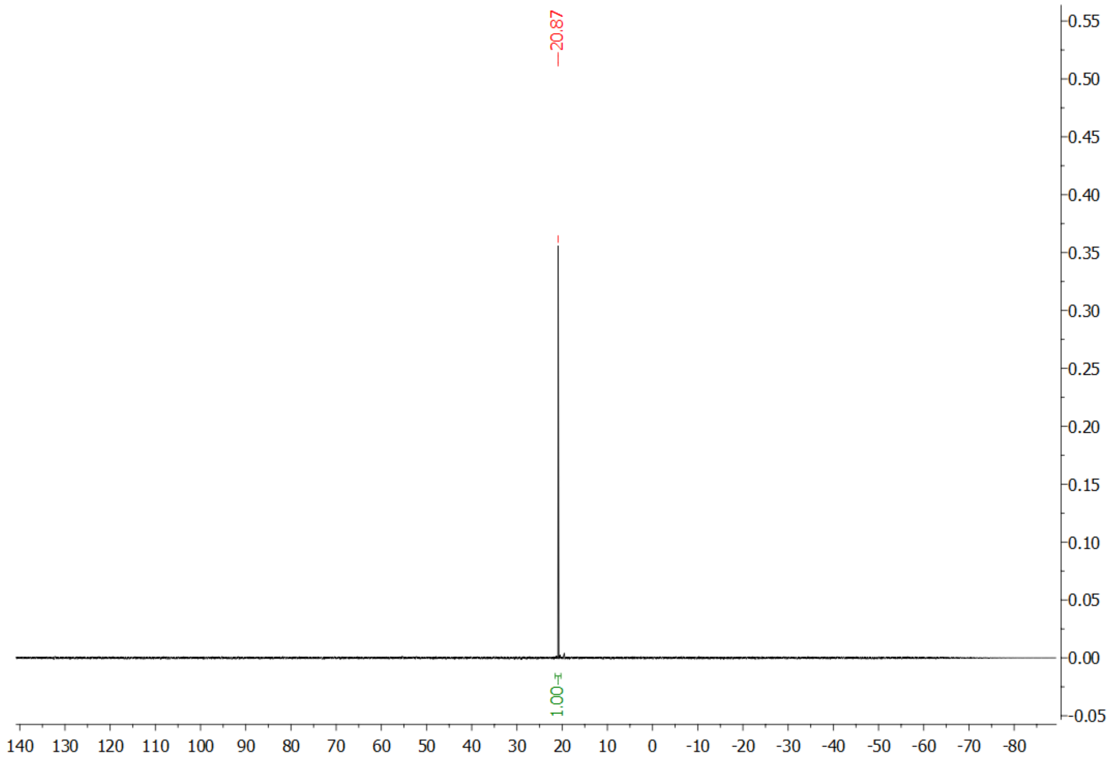

692 **Figure S10-2.** ^{1}H (**A**),^{13}C (**B**), ^{19}F (**C**), ^{31}P (**D**) NMR spectra for compound **15b**.

A

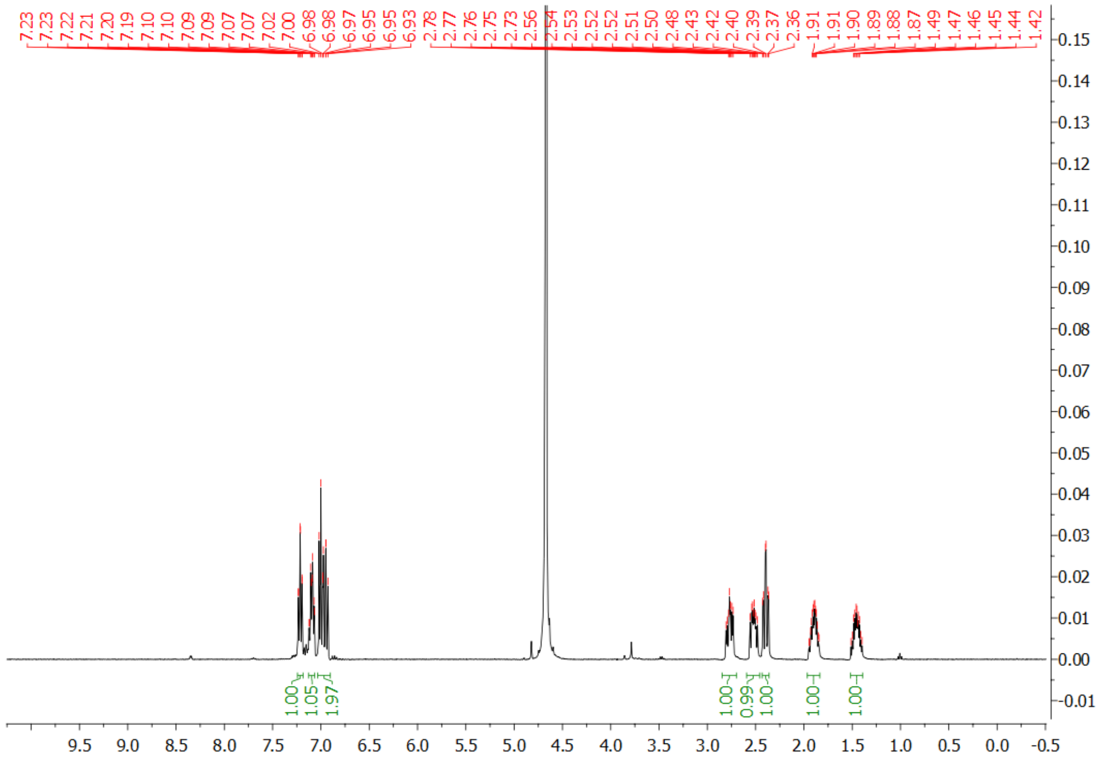

B

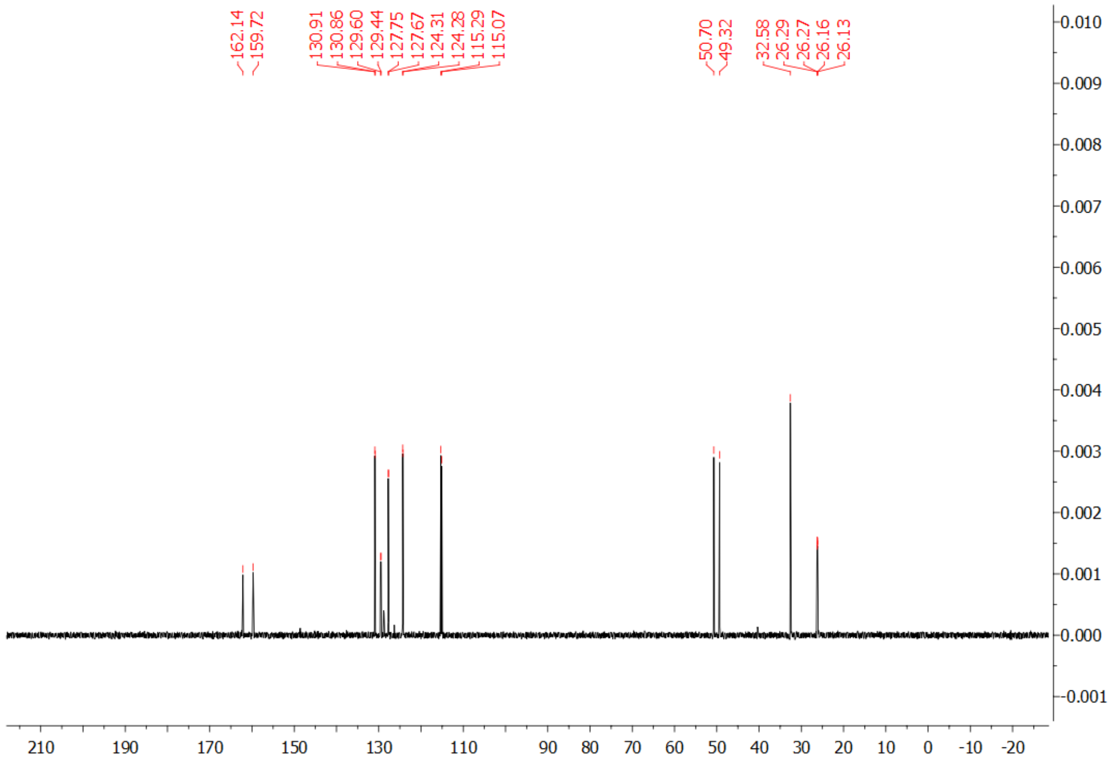
162.14
159.72
130.91
130.86
129.60
129.44
127.75
127.67
124.31
124.28
115.29
115.07
50.70
49.32
32.58
26.29
26.27
26.16
26.13

C

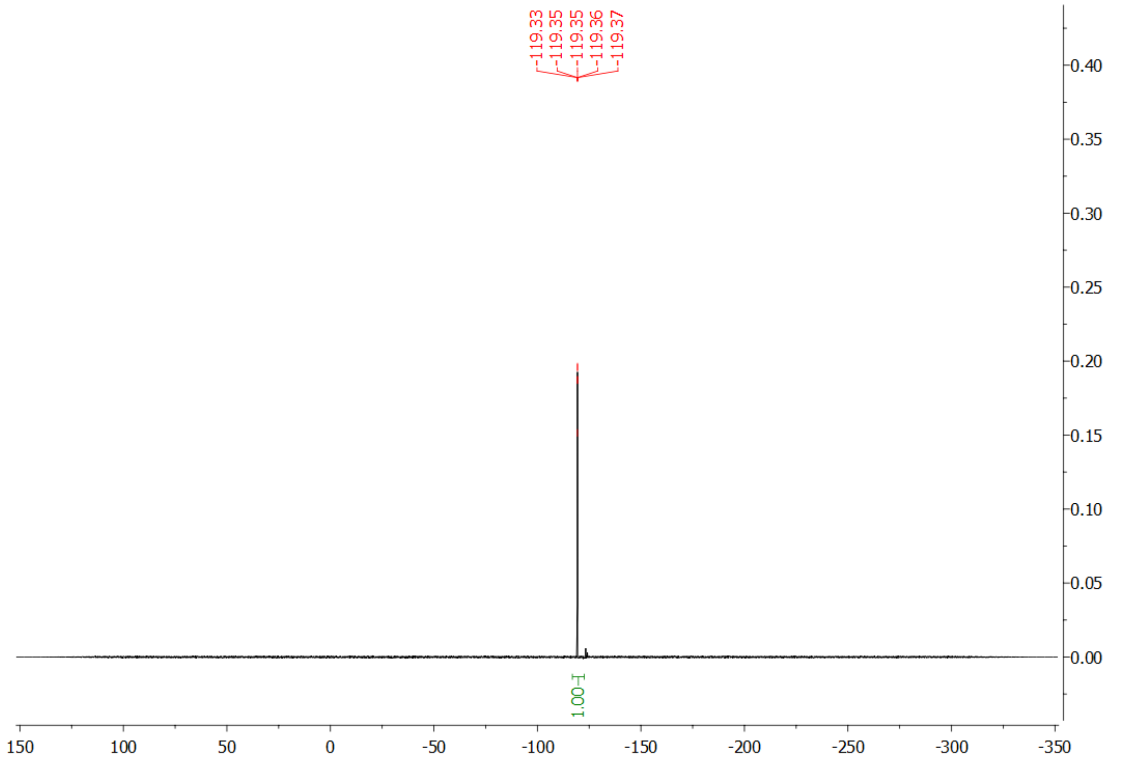
119.33
119.35
119.35
119.36
119.37
1.00

D

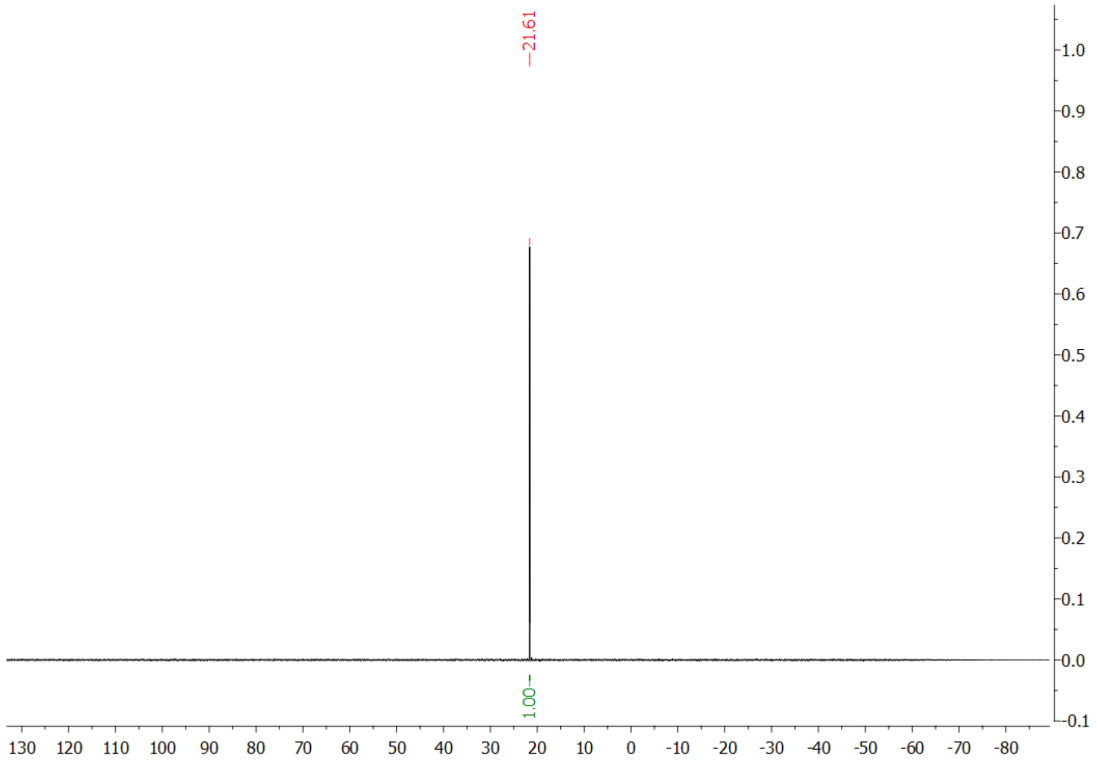

693 **Figure S10-3.** ^{1}H (**A**),^{13}C (**B**), ^{19}F (**C**), ^{31}P (**D**) NMR spectra for compound **15c**.

A

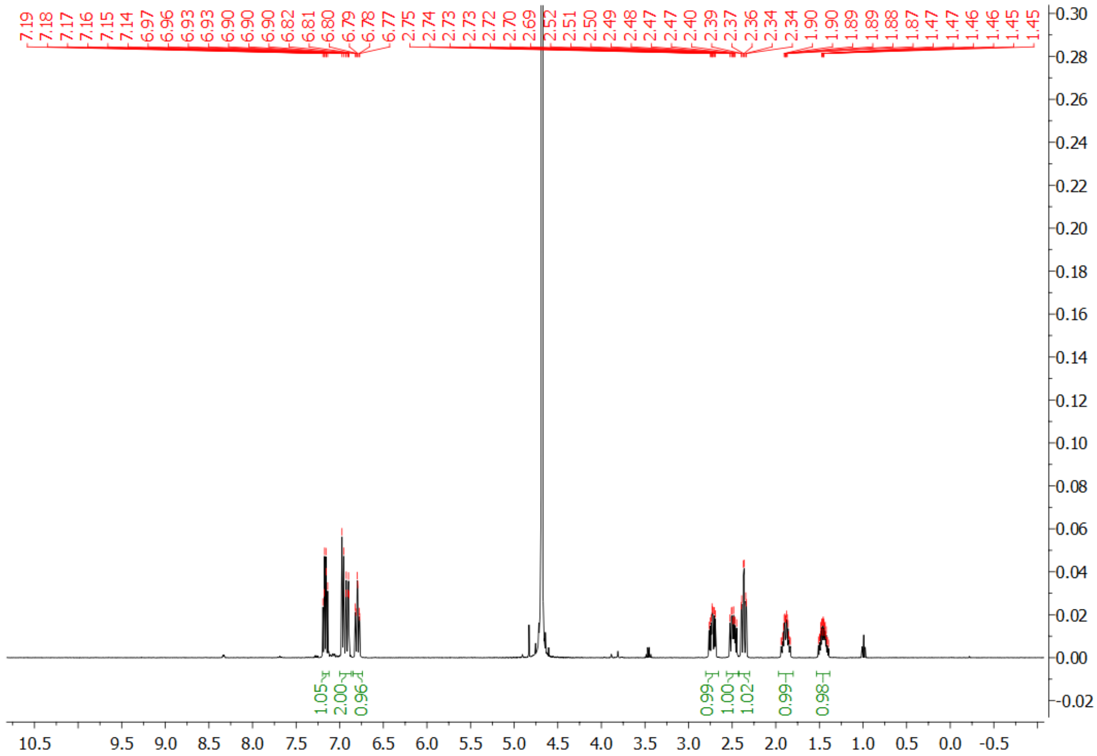

B

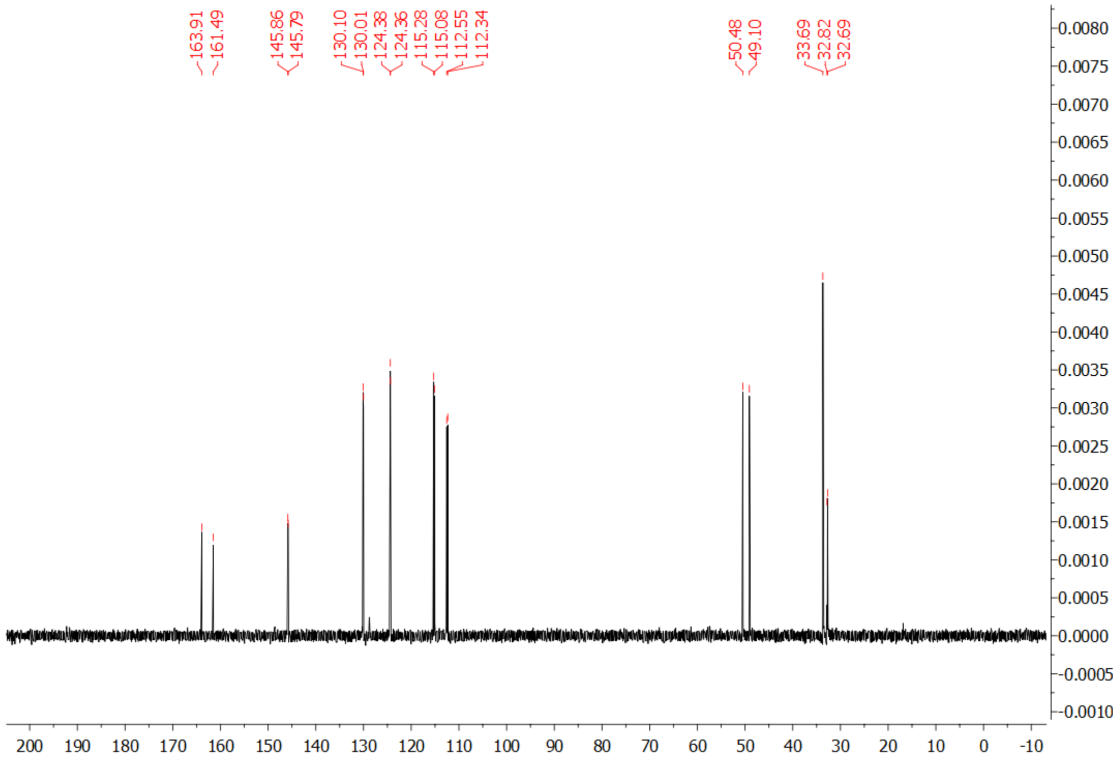
163.91
161.49
145.86
145.79
130.10
130.01
124.38
124.36
115.28
115.08
112.55
112.34
50.48
49.10
33.69
32.82
32.69

C

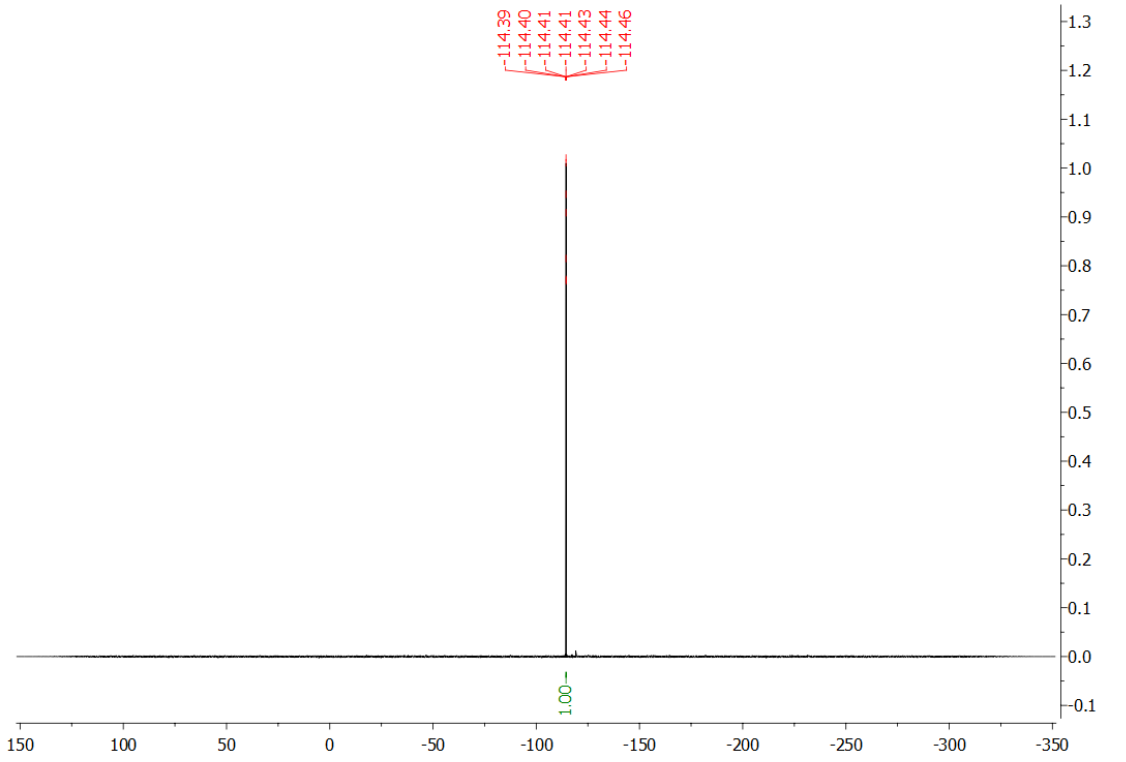
-114.39
-114.40
-114.41
-114.41
-114.43
-114.44
-114.46
1.00

D

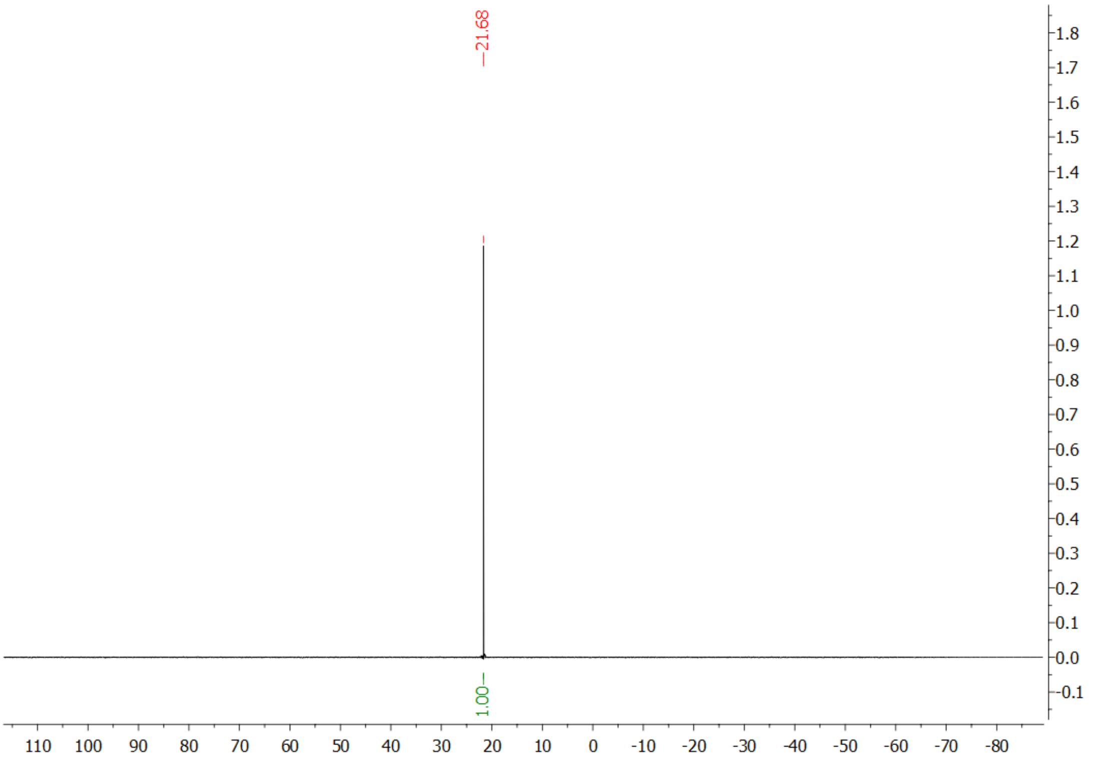

Figure S10-4. ^{1}H (**A**), ^{13}C (**B**), ^{19}F (**C**), ^{31}P (**D**) NMR spectra for compound **15d**.

A

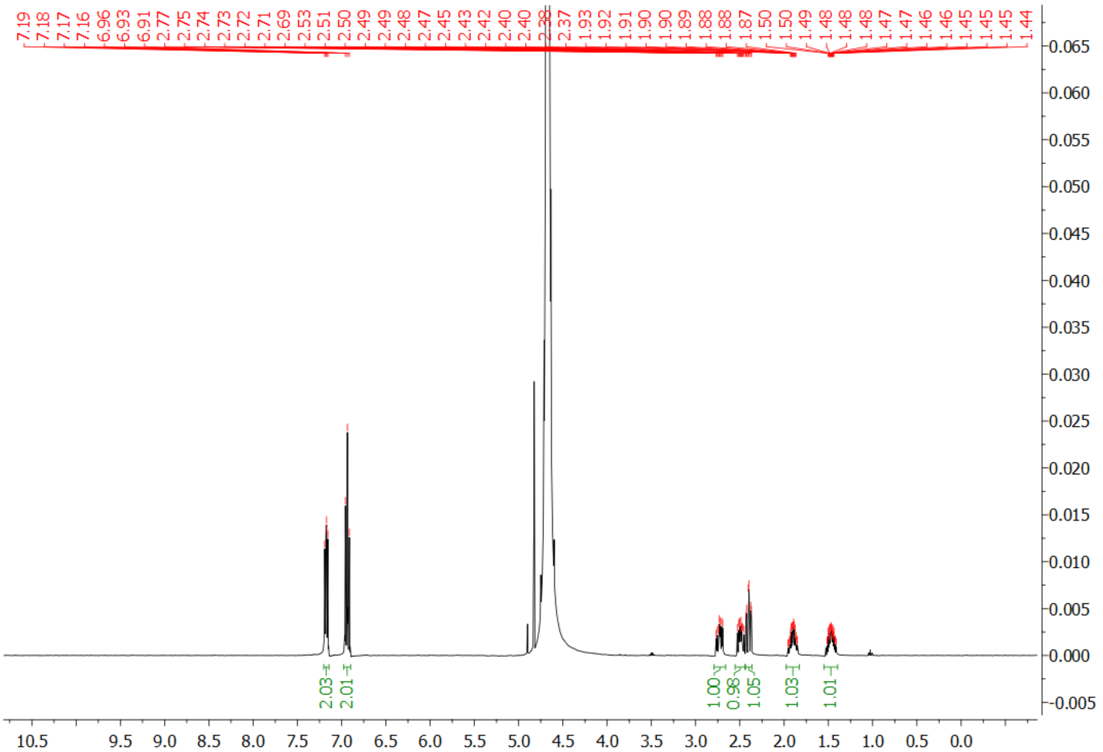

B

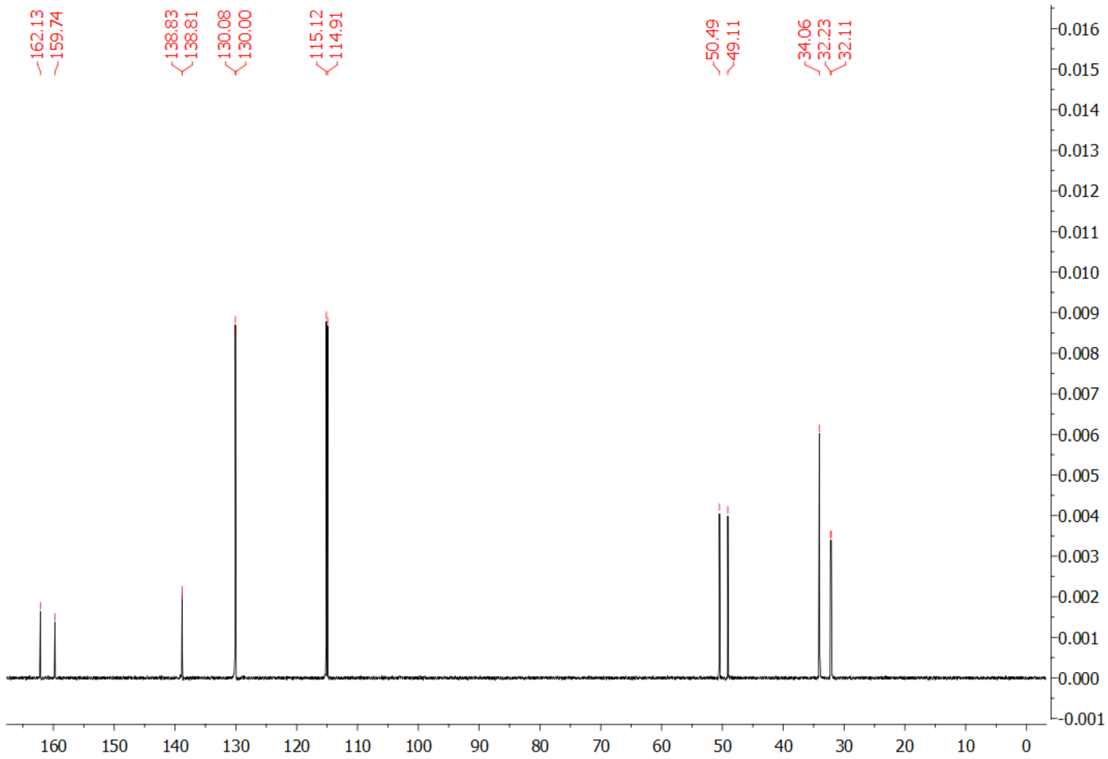
162.13
159.74
138.83
138.81
130.08
130.00
115.12
114.91
50.49
49.11
34.06
32.23
32.11
0.016
0.015
0.014
0.013
0.012
0.011
0.010
0.009
0.008
0.007
0.006
0.005
0.004
0.003
0.002
0.001
0.000
-0.001
160
150
140
130
120
110
100
90
80
70
60
50
40
30
20
10
0

C

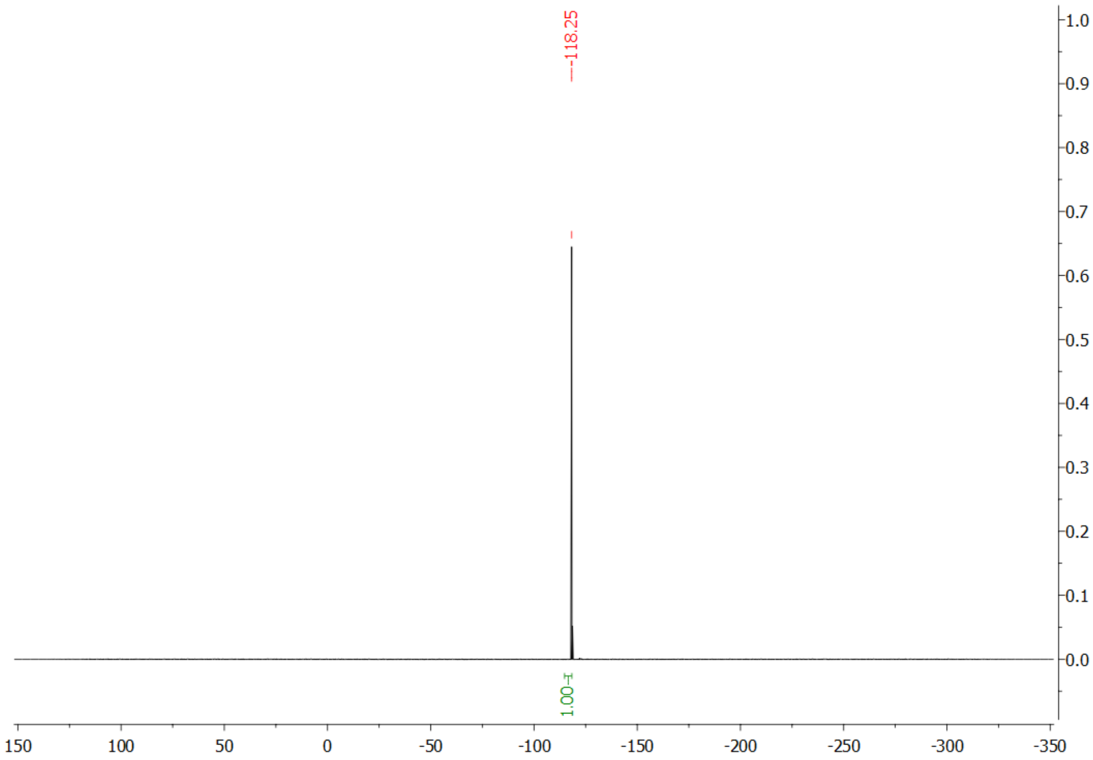
-118.25
1.00
1.0
0.9
0.8
0.7
0.6
0.5
0.4
0.3
0.2
0.1
0.0
150
100
50
0
-50
-100
-150
-200
-250
-300
-350

D

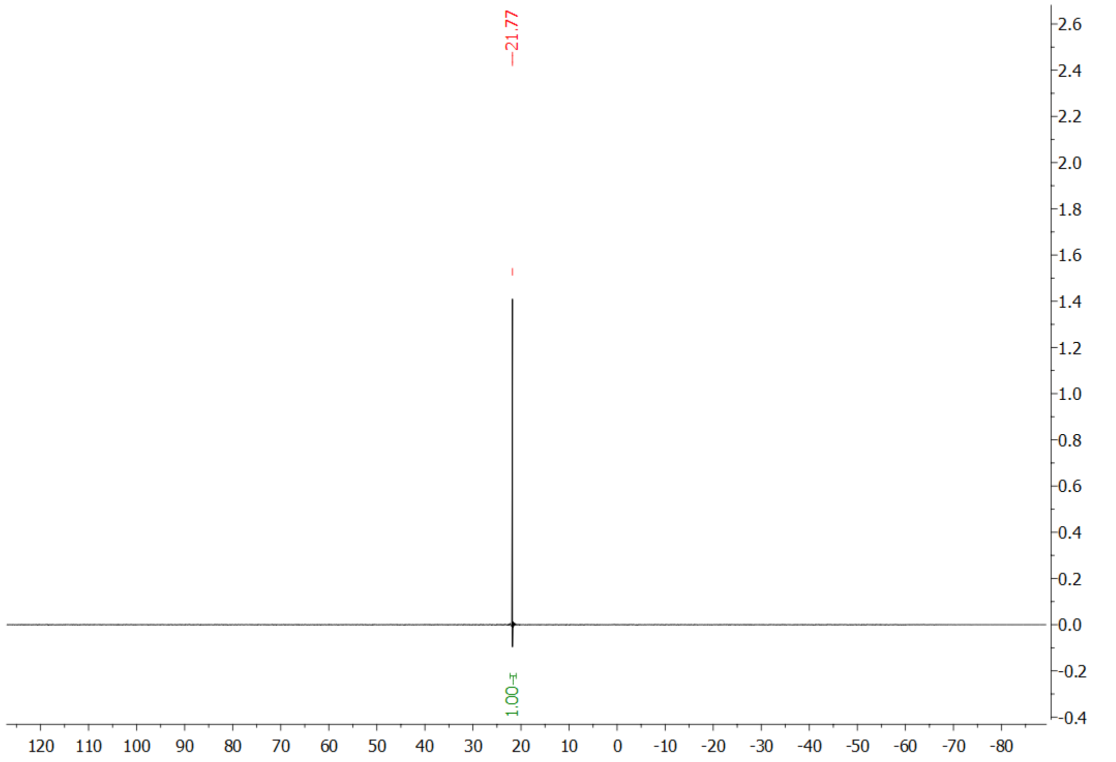

695 **Figure S10-5.** ^{1}H (**A**),^{13}C (**B**), ^{19}F (**C**), ^{31}P (**D**) NMR spectra for compound **15e**.

A

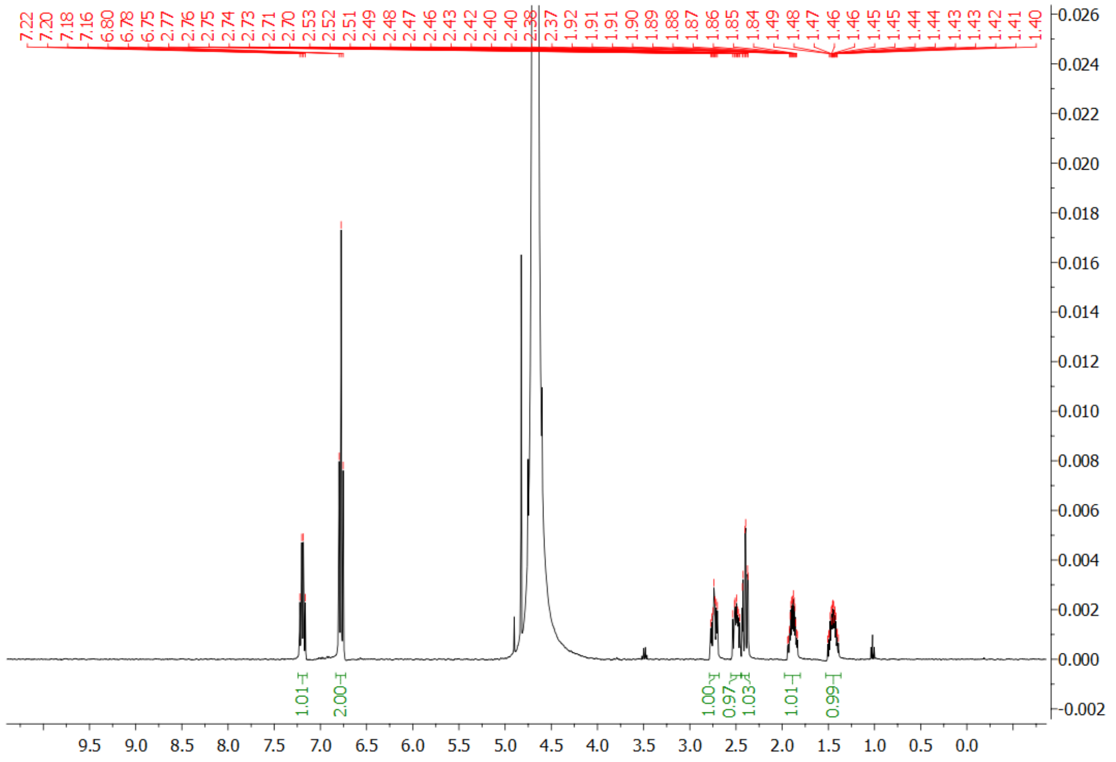

B

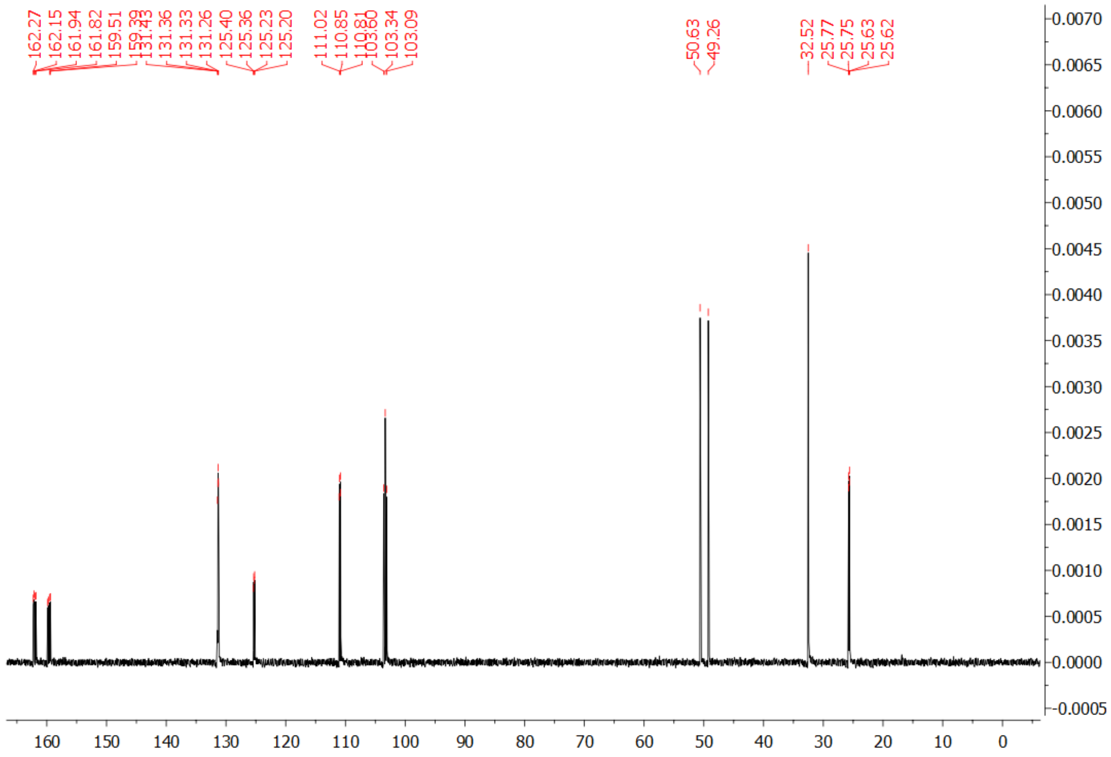

C

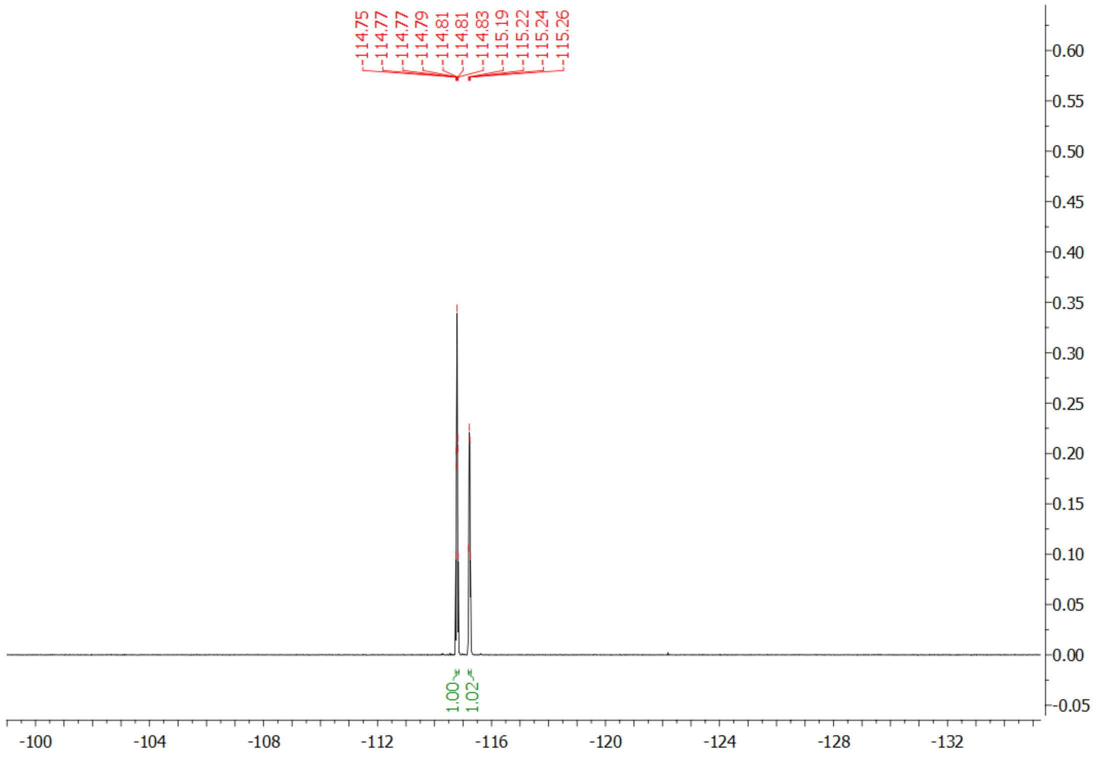

D

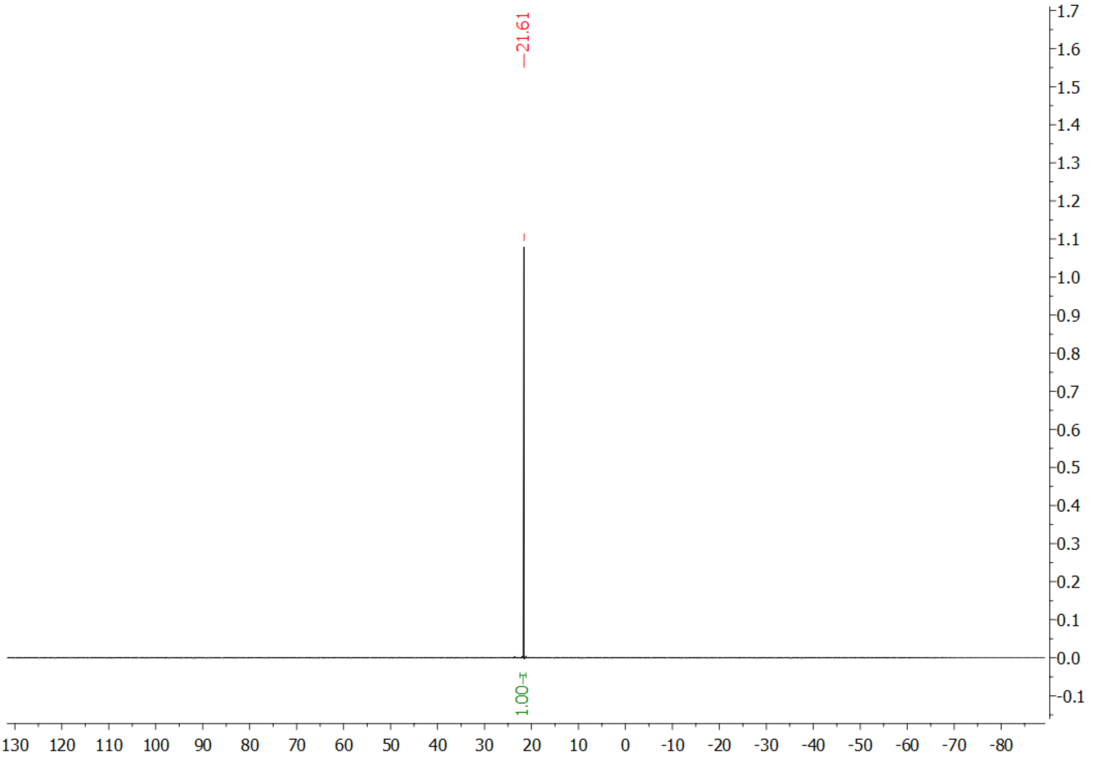

696 **Figure S10-6.** ^{1}H (**A**), ^{13}C (**B**), ^{19}F (**C**), ^{31}P (**D**) NMR spectra for compound **15f**.

A

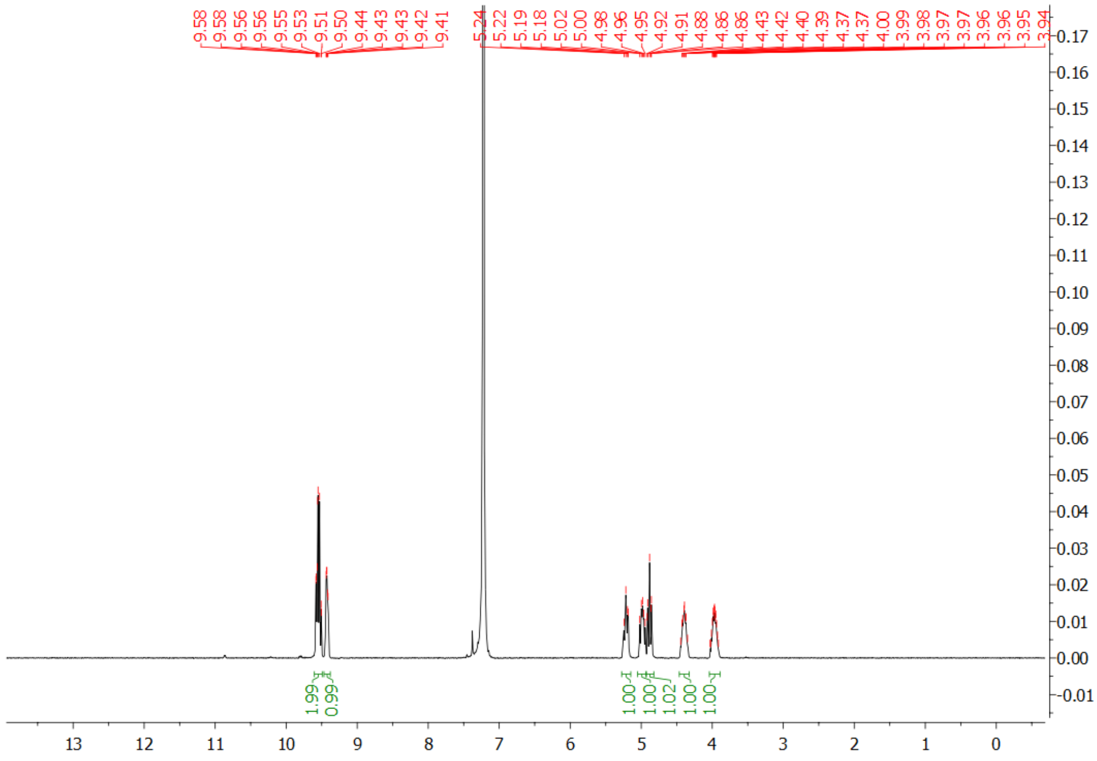

B

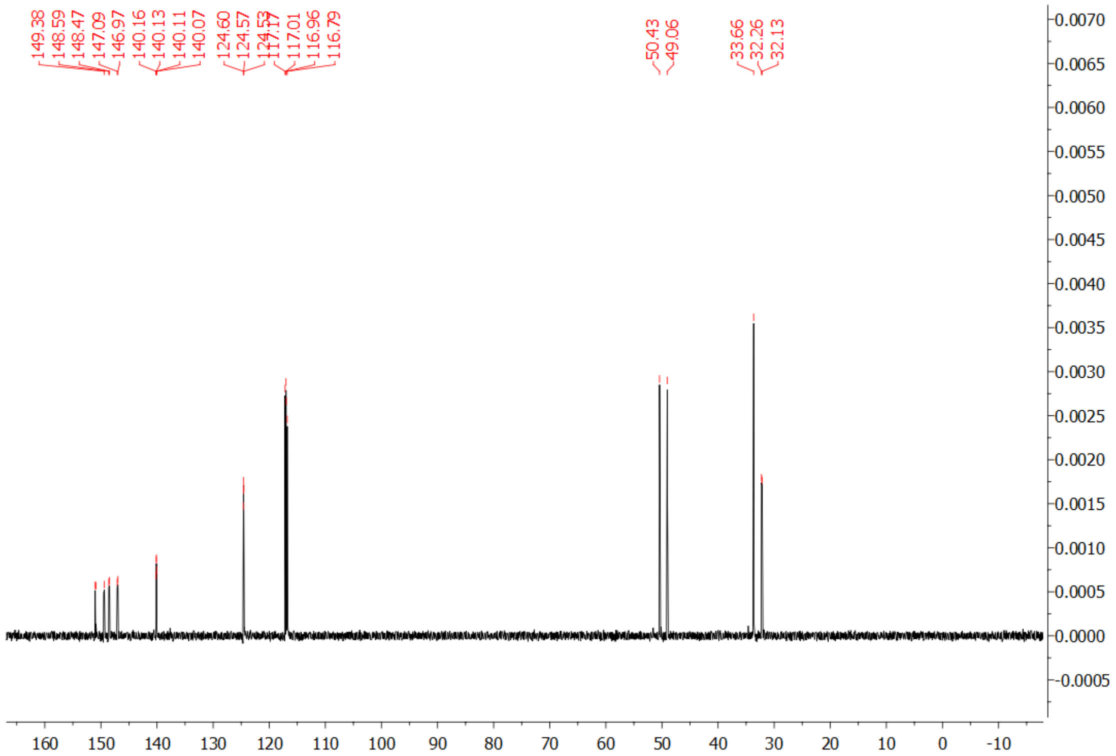
149.38
148.59
148.47
147.09
146.97
140.16
140.13
140.11
140.07
124.60
124.57
124.53
117.17
117.01
116.96
116.79
50.43
49.06
33.66
32.26
32.13

C

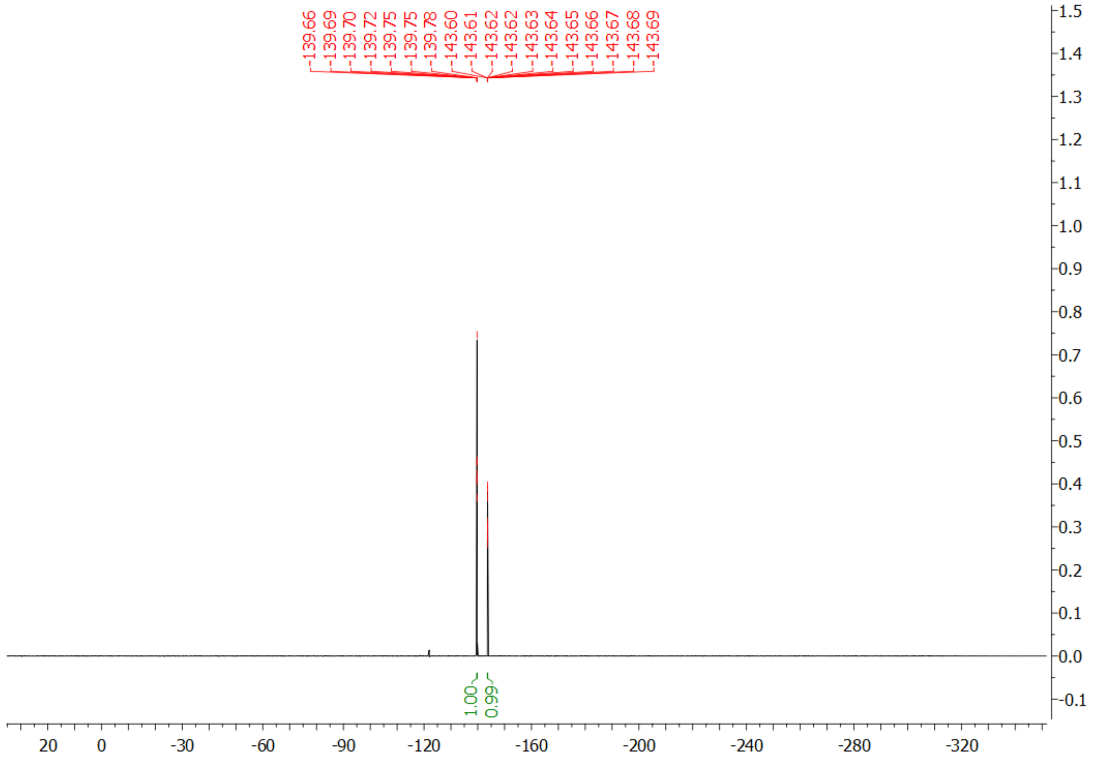
-139.66
-139.69
-139.70
-139.72
-139.75
-139.75
-139.78
-143.60
-143.61
-143.62
-143.62
-143.63
-143.64
-143.65
-143.66
-143.67
-143.68
-143.69
1.00
0.99

D

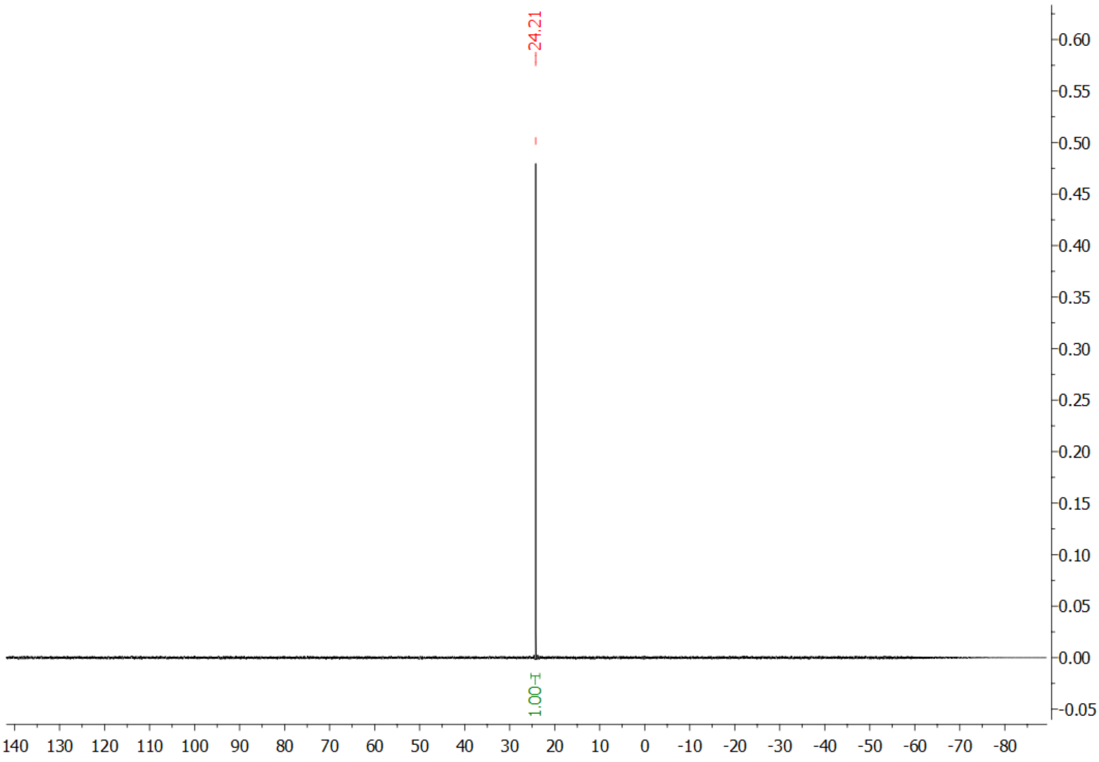

697 **Figure S10-7.** ^{1}H (**A**),^{13}C (**B**), ^{19}F (**C**), ^{31}P (**D**) NMR spectra for compound **15g**.

A

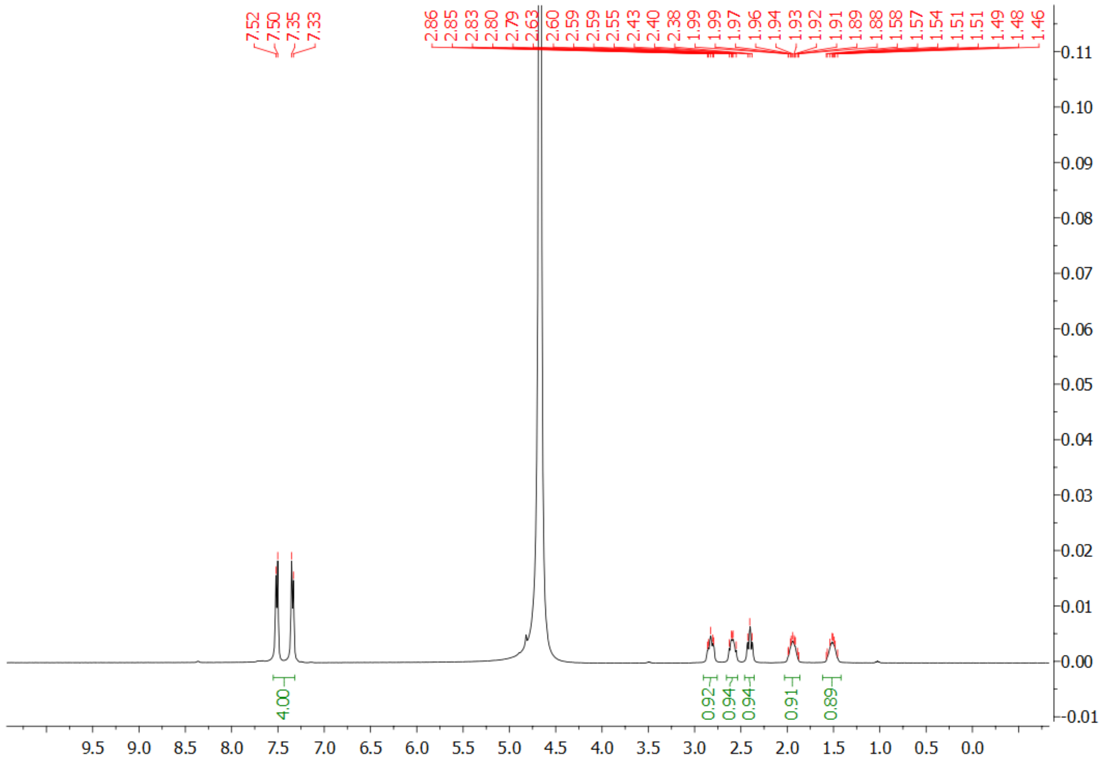

B

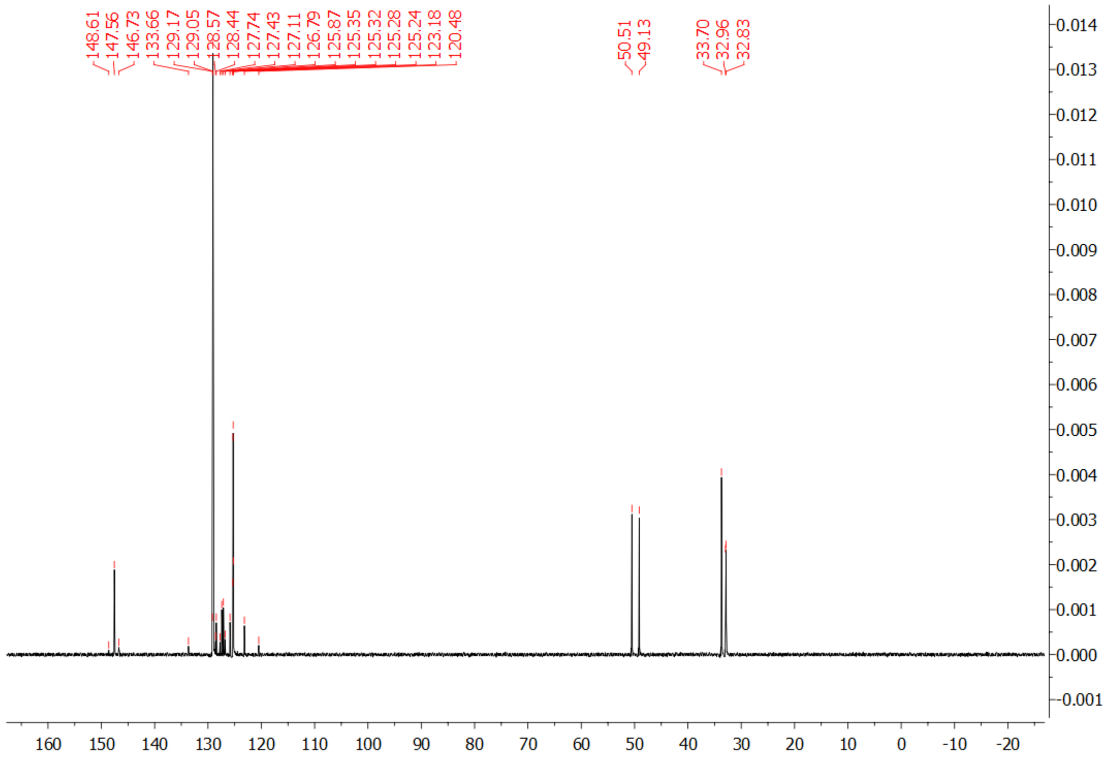
148.61
147.56
146.73
133.66
129.17
129.05
128.57
128.44
127.74
127.43
127.11
126.79
125.87
125.35
125.32
125.28
125.24
123.18
120.48
50.51
49.13
33.70
32.96
32.83

C

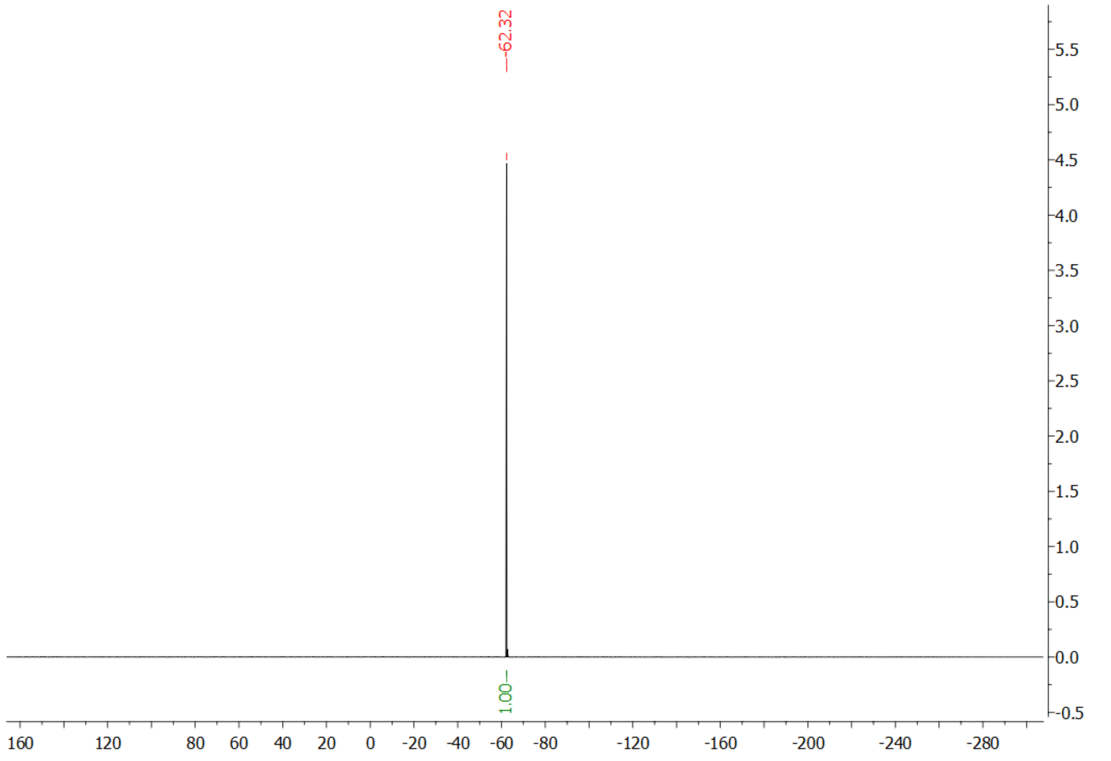
-62.32
1.00

D

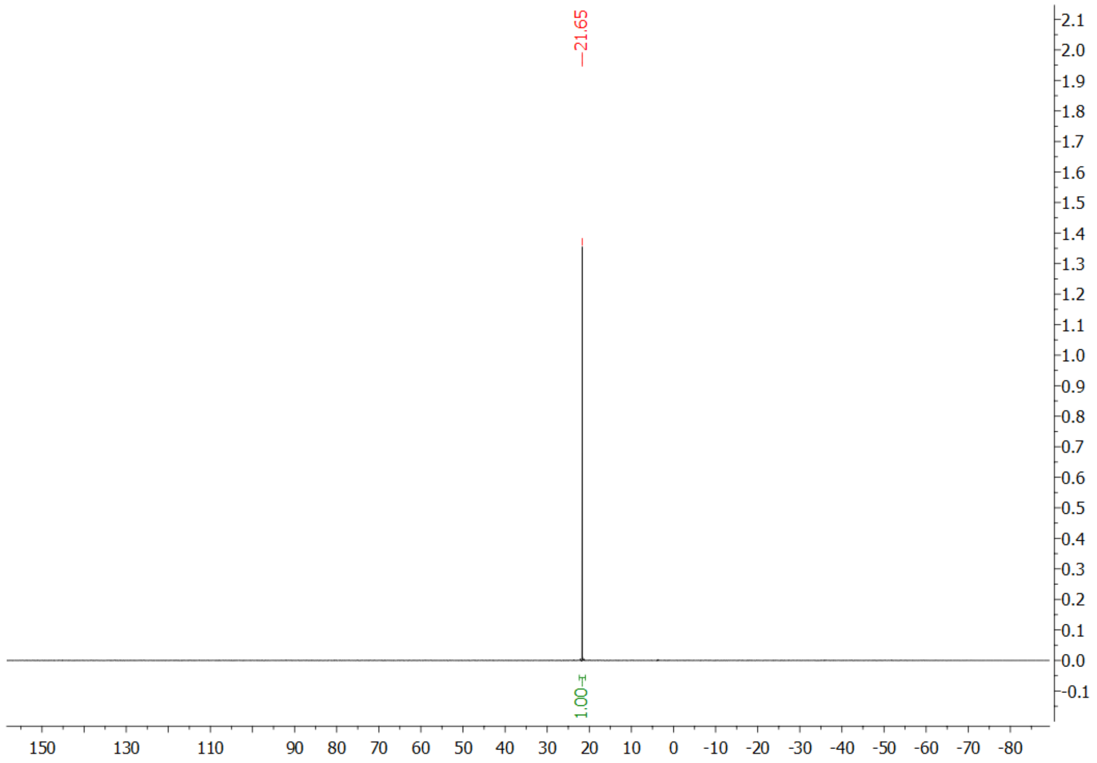

698 **Figure S10-8.** ^{1}H (**A**), ^{13}C (**B**), ^{19}F (**C**), ^{31}P (**D**) NMR spectra for compound **15h**.

A

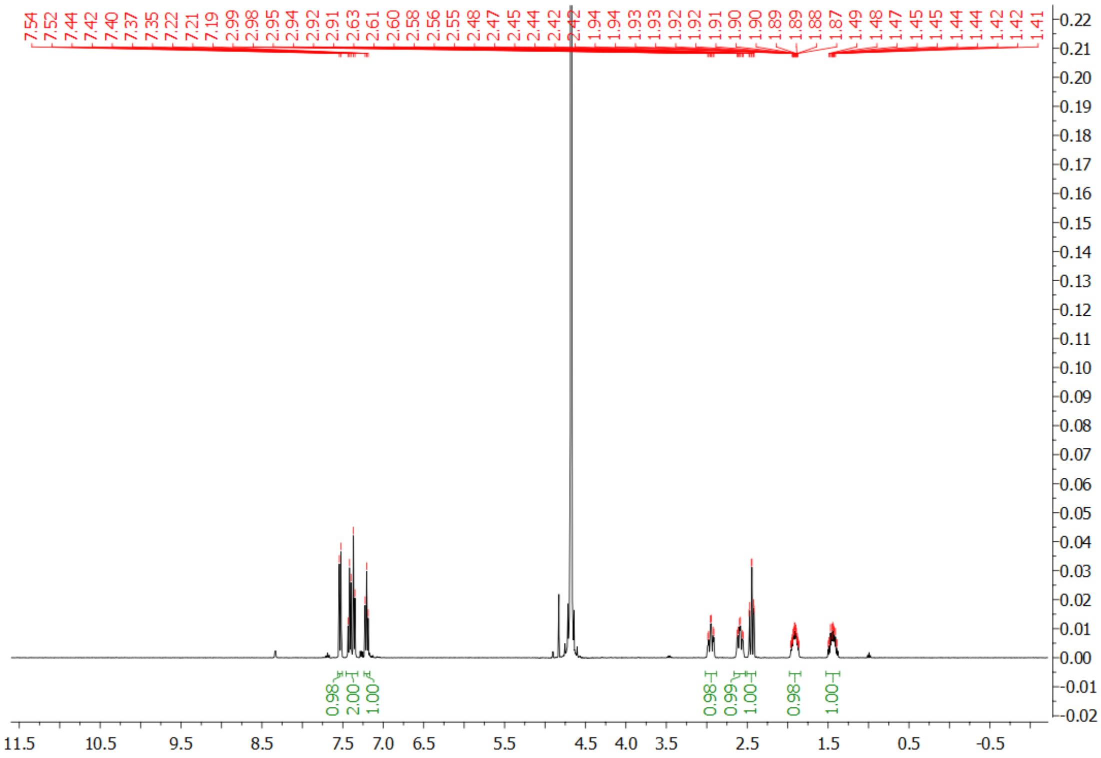

B

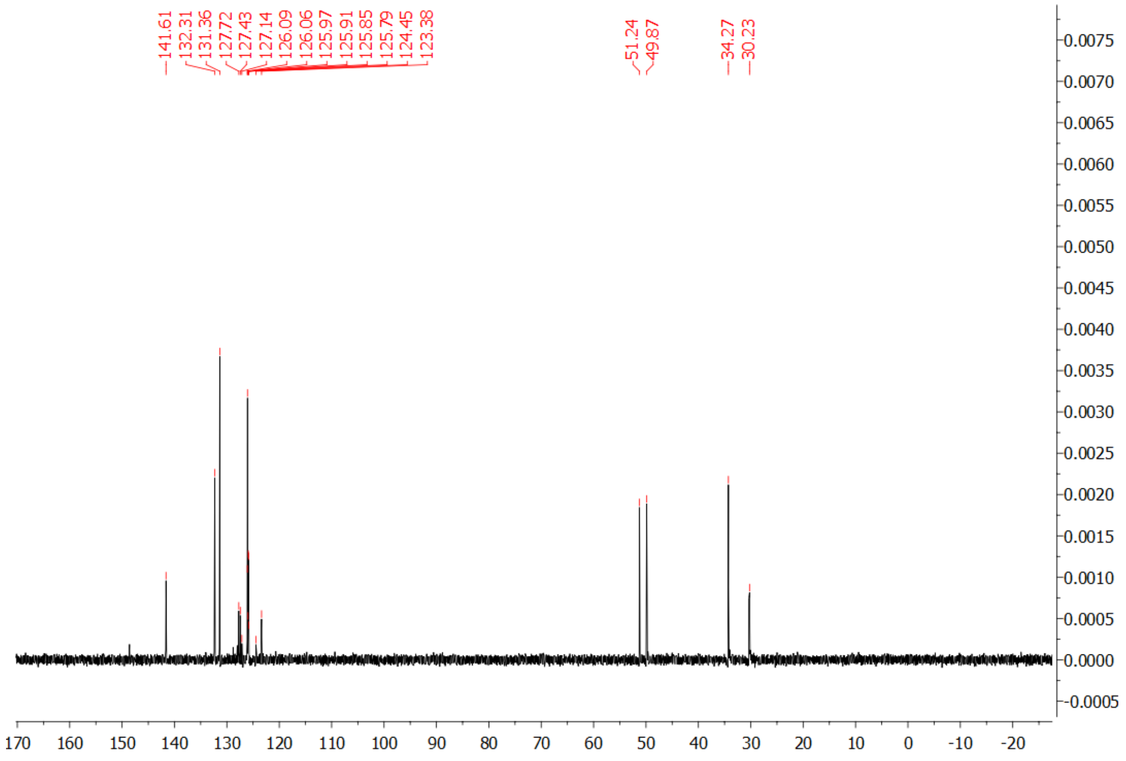
141.61
132.31
131.36
127.72
127.43
127.14
126.09
126.06
125.97
125.91
125.85
125.79
124.45
123.38
51.24
49.87
34.27
30.23

C

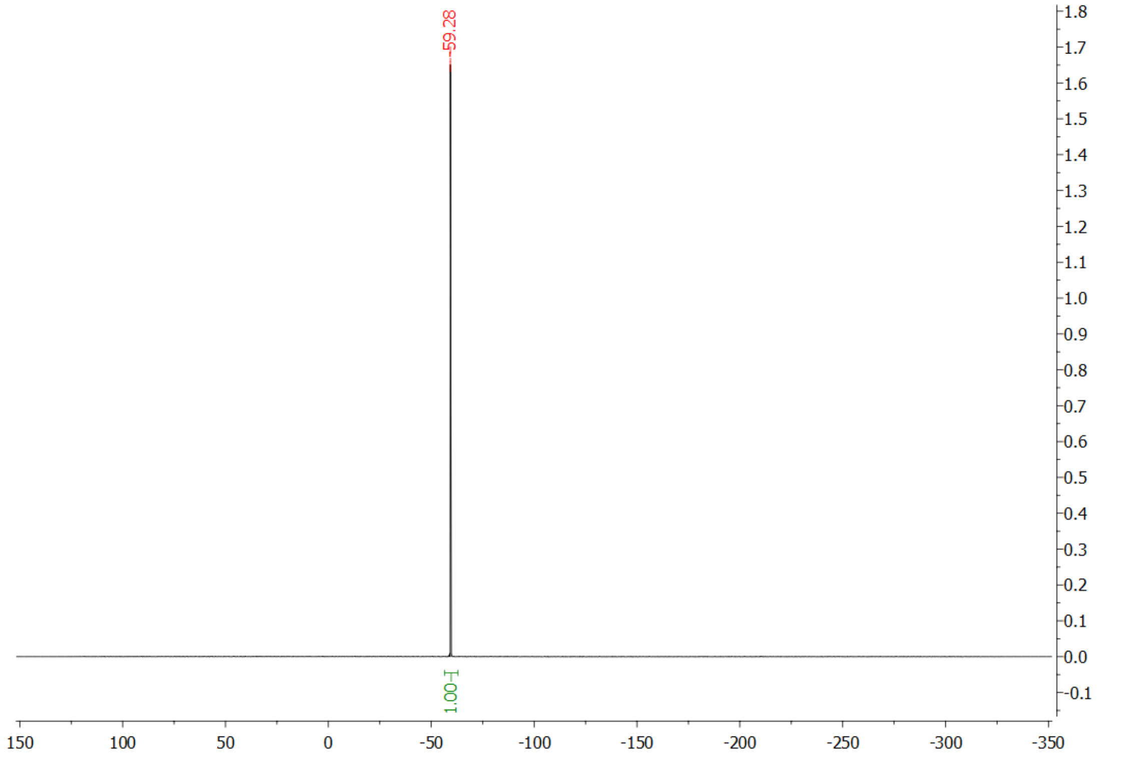
-59.28
1.00

D

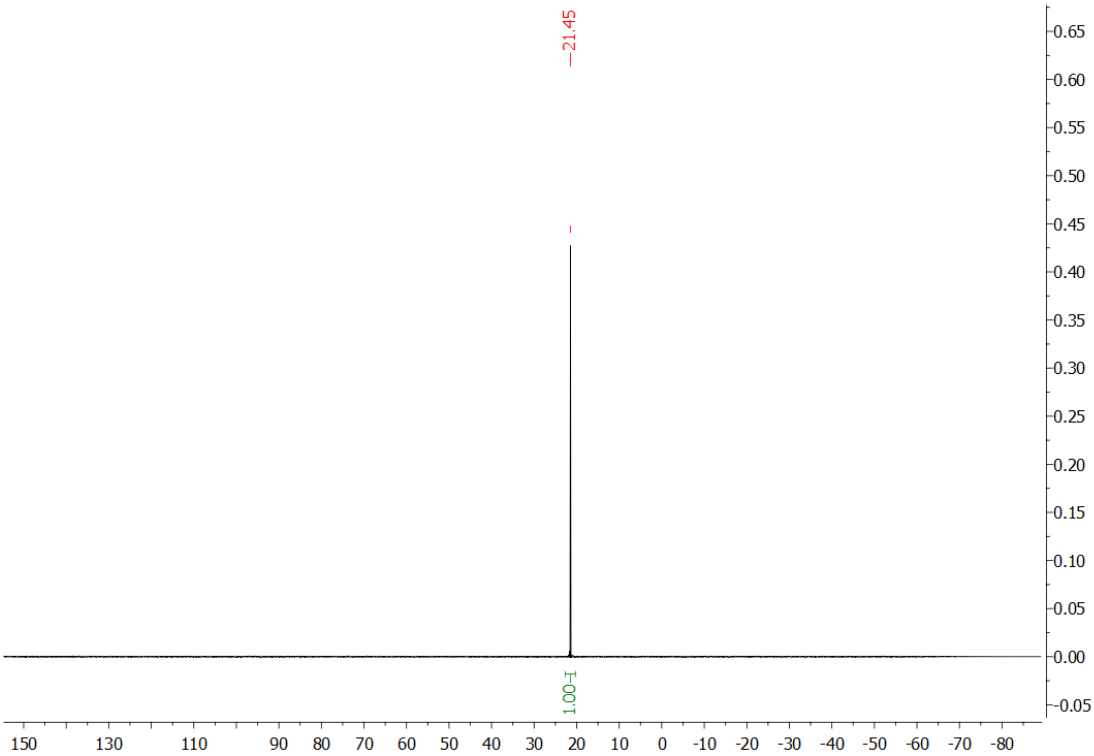

699 **Figure S10-9.** ^{1}H (**A**), ^{13}C (**B**), ^{19}F (**C**), ^{31}P (**D**) NMR spectra for compound **17a**.

A

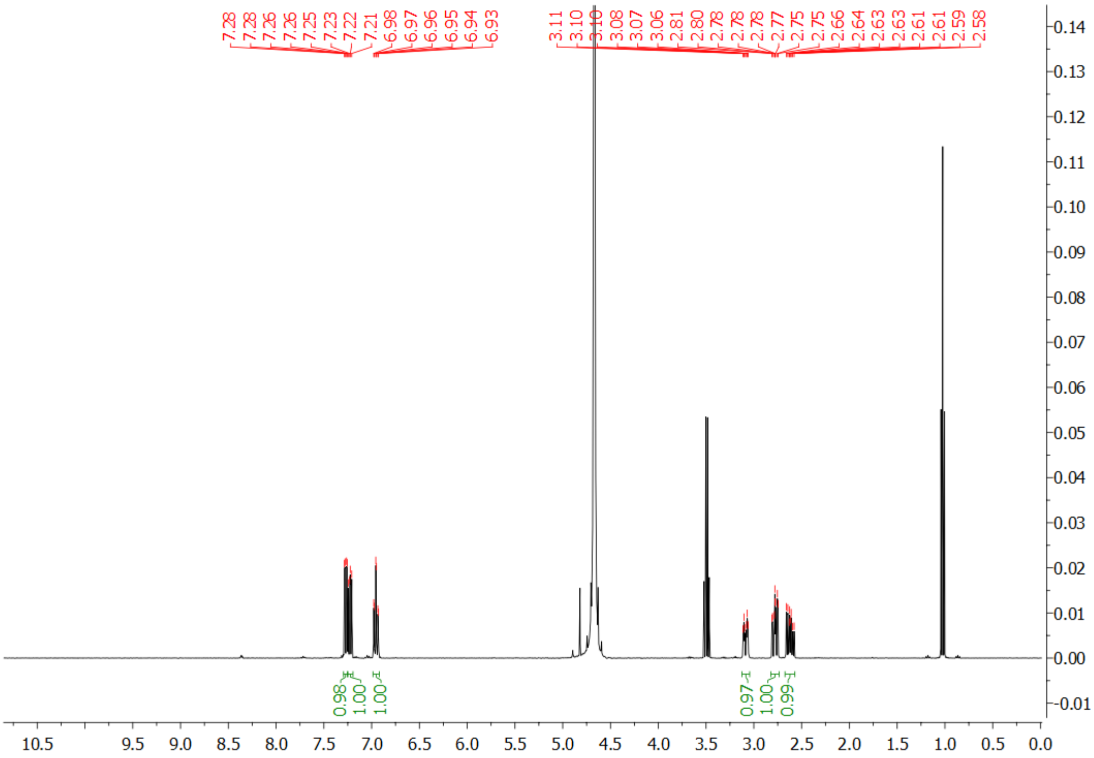

B

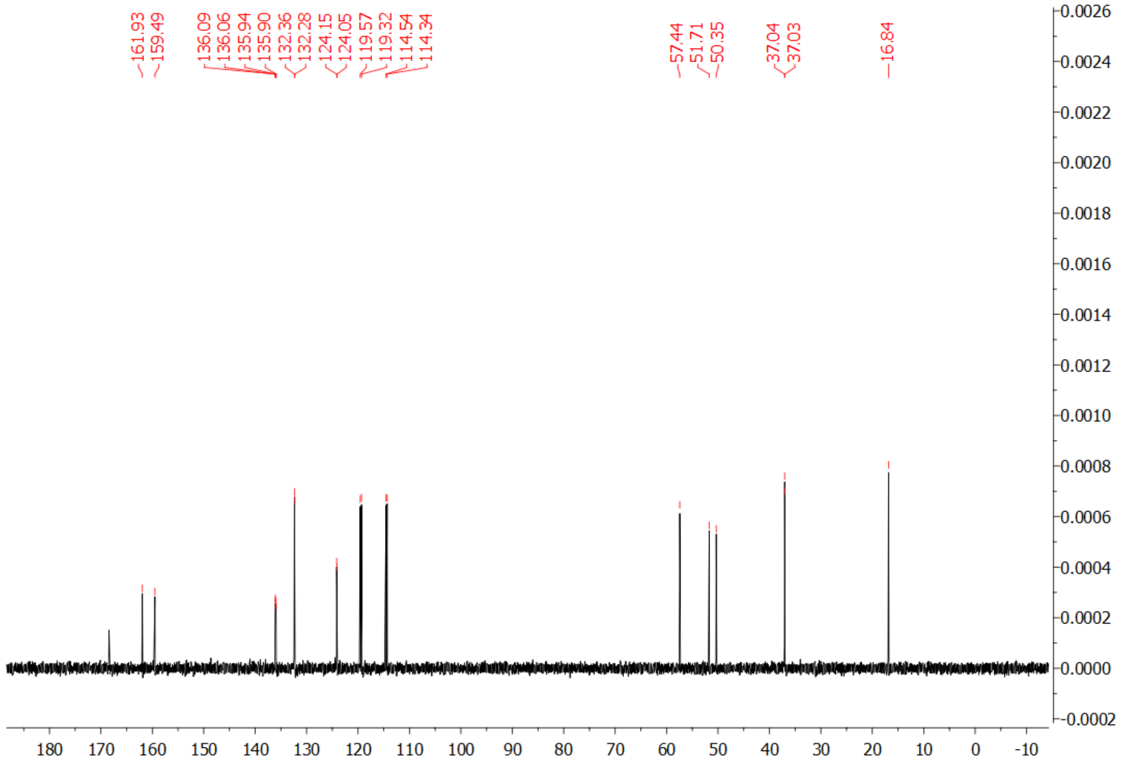
161.93
159.49
136.09
136.06
135.94
135.90
132.36
132.28
124.15
124.05
119.57
119.32
114.54
114.34
57.44
51.71
50.35
37.04
37.03
16.84

C

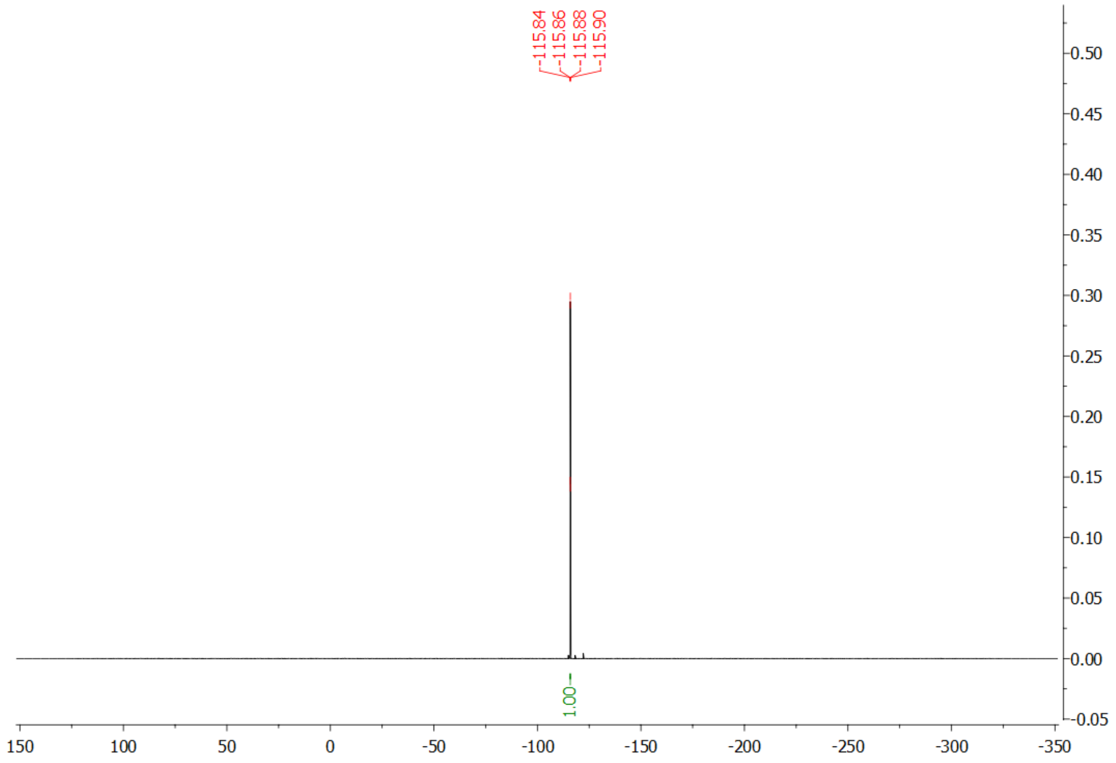
-115.84
-115.86
-115.88
-115.90
1.00

D

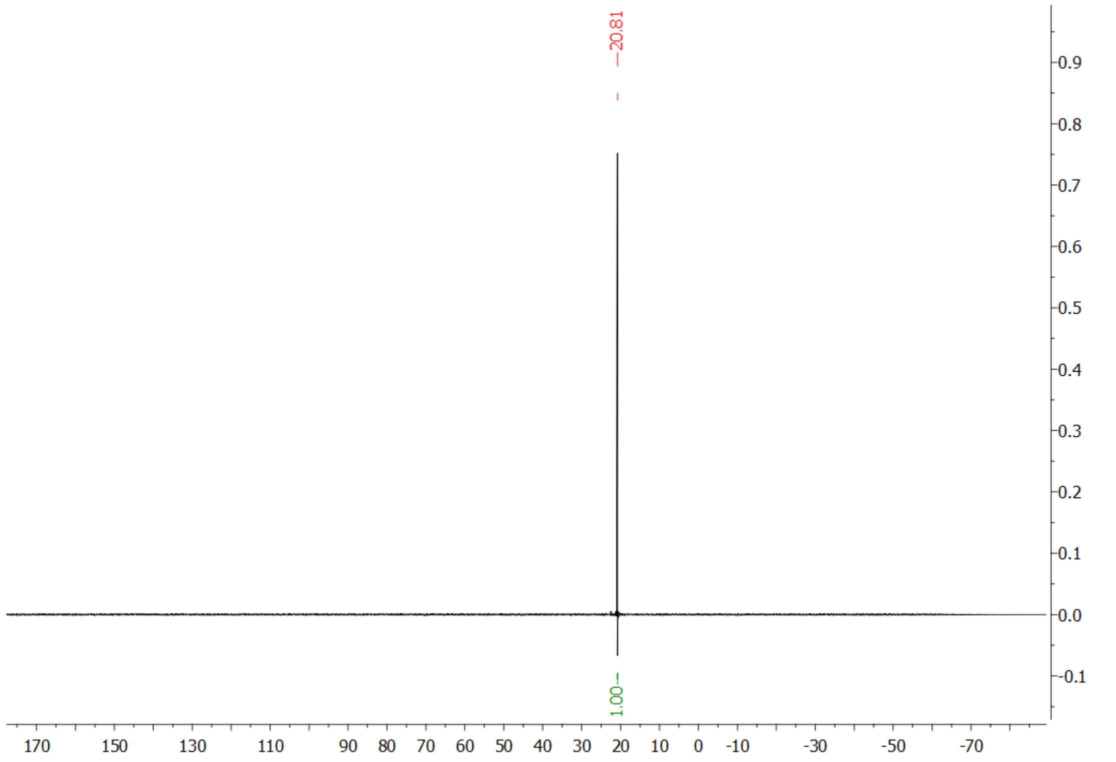

Figure S10-10. ^{1}H (**A**), ^{19}F (**B**), ^{31}P (**C**) NMR spectra for compound **17b**.

A

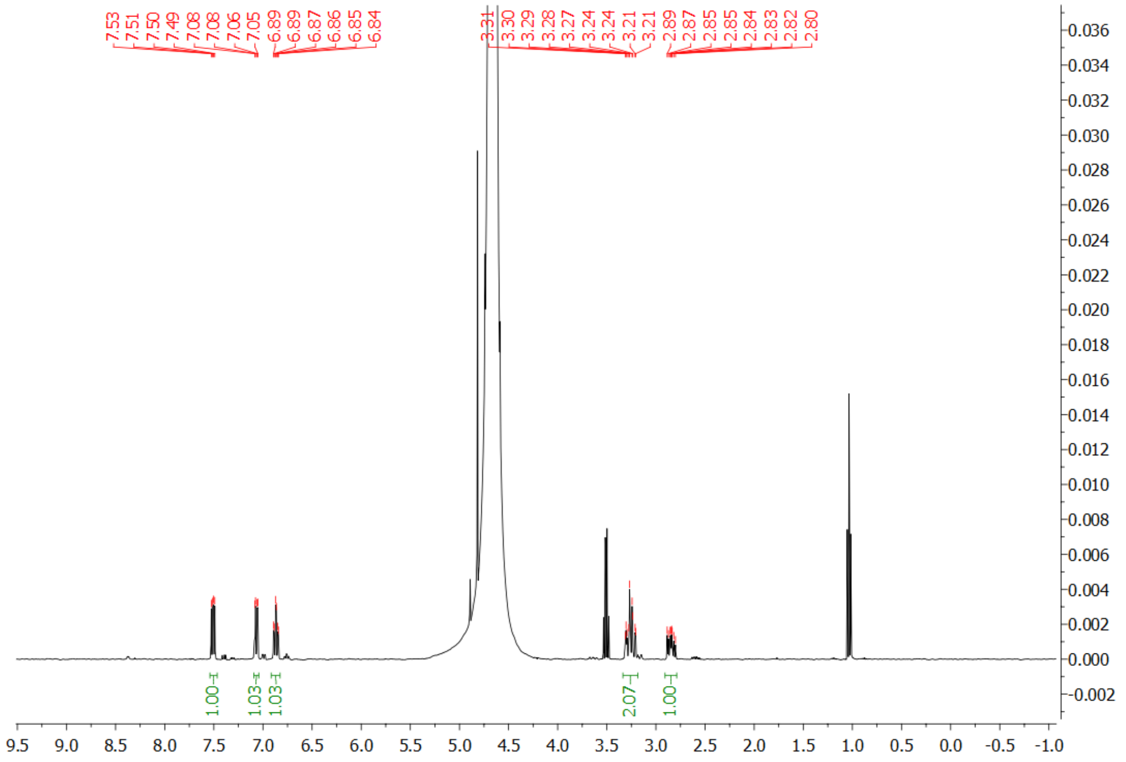

B

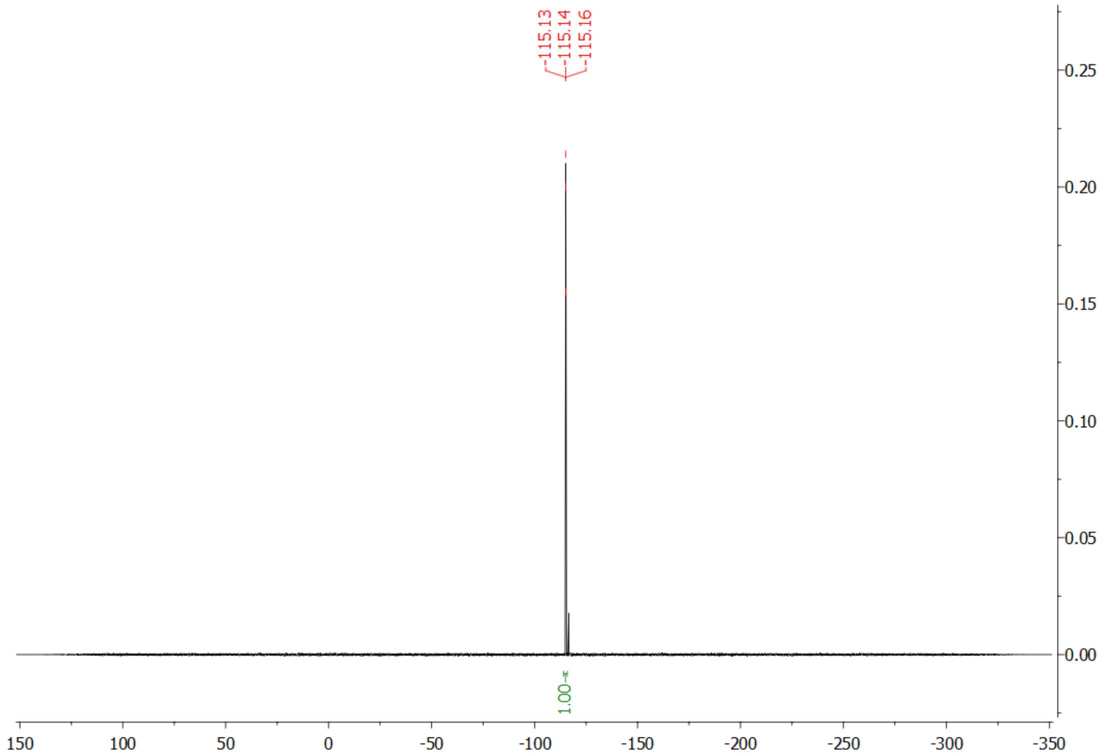
-115.13
-115.14
-115.16
1.00
0.25
0.20
0.15
0.10
0.05
0.00
150 100 50 0 -50 -100 -150 -200 -250 -300 -350

C

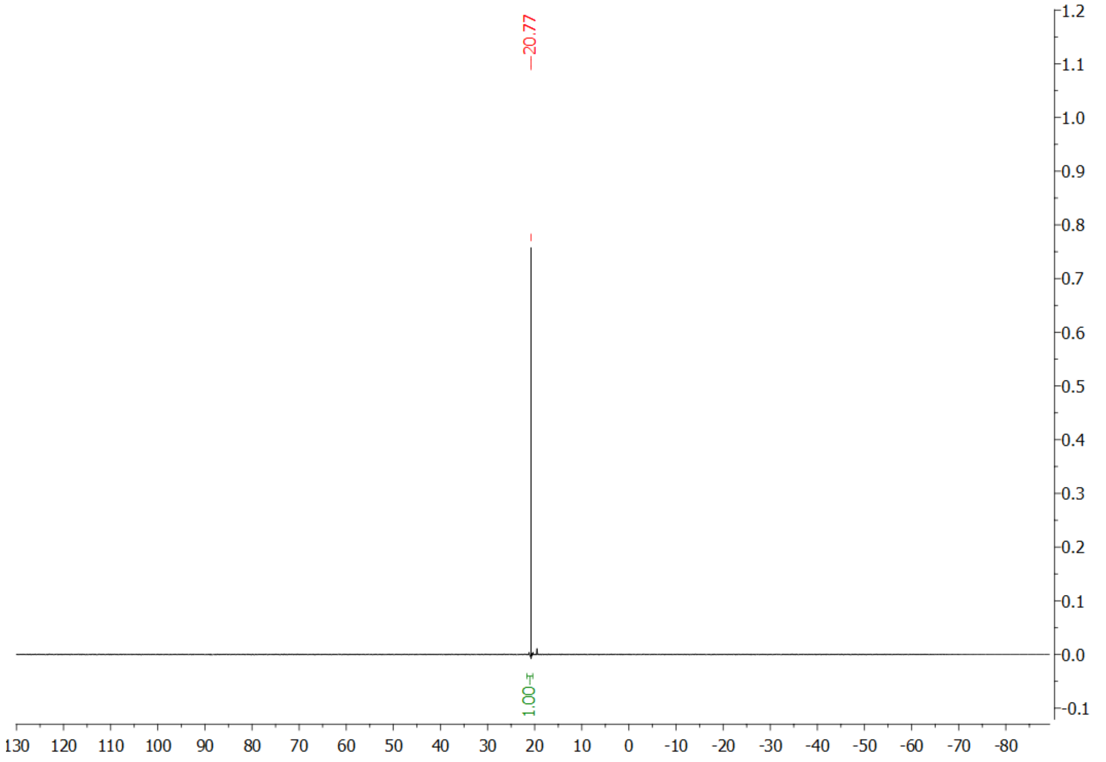
20.77
1.00
1.2
1.1
1.0
0.9
0.8
0.7
0.6
0.5
0.4
0.3
0.2
0.1
0.0
-0.1
130 120 110 100 90 80 70 60 50 40 30 20 10 0 -10 -20 -30 -40 -50 -60 -70 -80

707

708 **Figure S10-11.** ^{1}H (**A**),^{13}C (**B**), ^{19}F (**C**), ^{31}P (**D**) NMR spectra for compound **17c**.

A

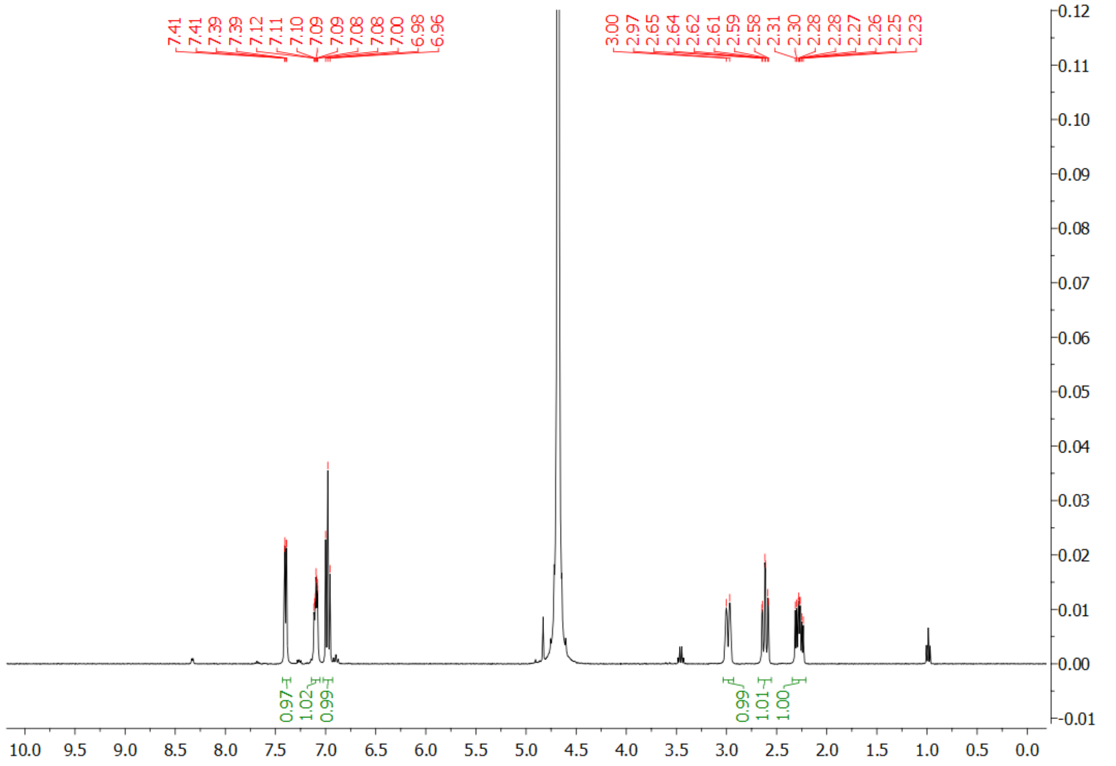

B

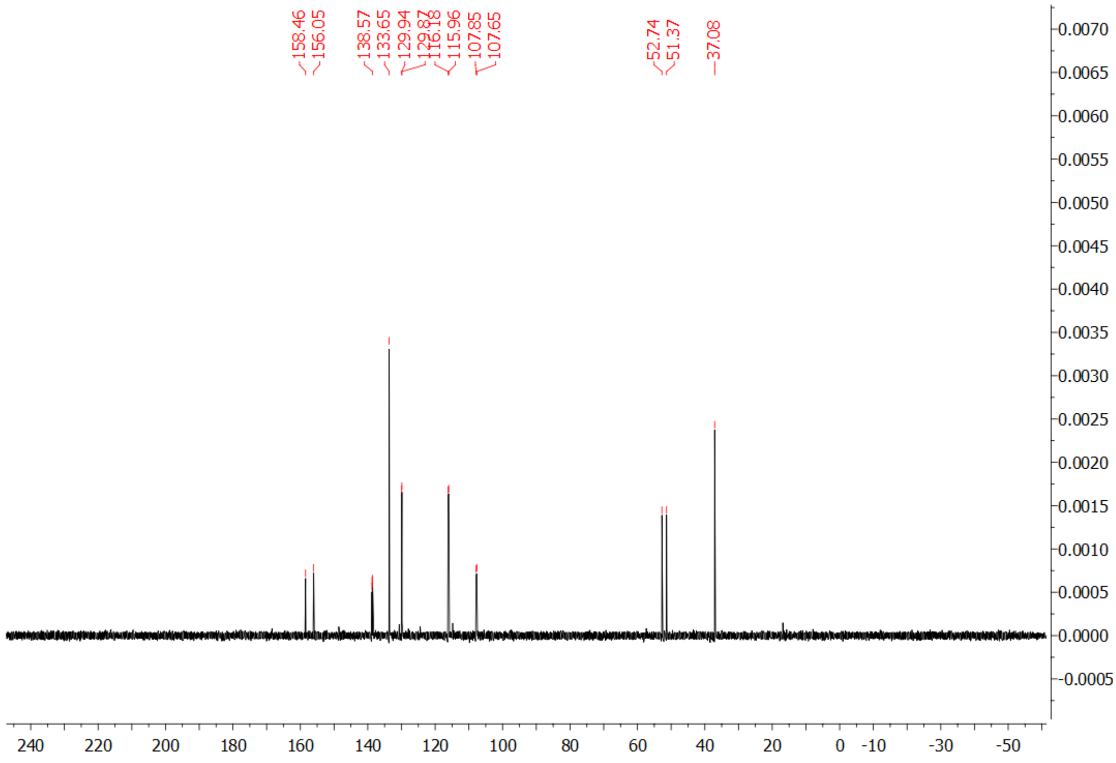

C

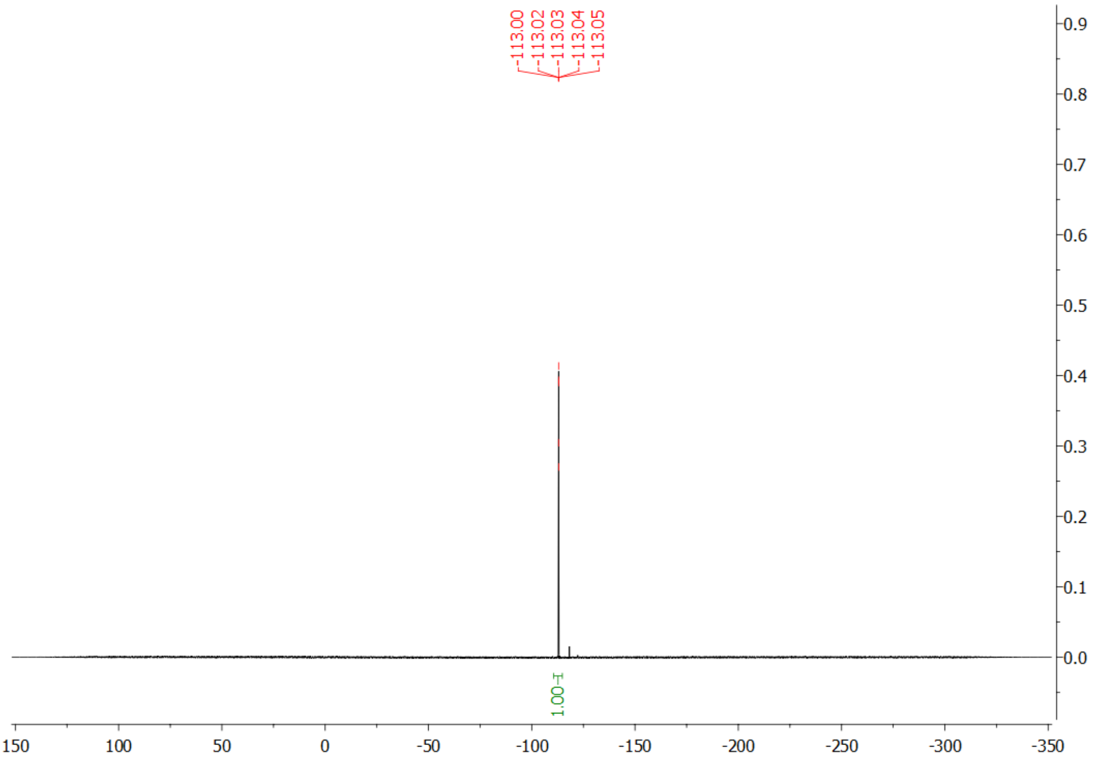

D

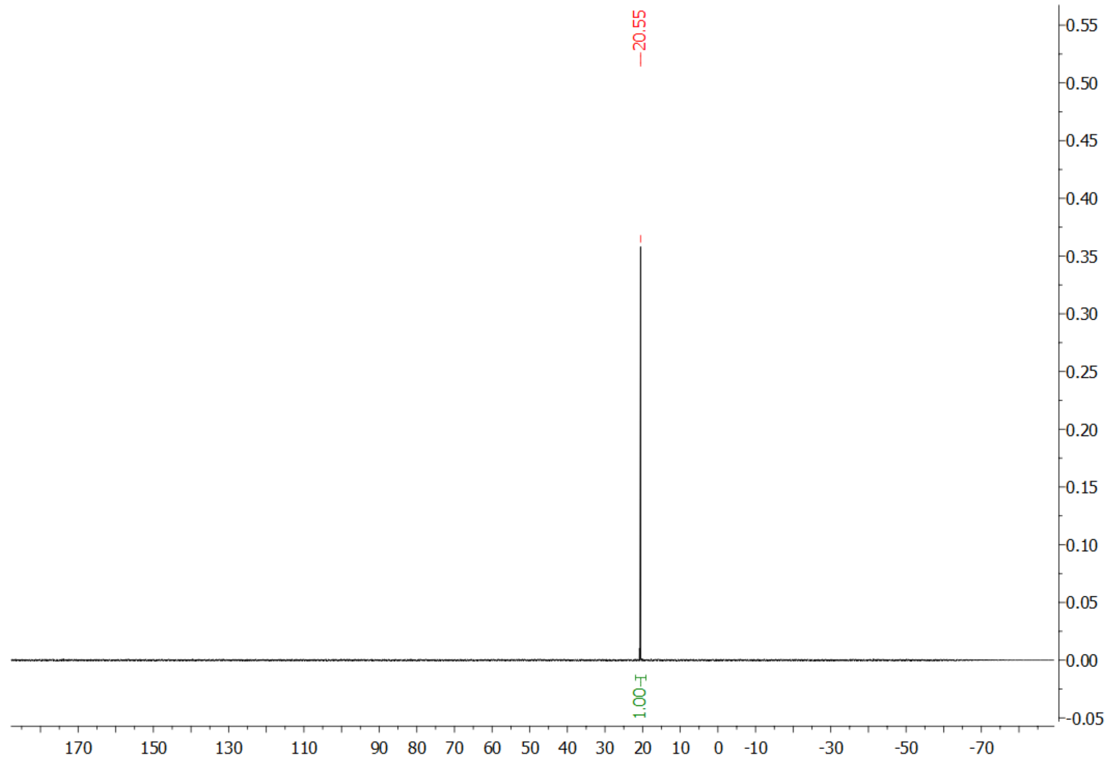

Figure S10-12. ^{1}H (**A**), ^{13}C (**B**), ^{19}F (**C**), ^{31}P (**D**) NMR spectra and HPLC (**E**) for compound **17d**.

A

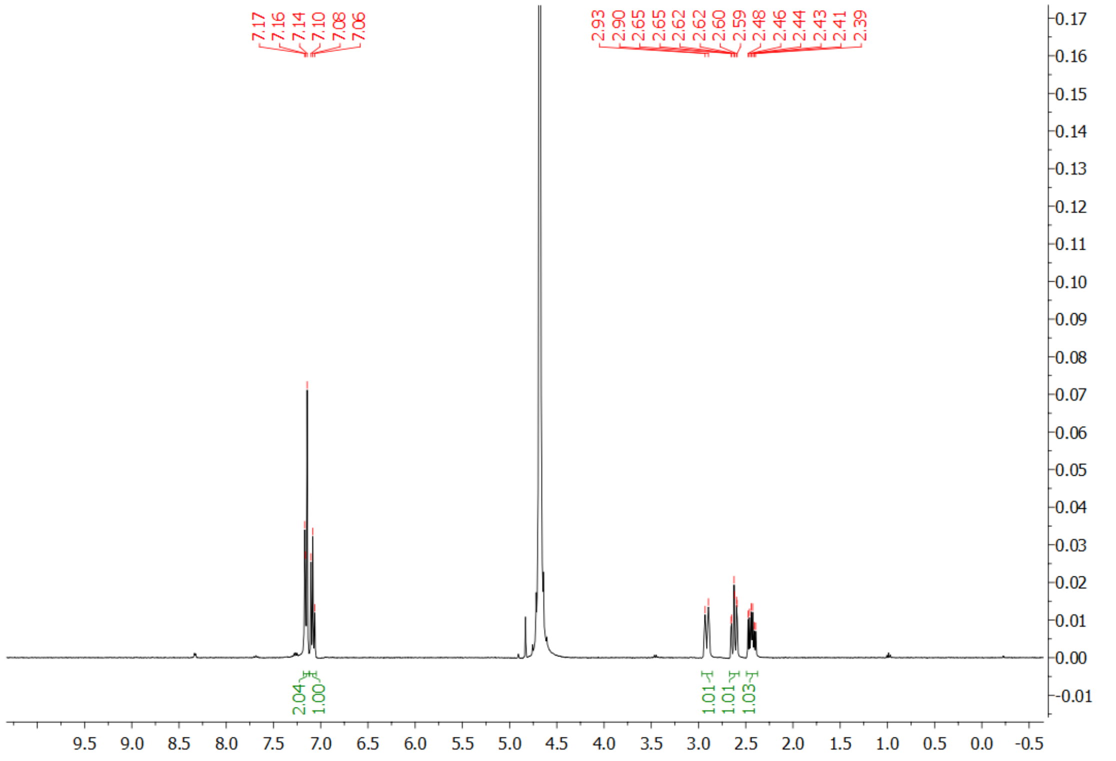

B

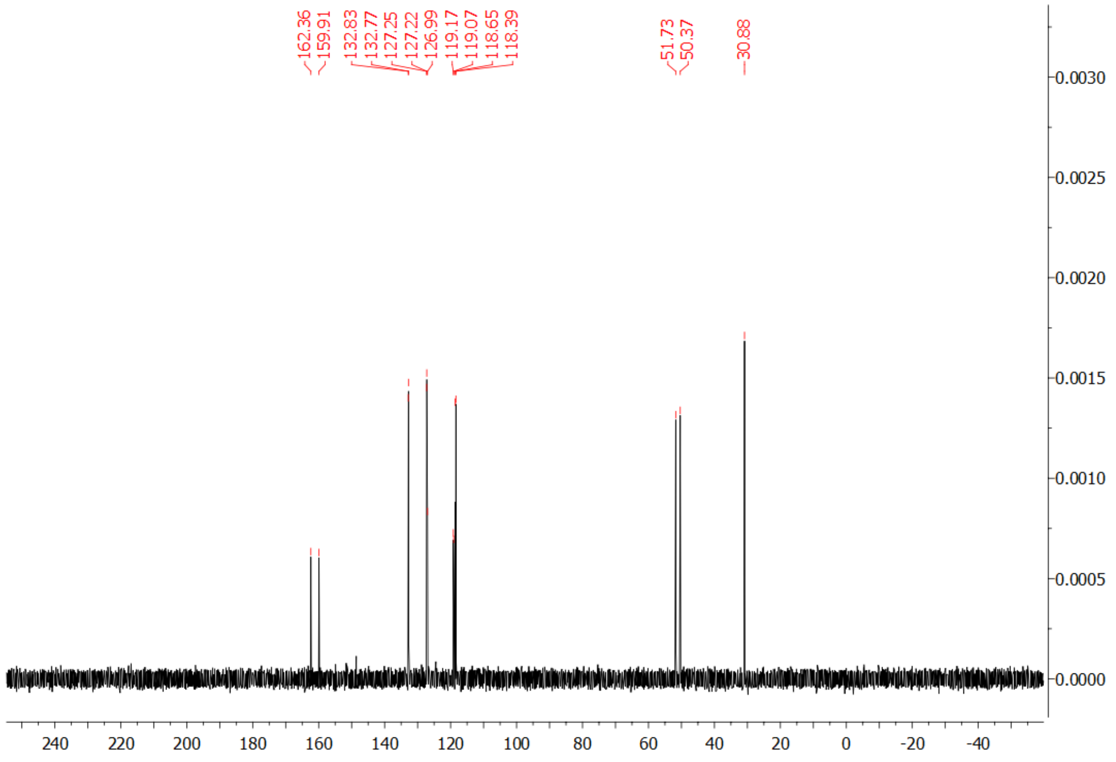

C

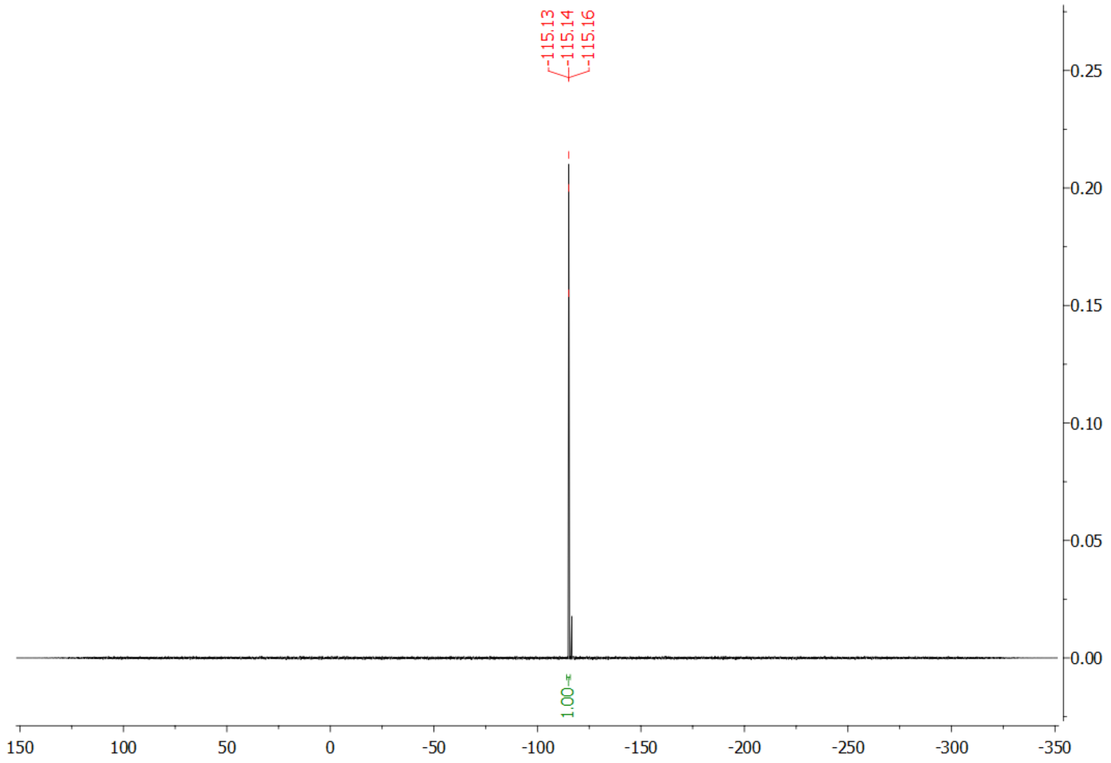

D

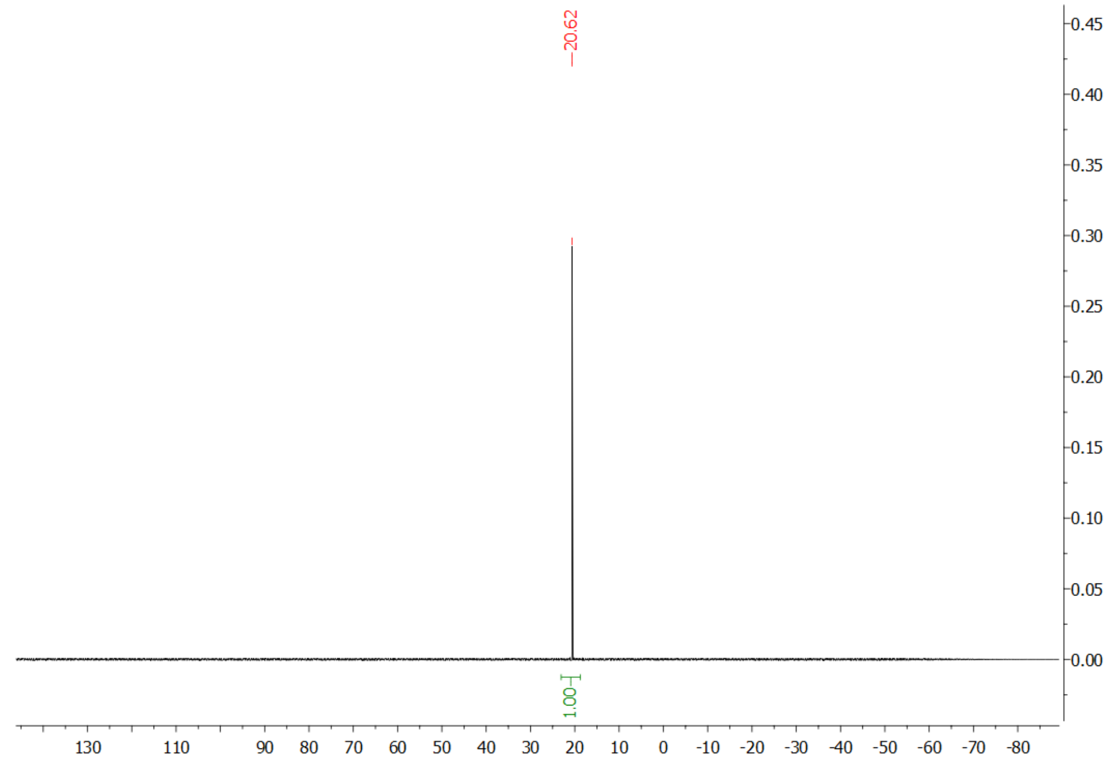

Figure S10-13. ^{1}H (**A**), ^{13}C (**B**), ^{19}F (**C**), ^{31}P (**D**) NMR spectra for compound **17e**.

A

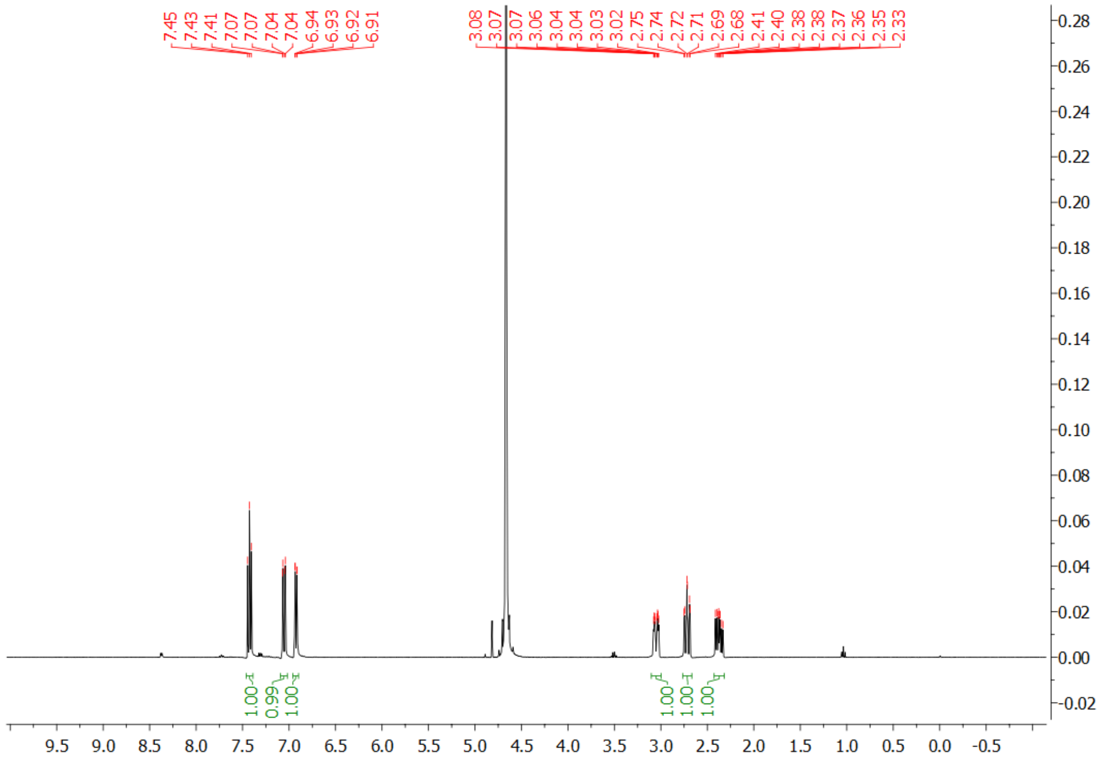

B

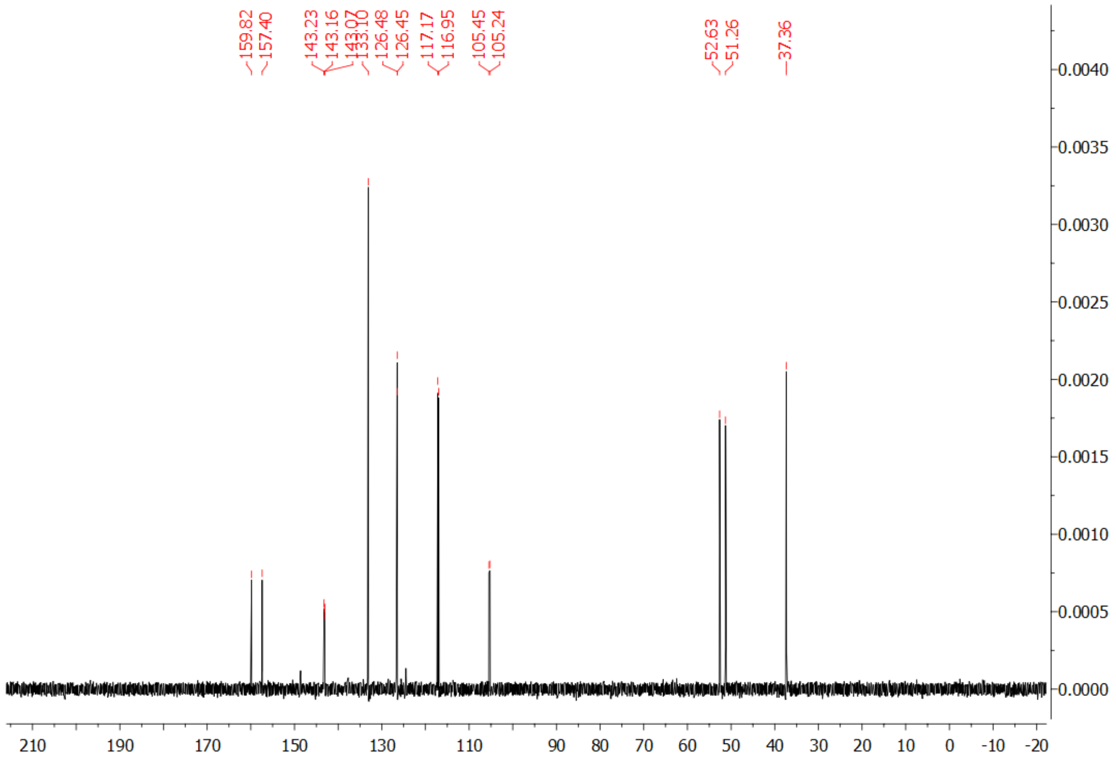

C

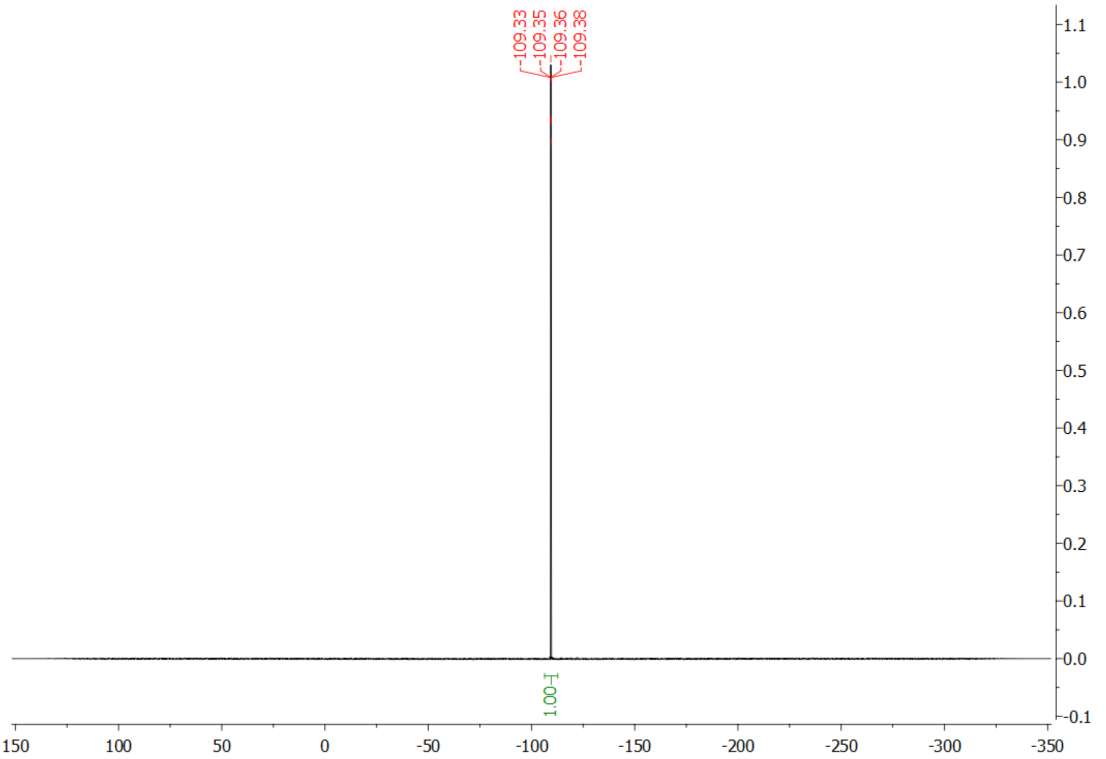
-109.33
-109.35
-109.36
-109.38
1.00
150
100
50
0
-50
-100
-150
-200
-250
-300
-350
1.1
1.0
0.9
0.8
0.7
0.6
0.5
0.4
0.3
0.2
0.1
0.0
-0.1

D

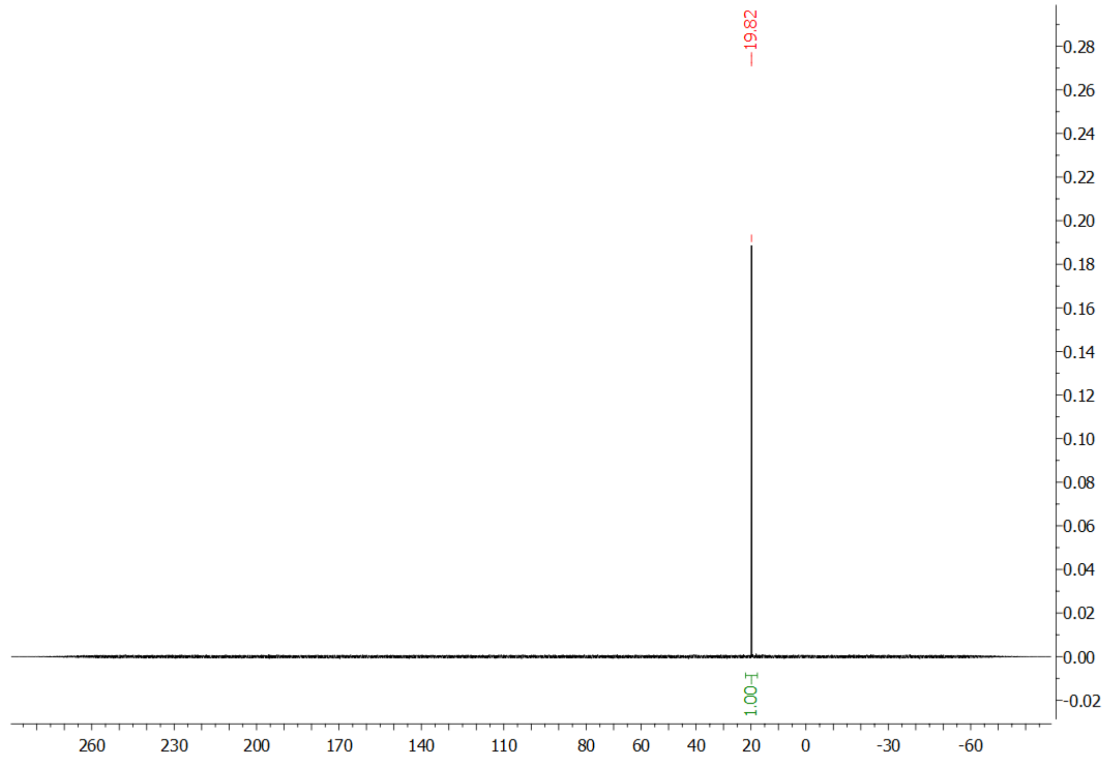
—19.82
1.00
260
230
200
170
140
110
80
60
40
20
0
-30
-60
0.28
0.26
0.24
0.22
0.20
0.18
0.16
0.14
0.12
0.10
0.08
0.06
0.04
0.02
0.00
-0.02

729 *References*

1. a) DeLong, M.A.; Amburgey, J.; Taylor, C.; Wos, J.A.; Soper, D.L.; Wang, Y.; Hicks, R. Synthesis and in vitro evaluation of human FP-receptor selective prostaglandin analogues. *Bioorg. Med. Chem. Lett.* **2000**, *10*, 1519–1522. https://doi.org/10.1016/S0960-894X(00)00273-0; b) Hamilton, G.S.; Wu, Y.-Q.; Limburg, D.C.; Wilkinson, D.E.; Vaal, M.J.; Li, J.-H.; Thomas, C.; Huang, W.; Sauer, H.; Ross, D.T.; et al. Synthesis of N -Glyoxyl Prolyl and Pipecolyl Amides and Thioesters and Evaluation of Their In Vitro and In Vivo Nerve Regenerative Effects. *J. Med. Chem.* **2002**, *45*, 3549–3557. https://doi.org/10.1021/jm010556c; c) Cao, W.; Liu, X.; Peng, R.; He, P.; Lin, L.; Feng, X. Catalytic asymmetric cross-dehydrogenative coupling: activation of C–H bonds by a cooperative bimetallic catalyst system. *Chem. Commun.* **2013**, *49*, 3470. https://doi.org/10.1039/c3cc41315b
2. Sanford, A.B.; Thane, T.A.; McGinnis, T.M.; Chen, P.-P.; Hong, X.; Jarvo, E.R. Nickel-Catalyzed Alkyl–Alkyl Cross-Electrophile Coupling Reaction of 1,3-Dimesylates for the Synthesis of Alkylcyclopropanes. *J. Am. Chem. Soc.* **2020**, *142*, 5017–5023. https://doi.org/10.1021/jacs.0c01330
3. Shimogaki, M.; Fujita, M.; Sugimura, T. Metal-Free Enantioselective Oxidative Arylation of Alkenes: Hypervalent-Iodine-Promoted Oxidative C–C Bond Formation. *Angew. Chemie Int. Ed.* **2016**, *55*, 15797–15801. https://doi.org/10.1002/anie.201609110
4. Lin, X.; Wang, Y.; Hu, Y.; Zhu, W.; Dou, X. Diboron-Mediated Rhodium-Catalysed Transfer Hydrogenation of Alkenes and Carbonyls. *European J. Org. Chem.* **2020**, *2020*, 1046–1049. https://doi.org/10.1002/ejoc.202000049
5. González-Sebastián, L.; Flores-Alamo, M.; García, J.J. Nickel-Catalyzed Reductive Hydroesterification of Styrenes Using CO 2 and MeOH. *Organometallics* **2012**, *31*, 8200–8207. https://doi.org/10.1021/om300819d
6. Woodward, D.F; Wang, J.W. Prostaglandin E receptor antagonists. *United States Patent Application Publication, US/2010/0256385 A1*, **2010**, Page/column 4
7. Chaumontet, M.; Piccardi, R.; Audic, N.; Hitce, J.; Peglion, J.-L.; Clot, E.; Baudoin, O. Synthesis of Benzocyclobutenes by Palladium-Catalyzed C−H Activation of Methyl Groups: Method and Mechanistic Study. *J. Am. Chem. Soc.* **2008**, *130*, 15157–15166. https://doi.org/10.1021/ja805598s
8. Gagnon, L.; Grouix, B. Substituted aromatic compounds and pharmaceutical compositions for the prevention and treatment of osteoporosis. *WO/2011/6054728 A1*, **2016,** Paragraph 00136
9. Chao, J.; Jain, R.; Hu. L.; Lewis, J.G.; Baribault, H.; Caldwell, J. Hormon receptor modulators for treating metabolic conditions and disorders, *WO/2018/039386 A1*, **2018**, Page/column 306
10. Eidam, H.S.; Raha, K.; Gong, Z.; Guan, H.; Wu, C.; Yang, H.; Yu, H.; Zhang, Z.; Cheung, M. Novel compounds as rearranged during transfection (RET) inhibitors. *United States Patent Application Publication US 2014/0275111 A1*, **2014**, Paragraph 0358; 0359
11. Cinelli, M.A.; Li, H.; Chreifi, G.; Martásek, P.; Roman, L.J.; Poulos, T.L.; Silverman, R.B. Simplified 2-Aminoquinoline-Based Scaffold for Potent and Selective Neuronal Nitric Oxide Synthase Inhibition. *J. Med. Chem.* **2014**, *57*, 1513–1530. https://doi.org/10.1021/jm401838x
12. Xu, G.-F.; Yang, X.-L.; Lei, P.; Liu, X.; Zhang, X.-B.; Ling, Y. Synthesis and fungicidal activity study of novel daphneolone analogs with 2,6-dimethylmorpholine. *Chinese Chem. Lett.* **2016**, *27*, 555–558. https://doi.org/10.1016/j.cclet.2016.01.045
13. Zhou, Y.; Li, Z.; Liu, Y.; Huo, J.; Chen, C.; Li, Q.; Niu, S.; Wang, S. Regulating Hydrogenation Chemoselectivity of α,β-Unsaturated Aldehydes by Combination of Transfer and Catalytic Hydrogenation. *ChemSusChem* **2020**, *13*, 1746–1750. https://doi.org/10.1002/cssc.201902629
14. Sibley, G.E.M.; Malmström, L.J.; Larsson, J.M. 2-amino-1,3,4-thiadazine and 2-amino-1,3,4-oxadiazine based antifungial agents. *WO/2017/009651 A1*, **2017**, Page column 159; 160
15. Desai, J.; Wang, Y.; Wang, K.; Malwal, S.R.; Oldfield, E. Isoprenoid Biosynthesis Inhibitors Targeting Bacterial Cell Growth. *ChemMedChem* **2016**, *11*, 2205–2215. https://doi.org/10.1002/cmdc.201600343
16. Chen, X.; Zhang, Y.; Wan, H.; Wang, W.; Zhang, S. Stereoselective organocatalytic oxidation of alcohols to enals: a homologation method to prepare polyenes. *Chem. Commun.* **2016**, *52*, 3532–3535. https://doi.org/10.1039/C5CC10093C
17. Gurak, J.A.; Engle, K.M. Practical Intermolecular Hydroarylation of Diverse Alkenes via Reductive Heck Coupling. *ACS Catal.* **2018**, *8*, 8987–8992. https://doi.org/10.1021/acscatal.8b02717

18. Farndon, J.J.; Ma, X.; Bower, J.F. Transition Metal Free C–N Bond Forming Dearomatizations and Aryl C–H Aminations by in Situ Release of a Hydroxylamine-Based Aminating Agent. *J. Am. Chem. Soc.* **2017**, *139*, 14005–14008. https://doi.org/10.1021/jacs.7b07830
19. Falck, J.R.; Paudyal, M.P.; Kürti, L. Direct C-H amination and Aza-annulation, *United States Patent Application Publication, US 2019/0152892 A1*, **2019**, Paragraph 0132; 0214; 0215
20. Wu, T.; Kang, X.; Bai, H.; Xiong, W.; Xu, G.; Tang, W. Enantioselective Construction of Spiro Quaternary Carbon Stereocenters via Pd-Catalyzed Intramolecular α-Arylation. *Org. Lett.* **2020**, *22*, 4602–4607. https://doi.org/10.1021/acs.orglett.0c01129
21. Yang, X.Y.; Lin, H.S.; Matsuo, Y. Highly Selective Synthesis of Tetrahydronaphthaleno[60]fullerenes via Fullerene-Cation-Mediated Intramolecular Cyclization. *J. Org. Chem.* **2019**, *84*, 16314–16322. https://doi.org/10.1021/acs.joc.9b02618
22. Xing, S.; Gu, N.; Wang, X.; Liu, J.; Xing, C.; Wang, K.; Zhu, B. Substitution-Controlled Selective Formation of Hexahydrobenz[e]isoindoles and 3-Benzazepines via In(OTf) 3 -Catalyzed Tandem Annulations. *Org. Lett.* **2018**, *20*, 5680–5683. https://doi.org/10.1021/acs.orglett.8b02406
23. Chen, S.; He, H.; Lagu, B.; Qin, H.; Wu, Ch.; Xiao, Y.; Tricyclic Sulfonamide derivatives, *WO/2015/102929A1*, **2015**, Page column 135-136
24. Brown, M.F.; Marfat, A.; Melnick, M.J.; Reilly, U. C-linked Hydroxamic acid derivatives useful as antibacterial agents. *WO/2011/045703 A2*, **2011**
25. Kuwada, T.; Yoshinaga, M.; Ishizaka, T.; Wakasugi, D.; Shirokawa, S.; Hattori, N.; Shimazaki, Y.; Miyakoshi, N. 1,2,4-Triazolone derivative. *United States Patent Application Publication, US 2013/0197217A1*, **2013**
26. Barda, D.A.; Henry, K.J.; Huang, J.; Joseph, S.; Lin, H-S.; Richett, M.E. 7-phenyl-isoquinoline-5-sulfonylamino derivatives as inhibitors of AKT (Proteinkinase B). *WO/2005/054202 A1*, **2005**, Page column 28
27. Uto, Y.; Ogata, T.; Harada, J.; Kiyotsuka, Y.; Ueno, Y.; Miyazawa, Y.; Kurata, H.; Deguchi, T.; Watanabe, N.; Takagi, T.; et al. Novel and potent inhibitors of stearoyl-CoA desaturase-1. Part I: Discovery of 3-(2-hydroxyethoxy)-4-methoxy-N-[5-(3-trifluoromethylbenzyl)thiazol-2-yl]benzamide. *Bioorg. Med. Chem. Lett.* **2009**, *19*, 4151–4158. https://doi.org/10.1016/j.bmcl.2009.05.119
28. Assaoui, H.; Boss, C.; Gude, M.; Koberstein, R.; Sifferlen, T. 5,6,7,8-tetrahydro-imidazo[1,5-A] pyrazine derivatives. *WO/2008/078291A1*, **2008**, Page/Page column 52
29. Matsumoto, T.; Katayama, N.; Mabuchi, H. Tyrosine phosphatase inhibitors. *United States Patent Application Publication, US 2003/0144338 A1*, **2003**
30. Chernyak, N.; Buchwald, S.L. Continuous-Flow Synthesis of Monoarylated Acetaldehydes Using Aryldiazonium Salts. *J. Am. Chem. Soc.* **2012**, *134*, 12466–12469. https://doi.org/10.1021/ja305660a
31. Baker, S.J.; Zhang, Y.-K.; Akama, T.; Lau, A.; Zhou, H.; Hernandez, V.; Mao, W.; Alley, M.R.K.; Sanders, V.; Plattner, J.J. Discovery of a New Boron-Containing Antifungal Agent, 5-Fluoro-1,3-dihydro-1-hydroxy-2,1- benzoxaborole (AN2690), for the Potential Treatment of Onychomycosis. *J. Med. Chem.* **2006**, *49*, 4447–4450. https://doi.org/10.1021/jm0603724
32. Huang, R.; Chen, X.; Mou, C.; Luo, G.; Li, Y.; Li, X.; Xue, W.; Jin, Z.; Chi, Y.R. Carbene-Catalyzed α-Carbon Amination of Chloroaldehydes for Enantioselective Access to Dihydroquinoxaline Derivatives. *Org. Lett.* **2019**, *21*, 4340–4344. https://doi.org/10.1021/acs.orglett.9b01520
33. Houjeiry, T.I.; Poe, S.L.; McQuade, D.T. Synthesis of Optically Active 4-Substituted 2-Cyclohexenones. *Org. Lett.* **2012**, *14*, 4394–4397. https://doi.org/10.1021/ol301874x

© 2020 by the authors. Submitted for possible open access publication under the terms and conditions of the Creative Commons Attribution (CC BY) license (http://creativecommons.org/licenses/by/4.0/).
